# Establishment and Temporal Validation of Next-Generation Reference Intervals for Routine Hematological Parameters Using Large-Scale Data

**DOI:** 10.3390/diagnostics16060944

**Published:** 2026-03-23

**Authors:** Chaochao Ma, Lihua Guan, Qian Chen, Rongrong Cheng, Wei Wu, Ling Qiu

**Affiliations:** 1 Department of Laboratory Medicine, Peking Union Medical College Hospital, Peking Union Medical College & Chinese Academy of Medical Science, Beijing 100730, China; machaochao@pumch.cn (C.M.); lihua180401@163.com (L.G.); chenq0806@163.com (Q.C.); chengrongrong2026@163.com (R.C.); 2 Department of Occupational and Environmental Health Sciences, School of Public Health, Peking University, Beijing 100191, China; 3 State Key Laboratory of Complex Severe and Rare Diseases, Peking Union Medical College Hospital, Peking Union Medical College & Chinese Academy of Medical Science, Beijing 100730, China

**Keywords:** next-generation reference intervals, hematological parameters, GAMLSS, big data, age-continuous modeling, temporal validation

## Abstract

**Background**: Conventional reference intervals (RIs) are typically expressed as fixed limits and may not adequately reflect continuous biological variation across age and sex. Next-generation reference intervals (NGRIs) allow dynamic modeling of laboratory parameters across the lifespan. This study aimed to establish age- and sex-specific NGRIs for routine hematological parameters using large-scale health examination data and to evaluate their temporal stability. **Methods**: Health examination records were linked with laboratory data, and a relatively healthy reference population was defined based on age (18–80 years), normal body mass index, normal blood pressure, and absence of documented disease history. NGRIs were constructed using generalized additive models for location, scale, and shape (GAMLSS) with the Box–Cox Cole and Green distribution. Age-dependent percentile curves (2.5th–97.5th) were generated using bootstrap resampling (100 iterations). Temporal external validation was conducted in five independent annual cohorts (2019–2023). **Results**: Age- and sex-dependent distributional patterns were observed across multiple hematological parameters, particularly RBC, HGB, and HCT. Continuous percentile curves demonstrated gradual age-related trends, with more pronounced changes in females after midlife. In temporal validation cohorts, the proportion of individuals classified outside the reference intervals remained consistently below 10% across years and sexes, indicating stable performance. **Conclusions**: Using large-scale real-world health examination data and a flexible distributional modeling framework, we established stable age-continuous next-generation reference intervals for routine hematological parameters. The proposed approach provides a reproducible strategy for modernizing laboratory reference interval construction and supports broader implementation in population-based laboratory medicine.

## 1. Introduction

Reference intervals (RIs) are essential tools in clinical laboratory medicine, serving as the basis for interpreting laboratory test results and supporting clinical decision-making [1,2,3]. Conventionally, first-generation reference intervals are established using a predefined “healthy” reference population and are typically expressed as fixed lower and upper limits. Although widely used, these single, static reference intervals do not account for continuous biological variation across age or other physiological factors. Such simplification may lead to reduced diagnostic accuracy, particularly for biomarkers that change dynamically throughout the lifespan [4].

To address these limitations, next-generation reference intervals (NGRIs) have been proposed. Unlike conventional RIs, NGRIs model biomarker distributions as continuous functions of covariates such as age, allowing reference limits to vary smoothly across the lifespan [4]. This approach provides a more accurate characterization of age-related trends in laboratory parameters [5,6,7,8]. In addition, standardized modeling frameworks enable transformation of results into dimensionless indices, thereby reducing dependence on measurement units and improving comparability [4]. In recent years, methodological advances—particularly in distributional modeling and large-scale data analytics—have facilitated the development of data-driven, age-specific reference intervals [9,10,11,12,13,14,15]. However, studies constructing NGRIs using real-world large-scale health examination data remain limited.

Against this background, the present study leveraged large-scale health examination data to establish NGRIs for routine hematological parameters. Using advanced distributional modeling techniques, we developed age- and sex-specific continuous reference curves and further evaluated their performance through temporal validation in independent annual cohorts. This study provides both methodological insight and empirical evidence for constructing next-generation reference intervals based on real-world big data.

## 2. Materials and Methods

### 2.1. Study Design and Data Sources

**Figure 1:**
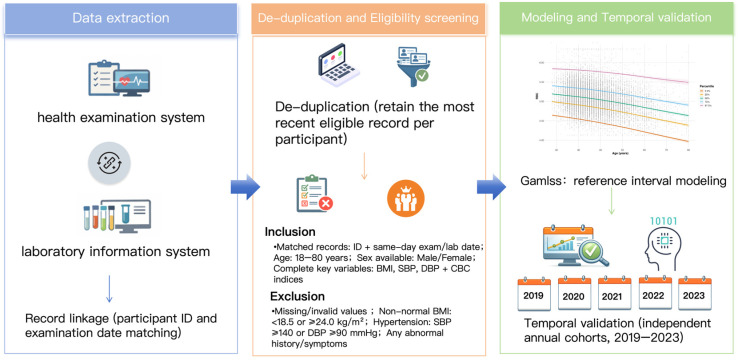
Overall Study Design and Analytical Workflow.

This study was a retrospective observational analysis based on routinely collected data from an established health examination system, integrating health check-up records with laboratory test results. Individual-level information was obtained from the health examination database, and hematological test indices were extracted from the laboratory information system; the two datasets were linked and consolidated at the data level. The overall study workflow is shown in Figure 1. To enable model development and external validation across time, a time-split strategy was adopted: health examination data from 2014 to 2018 were used as the training dataset, and five independent validation datasets were constructed by calendar year from 2019 to 2023, with each dataset representing an annual cohort from the same center (Peking Union Medical College Hospital). All data were de-identified prior to statistical analysis. This study relied solely on existing records and did not alter or intervene in routine clinical or health examination practice. Before modeling, we first applied predefined inclusion and exclusion criteria based on clinical and health examination information to define a relatively healthy population. Inclusion criteria were matched records with an available ID and a same-day examination/laboratory date, age 18–80 years, available sex information, and complete key variables including BMI, SBP, DBP, and CBC indices. Exclusion criteria included missing or invalid values, non-normal BMI, hypertension, and any abnormal history or symptoms recorded during the health examination. Because relevant clinical information was collected during routine examinations, individuals with chronic inflammatory conditions, iron deficiency, or other relevant abnormalities would have been excluded if these conditions were identified and documented (Figure 1). 

This flowchart illustrates the overall study workflow, including data extraction, de-duplication and eligibility screening, reference interval modeling, and temporal validation using independent annual cohorts.

### 2.2. Data Cleaning

#### 2.2.1. Record Linkage

Health examination records were deterministically linked to laboratory test results using unique personal identifiers and examination dates. The examination date was aligned with the laboratory specimen receipt date to ensure same-day matching. Only records with concordant identifiers and dates were retained for further analysis.

#### 2.2.2. De-Duplication Strategy

To ensure independence of observations, only one record per individual was retained. For the training dataset, when multiple examinations were available, the most recent eligible record was selected. For annual validation cohorts (2019–2023), de-duplication was performed within each calendar year, retaining one record per individual per year.

#### 2.2.3. Variables and Units

The primary hematological indices included white blood cell count (WBC, ×10^9^/L), red blood cell count (RBC, ×10^12^/L), hemoglobin (HGB, g/L), hematocrit (HCT, %), mean corpuscular volume (MCV, fL), mean corpuscular hemoglobin (MCH, pg), mean corpuscular hemoglobin concentration (MCHC, g/L), and platelet count (PLT, ×10^9^/L). Demographic and clinical variables included age (years), sex, body mass index (BMI, kg/m^2^), systolic blood pressure (mmHg), and diastolic blood pressure (mmHg).

Age was restricted to 18–80 years. Sex was coded as a binary variable. Laboratory values were converted to numeric format, and non-numeric characters (e.g., “<”, “>”) were removed prior to analysis. Values that could not be converted, as well as implausible values—defined here as rare erroneous results such as zero or negative values, most likely caused by instrument or data-recording errors—were treated as missing.

### 2.3. Analytical Platform

Hematological measurements were performed using automated hematology analyzers (Sysmex XE-5000, XE-5100, and Sysmex XN-9100; Sysmex Corporation, Kobe, Japan). During the study period, different analyzer platforms were used sequentially. Inter-platform comparability was ensured through routine method comparison procedures conducted by the laboratory. When a new analyzer was introduced, method comparison and validation studies were performed in accordance with internal quality management protocols. Only after demonstrating acceptable agreement between platforms was the new analyzer placed into routine clinical use.

### 2.4. Quality Control

Internal quality control was performed daily using commercial control materials at multiple concentration levels in accordance with the manufacturer’s recommendations. Quality control results were monitored using established laboratory quality rules to ensure analytical stability and precision. The laboratory participated in an external quality assessment program organized by national or regional proficiency testing providers. External quality assessment performance was reviewed periodically to verify inter-laboratory comparability and long-term analytical accuracy. All instruments were calibrated and maintained according to the manufacturer’s instructions and laboratory standard operating procedures. Only test results generated under routine laboratory quality assurance, with internal quality control and external quality assessment conducted according to standard practice, were included in the final analysis.

In addition to analytical quality control, data processing and statistical modeling were conducted using standardized and version-controlled scripts written in R. All data cleaning, transformation, and modeling procedures were predefined and automated to minimize manual intervention. Code validation was performed through stepwise verification of intermediate outputs, consistency checks of key variables, and repeated bootstrap procedures to ensure computational stability. Random seeds were set where applicable to guarantee reproducibility of results. Independent cross-checking of output tables and graphical results was performed prior to final reporting.

### 2.5. Statistical Analysis and Modeling

All statistical analyses were performed using R (R Foundation for Statistical Computing, Vienna, Austria) [16] and relevant packages, including dplyr [17], tidyr [18], stringr [19], lubridate (data cleaning and manipulation) [20], readr [21]/readxl [22]/purrr (data import and batch processing) [23], gamlss [24]/gamlss.dist [25]/gamlss.add [26] and mgcv (age-continuous modeling and smoothing) [16,27,28,29], ggplot2 [30] and scales (visualization) [31], and parallel (parallelized bootstrap computation) [16]. Continuous variables were summarized as the median and interquartile range. Normality was assessed using the Kolmogorov–Smirnov test. Between-group comparisons were conducted using the Wilcoxon rank-sum test with Benjamini–Hochberg adjustment for multiple testing where applicable.

NGRIs were constructed using the GAMLSS framework. For each hematological parameter and sex, age-specific distributions were modeled using the Box–Cox Cole and Green distribution. The location (μ) and scale (σ) parameters were modeled as smooth functions of age using penalized B-splines, while the shape parameter (ν) was modeled as a constant.

To enhance robustness and reduce the influence of sampling variability, a bootstrap procedure was implemented. For each parameter and sex, eligible observations were modeled repeatedly (100 iterations). Age-specific percentile curves (2.5th, 25th, 50th, 75th, and 97.5th percentiles) were generated from each fitted model. The final reference estimates were calculated as the mean across bootstrap iterations, and 90% confidence intervals were derived from the 5th and 95th percentiles of the bootstrap distribution.

Prior to modeling, laboratory values were winsorized at the 0.5th and 99.5th percentiles to reduce the influence of extreme outliers. This was done to reduce the influence of a very small number of extreme values that might reflect residual measurement or recording errors, or unrecognized conditions not captured by the exclusion criteria.

For temporal external validation, age- and sex-specific reference limits (2.5th and 97.5th percentiles) derived from the training dataset were applied to each independent annual cohort (2019–2023). Individuals were classified as below reference interval (L), within reference interval, or above reference interval (U), and proportions were calculated by sex and year.

A two-sided *p* value < 0.05 was considered statistically significant.

## 3. Results

### 3.1. Baseline Characteristics of the Training and Validation Datasets

A total of 47,093 participants were included in the training dataset (2014–2018), and 74,964 participants were included across five independent validation datasets collected from 2019 to 2023 (validation datasets 1–5: 17,896; 9234; 14,571; 13,426; and 14,837, respectively) (Table 1). The median age was similar across datasets, ranging from 35 years to 36 years. The proportion of males was 32.2% in the training dataset and was higher in the validation datasets (37.4–40.3%). Median BMI was comparable across cohorts. Blood pressure distributions were also consistent, with median SBP around 109–111 mmHg and median DBP around 69–70 mmHg.

### 3.2. Establishment of Next-Generation Reference Interval Models for Hematological Parameters

**Figure 2:**
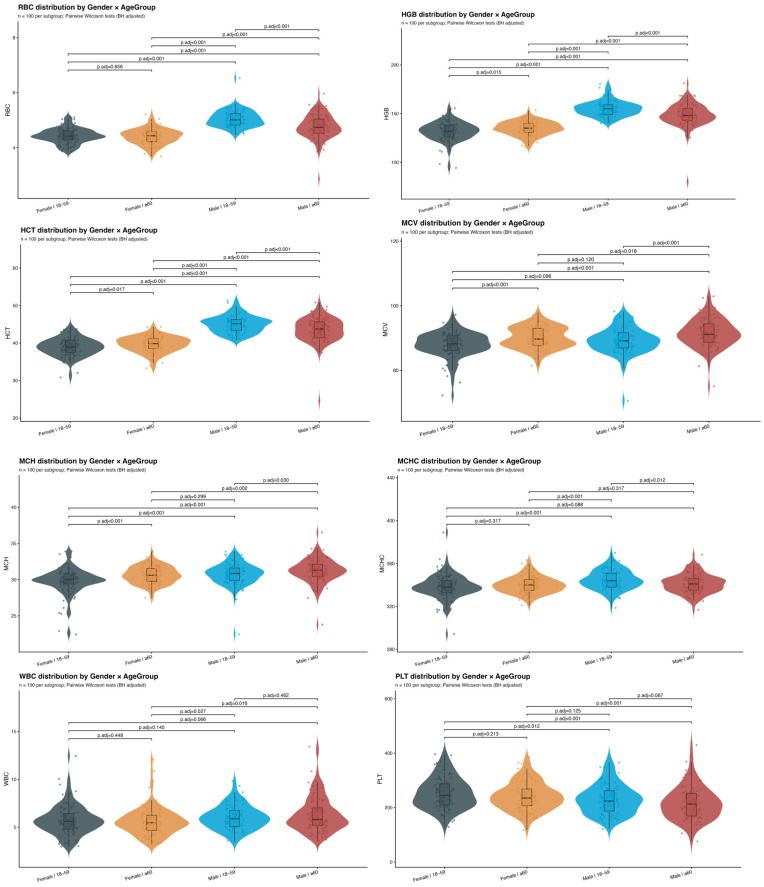
Distribution of hematological parameters stratified by sex and age group. Note: Violin plots showing the distributions of hematological parameters stratified by sex and age group. Participants were grouped by sex (female and male) and age (18–59 years and ≥60 years). Pairwise comparisons between subgroups were performed using Wilcoxon rank-sum tests with Benjamini–Hochberg adjustment for multiple testing.

The distributions of RBC, HGB, HCT, MCV, MCH, MCHC, WBC, and PLT stratified by sex and age group are presented in Figure 2. Overall, significant sex-related differences were observed for RBC, HGB, HCT, MCV, MCH, and PLT (all *p* < 0.05), indicating systematic separation between male and female measurements across the study population. In contrast, the sex effect for MCHC was more age-dependent: a significant difference between non-elderly males and females was detected (*p* < 0.05), whereas the corresponding comparison in the elderly group did not reach statistical significance. For WBC, a significant sex difference was observed specifically in the elderly subgroup (*p* < 0.05), while the non-elderly male–female comparison was not statistically significant.

Based on these distributional patterns and subgroup differences, next-generation reference interval models were constructed for each hematological parameter to generate age- and sex-specific reference limits. For RBC, the model-predicted reference intervals across age for females and males are summarized in Table 2. Corresponding model outputs for HGB, HCT, MCV, MCH, MCHC, WBC, and PLT are provided in Supplemental Tables S1–S7.

**Figure 3:**
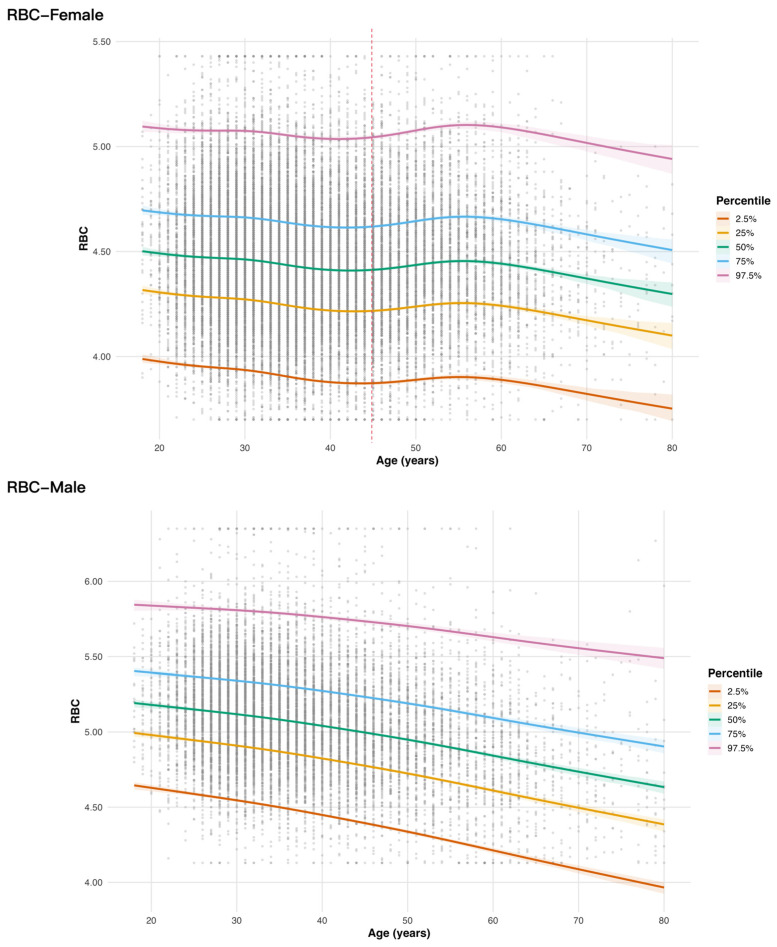
Age-specific next-generation reference intervals for red blood cell count stratified by sex. Note: Grey dots represent the raw data. The pink dashed line indicates an inflection point for RBC, and the same applies to HCT and HGB in females only. Estimated percentile curves (2.5th–97.5th) were obtained using GAMLSS, with shaded areas representing 90% bootstrap confidence intervals.

Model predictions further suggested an age-related downward shift in RBC reference limits. Specifically, RBC reference interval limits decreased with increasing age, with a more pronounced decline in females after approximately 50 years of age, whereas in males a gradual decline was observed throughout the age range (Figure 3). The age-specific NGRI curves for the remaining parameters (HGB, HCT, MCV, MCH, MCHC, WBC, and PLT) are shown in Supplemental Figures S1–S7.

### 3.3. Validation of Next-Generation Reference Intervals

As shown in Table 3, the next-generation reference interval (NGRI) for RBC demonstrated consistent performance across five independent annual validation cohorts collected from 2019 to 2023. After stratification by calendar year and sex, the proportion of individuals classified outside the reference interval (i.e., L + U) remained below 10% in both females and males in every validation cohort, while the vast majority were classified as within-range (N), accounting for approximately 94–95% each year. These findings indicate good external reproducibility and generalizability of the RBC NGRI across temporally independent cohorts.

Similarly, validation analyses for HGB, HCT, MCV, MCH, MCHC, WBC, and PLT showed that, for each year from 2019 to 2023 and for both sexes, the proportion of observations outside the corresponding NGRIs was consistently <10% (Supplementary Tables S8–S14).

## 4. Discussion

In this large-scale health examination study, we established sex- and age-specific NGRIs for routine hematological parameters using a GAMLSS-based modeling framework and evaluated their temporal stability across five independent annual validation cohorts. The models demonstrated clear age- and sex-dependent distributional patterns. Importantly, external validation showed that the proportion of individuals classified outside the reference limits remained consistently below 10% across calendar years and sexes, indicating good reproducibility and generalizability of the proposed NGRIs. These findings support the feasibility of constructing stable and clinically meaningful continuous reference intervals from real-world health examination data.

The larger cumulative size of the validation datasets was mainly attributable to the different de-duplication strategies used in the training and validation phases. Specifically, only one eligible record per individual was retained across the entire training period, whereas one record per individual was retained within each validation year. This strategy also contributed to the difference in sex composition between the training and validation datasets. However, this did not affect the study results, because both model development and validation were performed separately for females and males.

Sex- and age-related differences in hematological parameters are well documented. Previous population-based studies have consistently shown higher RBC and HGB levels in males compared with females, largely attributable to androgen stimulation of erythropoiesis and menstrual blood loss in premenopausal women [32,33]. Age-related declines in RBC and HGB have also been reported, particularly after midlife, reflecting physiological changes in bone marrow function and hormonal regulation [34]. The age-related pattern of RBC reference limits observed in our study, with a noticeable inflection around approximately 45 years of age in females, may be related to the menopausal transition and age-related changes in hematopoiesis [35]. Our findings further corroborate that sex stratification and continuous age modeling are essential for accurate interpretation of hematological parameters. Methodologically, this study adopted a data-driven approach based on a large health examination population. By applying strict eligibility criteria—including normal BMI, blood pressure, and absence of documented disease history—we derived a relatively healthy reference cohort from real-world screening data. Compared with traditional direct sampling methods requiring prospective recruitment, this indirect large-scale strategy provides greater statistical power and improved representativeness.

The GAMLSS framework enabled flexible modeling of age-dependent changes in both location and scale parameters using penalized B-splines. Compared with fixed partitioning by age groups, this approach avoids arbitrary cut-offs and allows smooth transitions across the lifespan. Bootstrap resampling further enhanced model robustness and provided uncertainty estimates for percentile curves. Alternative indirect approaches, such as kosmic [36] and refineR [37] combined with smoothing techniques, have also been proposed for large laboratory databases. However, refineR primarily focuses on extracting reference distributions from mixed populations without explicit covariate modeling. In contrast, the GAMLSS-based strategy applied here directly incorporates age as a continuous covariate, enabling more precise characterization of distributional shifts over time. Similar to RBC, both HGB and HCT showed a noticeable fluctuation after approximately 45 years of age in females. It is worth noting that, under the current inclusion and exclusion criteria, older individuals may have been more likely to be excluded, which could have resulted in a lower sample density at older ages and consequently wider 90% confidence intervals in this age range. Although the number of participants decreased at the upper end of the age range, the GAMLSS framework models age as a continuous variable using smooth functions across the full dataset within each sex. Together with bootstrap-based uncertainty estimation and winsorization of extreme values, this supports the robustness of the overall NGRI curves, while also indicating that estimates at the oldest ages should be interpreted with appropriate caution.

External validation can generally be performed in several ways, including validation across different time periods, different clinical settings, or different geographic locations. In the present study, the validation cohorts were derived from the same center but from different calendar years; therefore, our approach represents external validation across time, that is, temporal external validation, rather than validation across different institutions or regions. External temporal validation demonstrated consistent classification performance across five independent annual cohorts from 2019 to 2023. In each year and for both sexes, the proportion of individuals outside the RBC NGRI remained below 10%, with approximately 94–95% classified within the reference interval. Similar findings were observed for the other hematological parameters. These results indicate strong temporal stability and support the generalizability of the constructed models across different calendar years, despite minor demographic shifts between cohorts. Temporal validation is particularly important in large-scale real-world datasets, where changes in analyzer platforms, population structure, or healthcare utilization patterns may influence laboratory distributions. The stable validation performance observed in this study suggests that the modeling framework is robust to such real-world variability.

It should also be noted that RDW and PDW were not included in the present analysis because this study focused on core routine hematological parameters with broader clinical use and better inter-platform comparability. As distribution-width indices, RDW and PDW may be more sensitive to analyzer-specific measurement algorithms and instrument-related variation.

From an implementation perspective, the NGRIs derived in this study are fundamentally continuous age- and sex-specific reference curves, because age was modeled as a continuous variable in the GAMLSS framework. In practice, these dynamic limits could be implemented in laboratory information systems either by directly embedding the underlying model to generate individualized reference limits at the time of reporting, or by converting the continuous curves into more granular age-specific lookup tables that remain substantially more refined than the broad age brackets currently used in routine practice.

This study has several strengths. First, it was based on a large health examination population, providing substantial statistical power and minimizing random variation. Second, the indirect healthy cohort selection strategy enabled construction of reference intervals in a cost-effective and scalable manner. Third, the combination of GAMLSS modeling and bootstrap validation enhanced methodological rigor and stability.

However, several limitations should be acknowledged. This was a single-center study, and although temporal validation was performed, geographic generalizability remains to be established. Future multi-center studies incorporating diverse populations and laboratory systems would further strengthen the external validity of next-generation reference intervals. Furthermore, although we applied multiple exclusion criteria to define a relatively healthy reference population, this indirect approach may not completely exclude individuals with subclinical or undiagnosed conditions. As a result, some latent health abnormalities may still have influenced the estimated reference distributions. Lastly, the final RI limits, particularly for highly skewed parameters, may be somewhat influenced by the choice of truncation thresholds used for winsorization. In this study, the 0.5th and 99.5th percentiles were selected as a conservative approach to limit the impact of a very small number of extreme values on model fitting. Future studies may further evaluate the robustness of the estimated limits by formally comparing alternative truncation schemes.

## 5. Conclusions

This study demonstrates that large-scale health examination data combined with flexible distributional modeling can generate stable, age-continuous next-generation reference intervals. The proposed framework provides a practical and reproducible strategy for modernizing laboratory reference interval construction.

## Figures and Tables

**Table 1:** Participant baseline characteristics in the training and validation datasets.

Characteristic	Training Dataset	Validation Dataset 1	Validation Dataset 2	Validation Dataset 3	Validation Dataset 4	Validation Dataset 5
N	47,093	17,896	9234	14,571	13,426	14,837
Age (Year)	35 (30, 44)	35 (30–43)	36 (30–43)	35 (30–42)	36 (30–43)	36 (30–43)
Gender (Male%)	32.2	40.1	40.3	39.0	38.5	37.4
Gender (Female%)	67.8	59.9	59.7	61.0	61.5	62.6
BMI (kg/m^2^)	21.6 (20.3, 22.8)	21.8 (20.4–22.9)	21.8 (20.4–22.9)	21.7 (20.4–22.9)	21.7 (20.3–22.9)	21.7 (20.3–22.9)
SBP (mmHg)	110 (102, 119)	111.0 (105.0–120.0)	110.0 (105.0–118.0)	109.0 (104.0–117.0)	110.0 (104.0–117.0)	110.0 (105.0–118.0)
DBP (mmHg)	69 (63.0–75.0)	69.0 (64.0–75.0)	70.0 (65.0–75.0)	69.0 (64.0–74.0)	69.0 (64.0–74.0)	69.0 (64.0–74.0)

Note: Data are summarized as the median (interquartile range) for continuous variables and as percentages for categorical variables. The training set included health examination records collected from 2014 to 2018. Validation datasets 1–5 were independently assembled from health examination cohorts collected from 2019 to 2023, with each dataset representing one calendar year. BMI denotes body mass index; SBP, systolic blood pressure; DBP, diastolic blood pressure.

**Table 2:** Model-predicted sex- and age-specific next-generation reference intervals for RBC.

Age	Female					Male				
	Q2.5	Q25	Q50	Q75	Q97.5	Q2.5	Q25	Q50	Q75	Q97.5
18	3.99 [3.97–4.01]	4.32 [4.30–4.34]	4.50 [4.48–4.52]	4.70 [4.67–4.72]	5.10 [5.07–5.12]	4.65 [4.62–4.67]	4.99 [4.97–5.01]	5.19 [5.16–5.22]	5.40 [5.37–5.43]	5.84 [5.80–5.88]
19	3.98 [3.97–4.00]	4.31 [4.29–4.33]	4.50 [4.48–4.51]	4.69 [4.67–4.71]	5.09 [5.07–5.12]	4.64 [4.61–4.66]	4.99 [4.96–5.01]	5.19 [5.16–5.21]	5.40 [5.37–5.42]	5.84 [5.80–5.87]
20	3.98 [3.96–3.99]	4.31 [4.29–4.32]	4.49 [4.48–4.51]	4.69 [4.67–4.70]	5.09 [5.07–5.11]	4.63 [4.61–4.65]	4.98 [4.96–5.00]	5.18 [5.16–5.20]	5.39 [5.37–5.41]	5.84 [5.81–5.87]
21	3.97 [3.96–3.98]	4.30 [4.29–4.31]	4.49 [4.47–4.50]	4.68 [4.67–4.70]	5.08 [5.07–5.10]	4.62 [4.60–4.64]	4.97 [4.96–4.99]	5.17 [5.15–5.19]	5.39 [5.37–5.41]	5.84 [5.81–5.86]
22	3.97 [3.96–3.98]	4.30 [4.29–4.30]	4.48 [4.47–4.49]	4.68 [4.67–4.69]	5.08 [5.06–5.10]	4.61 [4.59–4.63]	4.97 [4.95–4.98]	5.17 [5.15–5.18]	5.38 [5.36–5.40]	5.83 [5.81–5.86]
23	3.96 [3.95–3.97]	4.29 [4.28–4.30]	4.48 [4.47–4.49]	4.67 [4.67–4.68]	5.08 [5.06–5.09]	4.60 [4.59–4.62]	4.96 [4.95–4.97]	5.16 [5.15–5.17]	5.38 [5.36–5.39]	5.83 [5.81–5.85]
24	3.96 [3.95–3.97]	4.29 [4.28–4.30]	4.48 [4.47–4.48]	4.67 [4.66–4.68]	5.08 [5.06–5.09]	4.60 [4.58–4.61]	4.95 [4.94–4.96]	5.16 [5.14–5.17]	5.37 [5.36–5.39]	5.83 [5.81–5.85]
25	3.95 [3.94–3.96]	4.29 [4.28–4.29]	4.47 [4.47–4.48]	4.67 [4.66–4.68]	5.08 [5.06–5.09]	4.59 [4.58–4.60]	4.95 [4.94–4.96]	5.15 [5.14–5.16]	5.37 [5.36–5.38]	5.82 [5.81–5.84]
26	3.95 [3.94–3.96]	4.28 [4.28–4.29]	4.47 [4.47–4.48]	4.67 [4.66–4.67]	5.08 [5.06–5.09]	4.58 [4.57–4.59]	4.94 [4.93–4.95]	5.14 [5.14–5.15]	5.36 [5.35–5.37]	5.82 [5.80–5.84]
27	3.95 [3.94–3.95]	4.28 [4.28–4.28]	4.47 [4.46–4.47]	4.67 [4.66–4.67]	5.08 [5.06–5.08]	4.57 [4.56–4.58]	4.93 [4.92–4.94]	5.14 [5.13–5.15]	5.36 [5.35–5.36]	5.82 [5.80–5.83]
28	3.94 [3.94–3.95]	4.28 [4.27–4.28]	4.47 [4.46–4.47]	4.67 [4.66–4.67]	5.08 [5.07–5.09]	4.56 [4.55–4.57]	4.92 [4.92–4.93]	5.13 [5.13–5.14]	5.35 [5.34–5.36]	5.81 [5.80–5.83]
29	3.94 [3.93–3.95]	4.28 [4.27–4.28]	4.47 [4.46–4.47]	4.66 [4.66–4.67]	5.08 [5.07–5.08]	4.55 [4.54–4.56]	4.92 [4.91–4.92]	5.12 [5.12–5.13]	5.35 [5.34–5.35]	5.81 [5.80–5.83]
30	3.94 [3.93–3.94]	4.27 [4.27–4.28]	4.46 [4.46–4.47]	4.66 [4.66–4.67]	5.07 [5.07–5.08]	4.55 [4.54–4.56]	4.91 [4.90–4.91]	5.12 [5.11–5.12]	5.34 [5.34–5.35]	5.81 [5.79–5.82]
31	3.93 [3.92–3.94]	4.27 [4.26–4.27]	4.46 [4.45–4.46]	4.66 [4.65–4.67]	5.07 [5.06–5.08]	4.54 [4.53–4.55]	4.90 [4.90–4.91]	5.11 [5.11–5.12]	5.34 [5.33–5.34]	5.80 [5.79–5.82]
32	3.93 [3.92–3.93]	4.26 [4.26–4.27]	4.45 [4.45–4.46]	4.65 [4.65–4.66]	5.07 [5.06–5.08]	4.53 [4.52–4.54]	4.89 [4.89–4.90]	5.10 [5.10–5.11]	5.33 [5.32–5.34]	5.80 [5.79–5.81]
33	3.92 [3.91–3.92]	4.26 [4.25–4.26]	4.45 [4.44–4.45]	4.65 [4.64–4.65]	5.06 [5.06–5.07]	4.52 [4.51–4.53]	4.89 [4.88–4.89]	5.10 [5.09–5.10]	5.32 [5.32–5.33]	5.80 [5.78–5.81]
34	3.91 [3.91–3.92]	4.25 [4.25–4.25]	4.44 [4.44–4.45]	4.64 [4.64–4.65]	5.06 [5.05–5.07]	4.51 [4.50–4.52]	4.88 [4.87–4.88]	5.09 [5.08–5.10]	5.32 [5.31–5.32]	5.79 [5.78–5.81]
35	3.90 [3.90–3.91]	4.24 [4.24–4.25]	4.44 [4.43–4.44]	4.64 [4.63–4.64]	5.05 [5.05–5.06]	4.50 [4.49–4.51]	4.87 [4.86–4.88]	5.08 [5.08–5.09]	5.31 [5.30–5.32]	5.79 [5.78–5.80]
36	3.90 [3.89–3.90]	4.24 [4.23–4.24]	4.43 [4.42–4.43]	4.63 [4.63–4.64]	5.05 [5.04–5.06]	4.49 [4.48–4.50]	4.86 [4.85–4.87]	5.07 [5.07–5.08]	5.30 [5.30–5.31]	5.78 [5.77–5.80]
37	3.89 [3.88–3.90]	4.23 [4.23–4.24]	4.42 [4.42–4.43]	4.63 [4.62–4.63]	5.04 [5.03–5.06]	4.48 [4.47–4.49]	4.85 [4.84–4.86]	5.07 [5.06–5.07]	5.30 [5.29–5.30]	5.78 [5.77–5.79]
38	3.89 [3.88–3.89]	4.23 [4.22–4.23]	4.42 [4.41–4.42]	4.62 [4.62–4.63]	5.04 [5.03–5.05]	4.47 [4.46–4.48]	4.84 [4.84–4.85]	5.06 [5.05–5.06]	5.29 [5.28–5.30]	5.77 [5.76–5.79]
39	3.88 [3.87–3.89]	4.22 [4.22–4.23]	4.42 [4.41–4.42]	4.62 [4.61–4.62]	5.04 [5.03–5.05]	4.46 [4.45–4.47]	4.83 [4.83–4.84]	5.05 [5.04–5.06]	5.28 [5.27–5.29]	5.77 [5.75–5.78]
40	3.88 [3.87–3.88]	4.22 [4.21–4.22]	4.41 [4.41–4.42]	4.62 [4.61–4.62]	5.04 [5.03–5.05]	4.45 [4.44–4.46]	4.82 [4.82–4.83]	5.04 [5.03–5.05]	5.27 [5.26–5.28]	5.76 [5.75–5.78]
41	3.88 [3.87–3.88]	4.22 [4.21–4.22]	4.41 [4.40–4.42]	4.61 [4.61–4.62]	5.04 [5.03–5.05]	4.44 [4.43–4.45]	4.81 [4.81–4.82]	5.03 [5.02–5.04]	5.27 [5.26–5.27]	5.76 [5.74–5.77]
42	3.87 [3.87–3.88]	4.22 [4.21–4.22]	4.41 [4.40–4.42]	4.61 [4.61–4.62]	5.04 [5.03–5.05]	4.43 [4.42–4.44]	4.81 [4.80–4.81]	5.02 [5.02–5.03]	5.26 [5.25–5.27]	5.75 [5.74–5.77]
43	3.87 [3.86–3.88]	4.22 [4.21–4.22]	4.41 [4.40–4.42]	4.61 [4.61–4.62]	5.04 [5.03–5.05]	4.42 [4.41–4.43]	4.80 [4.79–4.80]	5.01 [5.01–5.02]	5.25 [5.24–5.26]	5.75 [5.73–5.76]
44	3.87 [3.86–3.88]	4.22 [4.21–4.22]	4.41 [4.41–4.42]	4.62 [4.61–4.62]	5.04 [5.03–5.05]	4.41 [4.39–4.41]	4.79 [4.78–4.79]	5.01 [5.00–5.01]	5.24 [5.23–5.25]	5.74 [5.72–5.76]
45	3.87 [3.86–3.88]	4.22 [4.21–4.22]	4.41 [4.41–4.42]	4.62 [4.61–4.63]	5.04 [5.03–5.06]	4.39 [4.38–4.40]	4.78 [4.77–4.78]	5.00 [4.99–5.00]	5.23 [5.23–5.24]	5.73 [5.72–5.75]
46	3.87 [3.87–3.88]	4.22 [4.21–4.23]	4.42 [4.41–4.42]	4.62 [4.62–4.63]	5.05 [5.04–5.06]	4.38 [4.37–4.39]	4.77 [4.76–4.77]	4.99 [4.98–5.00]	5.22 [5.22–5.23]	5.73 [5.71–5.75]
47	3.88 [3.87–3.89]	4.22 [4.22–4.23]	4.42 [4.41–4.43]	4.63 [4.62–4.64]	5.06 [5.04–5.07]	4.37 [4.36–4.38]	4.76 [4.75–4.76]	4.98 [4.97–4.99]	5.22 [5.21–5.23]	5.72 [5.70–5.74]
48	3.88 [3.87–3.89]	4.23 [4.22–4.23]	4.43 [4.42–4.43]	4.63 [4.63–4.64]	5.06 [5.05–5.07]	4.36 [4.35–4.37]	4.75 [4.74–4.75]	4.97 [4.96–4.98]	5.21 [5.20–5.22]	5.72 [5.70–5.74]
49	3.88 [3.87–3.89]	4.23 [4.23–4.24]	4.43 [4.42–4.44]	4.64 [4.63–4.65]	5.07 [5.06–5.08]	4.35 [4.34–4.36]	4.73 [4.73–4.74]	4.96 [4.95–4.97]	5.20 [5.19–5.21]	5.71 [5.69–5.73]
50	3.89 [3.88–3.90]	4.24 [4.23–4.24]	4.44 [4.43–4.44]	4.65 [4.64–4.65]	5.08 [5.06–5.09]	4.34 [4.32–4.35]	4.72 [4.72–4.73]	4.95 [4.94–4.96]	5.19 [5.18–5.20]	5.70 [5.69–5.72]
51	3.89 [3.88–3.90]	4.24 [4.24–4.25]	4.44 [4.43–4.45]	4.65 [4.64–4.66]	5.08 [5.07–5.09]	4.33 [4.31–4.34]	4.71 [4.70–4.72]	4.94 [4.93–4.95]	5.18 [5.17–5.19]	5.70 [5.68–5.72]
52	3.90 [3.89–3.91]	4.25 [4.24–4.26]	4.45 [4.44–4.45]	4.66 [4.65–4.67]	5.09 [5.08–5.10]	4.31 [4.30–4.33]	4.70 [4.69–4.71]	4.93 [4.92–4.94]	5.17 [5.16–5.18]	5.69 [5.67–5.71]
53	3.90 [3.89–3.91]	4.25 [4.24–4.26]	4.45 [4.44–4.46]	4.66 [4.65–4.67]	5.10 [5.08–5.11]	4.30 [4.29–4.31]	4.69 [4.68–4.70]	4.92 [4.91–4.93]	5.16 [5.15–5.17]	5.68 [5.66–5.70]
54	3.90 [3.89–3.91]	4.25 [4.24–4.26]	4.45 [4.44–4.46]	4.66 [4.65–4.67]	5.10 [5.08–5.11]	4.29 [4.28–4.30]	4.68 [4.67–4.69]	4.91 [4.90–4.92]	5.15 [5.14–5.17]	5.67 [5.66–5.70]
55	3.90 [3.89–3.92]	4.25 [4.25–4.26]	4.45 [4.45–4.46]	4.67 [4.65–4.68]	5.10 [5.08–5.12]	4.28 [4.26–4.29]	4.67 [4.66–4.68]	4.90 [4.89–4.91]	5.14 [5.13–5.16]	5.67 [5.65–5.69]
56	3.90 [3.89–3.92]	4.25 [4.24–4.26]	4.45 [4.45–4.46]	4.67 [4.66–4.68]	5.10 [5.08–5.12]	4.26 [4.25–4.28]	4.66 [4.65–4.67]	4.89 [4.88–4.90]	5.13 [5.12–5.15]	5.66 [5.64–5.68]
57	3.90 [3.89–3.91]	4.25 [4.24–4.26]	4.45 [4.44–4.46]	4.66 [4.65–4.68]	5.10 [5.08–5.12]	4.25 [4.24–4.27]	4.65 [4.63–4.66]	4.88 [4.86–4.89]	5.12 [5.11–5.14]	5.65 [5.63–5.68]
58	3.90 [3.88–3.91]	4.25 [4.24–4.26]	4.45 [4.44–4.46]	4.66 [4.65–4.67]	5.10 [5.08–5.12]	4.24 [4.22–4.25]	4.63 [4.62–4.65]	4.86 [4.85–4.88]	5.11 [5.10–5.13]	5.64 [5.62–5.67]
59	3.89 [3.88–3.91]	4.25 [4.24–4.26]	4.45 [4.44–4.46]	4.66 [4.65–4.67]	5.10 [5.07–5.11]	4.23 [4.21–4.24]	4.62 [4.61–4.64]	4.85 [4.84–4.87]	5.10 [5.09–5.12]	5.64 [5.61–5.67]
60	3.89 [3.87–3.91]	4.24 [4.23–4.25]	4.44 [4.43–4.45]	4.65 [4.64–4.67]	5.09 [5.07–5.11]	4.21 [4.19–4.23]	4.61 [4.60–4.62]	4.84 [4.83–4.86]	5.09 [5.08–5.11]	5.63 [5.60–5.66]
61	3.88 [3.87–3.90]	4.24 [4.23–4.25]	4.44 [4.43–4.45]	4.65 [4.63–4.66]	5.09 [5.06–5.11]	4.20 [4.18–4.22]	4.60 [4.58–4.61]	4.83 [4.81–4.85]	5.08 [5.07–5.10]	5.62 [5.59–5.65]
62	3.88 [3.86–3.90]	4.23 [4.22–4.24]	4.43 [4.42–4.44]	4.64 [4.63–4.66]	5.08 [5.05–5.10]	4.19 [4.17–4.21]	4.59 [4.57–4.60]	4.82 [4.80–4.84]	5.07 [5.05–5.09]	5.61 [5.58–5.65]
63	3.87 [3.86–3.89]	4.22 [4.21–4.24]	4.42 [4.41–4.44]	4.64 [4.62–4.65]	5.07 [5.04–5.10]	4.17 [4.15–4.19]	4.58 [4.56–4.59]	4.81 [4.79–4.83]	5.06 [5.04–5.09]	5.61 [5.57–5.64]
64	3.87 [3.85–3.89]	4.22 [4.20–4.23]	4.42 [4.40–4.43]	4.63 [4.61–4.64]	5.07 [5.03–5.09]	4.16 [4.14–4.18]	4.56 [4.54–4.58]	4.80 [4.78–4.82]	5.05 [5.03–5.08]	5.60 [5.56–5.64]
65	3.86 [3.84–3.88]	4.21 [4.20–4.22]	4.41 [4.39–4.42]	4.62 [4.60–4.64]	5.06 [5.02–5.08]	4.15 [4.13–4.17]	4.55 [4.53–4.57]	4.79 [4.77–4.81]	5.04 [5.02–5.07]	5.59 [5.56–5.63]
66	3.85 [3.83–3.87]	4.20 [4.19–4.22]	4.40 [4.39–4.42]	4.61 [4.59–4.63]	5.05 [5.01–5.08]	4.14 [4.11–4.16]	4.54 [4.52–4.56]	4.78 [4.75–4.80]	5.03 [5.01–5.06]	5.58 [5.55–5.63]
67	3.84 [3.82–3.87]	4.20 [4.18–4.21]	4.39 [4.38–4.41]	4.61 [4.58–4.62]	5.04 [5.00–5.07]	4.12 [4.10–4.15]	4.53 [4.51–4.55]	4.77 [4.74–4.79]	5.02 [5.00–5.05]	5.58 [5.54–5.62]
68	3.84 [3.81–3.86]	4.19 [4.17–4.21]	4.39 [4.36–4.40]	4.60 [4.57–4.62]	5.03 [5.00–5.06]	4.11 [4.09–4.13]	4.52 [4.49–4.54]	4.76 [4.73–4.78]	5.01 [4.98–5.04]	5.57 [5.53–5.62]
69	3.83 [3.80–3.86]	4.18 [4.16–4.20]	4.38 [4.35–4.40]	4.59 [4.56–4.61]	5.03 [4.99–5.06]	4.10 [4.07–4.12]	4.51 [4.48–4.53]	4.75 [4.72–4.77]	5.01 [4.97–5.04]	5.56 [5.52–5.61]
70	3.82 [3.79–3.85]	4.17 [4.15–4.19]	4.37 [4.35–4.39]	4.58 [4.55–4.60]	5.02 [4.98–5.05]	4.09 [4.06–4.11]	4.50 [4.47–4.52]	4.74 [4.71–4.76]	5.00 [4.96–5.03]	5.56 [5.51–5.60]
71	3.81 [3.78–3.85]	4.16 [4.14–4.19]	4.36 [4.34–4.38]	4.57 [4.54–4.60]	5.01 [4.97–5.04]	4.08 [4.04–4.10]	4.48 [4.45–4.51]	4.73 [4.70–4.75]	4.99 [4.95–5.02]	5.55 [5.50–5.60]
72	3.81 [3.77–3.84]	4.16 [4.13–4.19]	4.36 [4.33–4.38]	4.57 [4.53–4.59]	5.00 [4.96–5.04]	4.06 [4.03–4.09]	4.47 [4.44–4.50]	4.72 [4.68–4.74]	4.98 [4.94–5.01]	5.54 [5.49–5.59]
73	3.80 [3.76–3.84]	4.15 [4.12–4.18]	4.35 [4.32–4.37]	4.56 [4.52–4.59]	4.99 [4.95–5.03]	4.05 [4.02–4.08]	4.46 [4.43–4.49]	4.71 [4.67–4.73]	4.97 [4.94–5.00]	5.54 [5.48–5.59]
74	3.79 [3.75–3.84]	4.14 [4.11–4.18]	4.34 [4.30–4.37]	4.55 [4.51–4.58]	4.99 [4.94–5.03]	4.04 [4.00–4.07]	4.45 [4.42–4.48]	4.69 [4.66–4.72]	4.96 [4.93–5.00]	5.53 [5.47–5.59]
75	3.79 [3.75–3.83]	4.14 [4.10–4.17]	4.33 [4.29–4.37]	4.54 [4.50–4.58]	4.98 [4.92–5.02]	4.03 [3.99–4.06]	4.44 [4.41–4.47]	4.68 [4.65–4.71]	4.95 [4.92–4.99]	5.52 [5.46–5.58]
76	3.78 [3.74–3.83]	4.13 [4.08–4.17]	4.33 [4.28–4.37]	4.54 [4.49–4.57]	4.97 [4.91–5.02]	4.01 [3.98–4.04]	4.43 [4.39–4.46]	4.67 [4.64–4.70]	4.94 [4.91–4.98]	5.52 [5.45–5.58]
77	3.77 [3.73–3.83]	4.12 [4.07–4.17]	4.32 [4.27–4.36]	4.53 [4.48–4.57]	4.96 [4.90–5.01]	4.00 [3.97–4.03]	4.42 [4.38–4.45]	4.66 [4.63–4.70]	4.93 [4.89–4.97]	5.51 [5.45–5.57]
78	3.77 [3.71–3.82]	4.11 [4.06–4.16]	4.31 [4.26–4.36]	4.52 [4.46–4.56]	4.96 [4.89–5.01]	3.99 [3.95–4.02]	4.41 [4.37–4.44]	4.65 [4.62–4.69]	4.92 [4.88–4.96]	5.50 [5.44–5.57]
79	3.76 [3.70–3.82]	4.11 [4.05–4.16]	4.30 [4.25–4.35]	4.51 [4.45–4.56]	4.95 [4.88–5.01]	3.98 [3.94–4.01]	4.40 [4.35–4.43]	4.64 [4.60–4.68]	4.91 [4.87–4.96]	5.50 [5.43–5.56]
80	3.75 [3.69–3.82]	4.10 [4.04–4.16]	4.30 [4.24–4.35]	4.51 [4.44–4.56]	4.94 [4.87–5.01]	3.97 [3.92–4.00]	4.39 [4.34–4.42]	4.63 [4.59–4.67]	4.90 [4.86–4.95]	5.49 [5.42–5.56]

**Table 3:** External validation of the next-generation reference interval for RBC across annual validation cohorts (2019–2023).

Year	Group	NRI	n	Total	Percent (%)
2019	Female	L	377	10712	3.52
2019	Female	N	10105	10712	94.33
2019	Female	U	230	10712	2.15
2019	Male	L	229	7184	3.19
2019	Male	N	6823	7184	94.97
2019	Male	U	132	7184	1.84
2020	Female	L	208	5512	3.77
2020	Female	N	5199	5512	94.32
2020	Female	U	105	5512	1.90
2020	Male	L	157	3722	4.22
2020	Male	N	3501	3722	94.06
2020	Male	U	64	3722	1.72
2021	Female	L	244	8883	2.75
2021	Female	N	8440	8883	95.01
2021	Female	U	199	8883	2.24
2021	Male	L	178	5688	3.13
2021	Male	N	5404	5688	95.01
2021	Male	U	106	5688	1.86
2022	Female	L	216	8259	2.62
2022	Female	N	7837	8259	94.89
2022	Female	U	206	8259	2.49
2022	Male	L	121	5167	2.34
2022	Male	N	4924	5167	95.30
2022	Male	U	122	5167	2.36
2023	Female	L	317	9283	3.41
2023	Female	N	8737	9283	94.12
2023	Female	U	229	9283	2.47
2023	Male	L	184	5554	3.31
2023	Male	N	5209	5554	93.79
2023	Male	U	161	5554	2.90

Note. Participants were classified according to the next-generation reference interval as L (below the lower reference limit), N (within the reference interval), or U (above the upper reference limit). Percentages were calculated as n/Total × 100 within each Year × Sex stratum. “Total” denotes the number of evaluable individuals in each stratum. Corresponding validation results for HGB, HCT, MCV, MCH, MCHC, WBC, and PLT are provided in Supplementary Tables S8–S14.

## Data Availability

The data are not publicly available due to privacy and institutional restrictions.

## Supplementary Materials

The following supporting information can be downloaded at: https://www.mdpi.com/article/10.3390/diagnostics16060944/s1, Supplementary Tables S1–S7: Age- and sex-specific percentile estimates (2.5th, 25th, 50th, 75th, and 97.5th) for each hematological parameter derived from the GAMLSS models. Supplementary Tables S8–S14: Annual validation results (2019–2023), including the number and proportion of individuals classified as below, within, or above the reference intervals. Supplementary Figures S1–S7: Age-continuous percentile curves for each hematological parameter. These supplementary materials provide detailed numerical results and additional validation evidence supporting the main findings of the study. Supplementary Figure S8: Age distribution of the training population, stratified by sex.

## Abbreviations

The following abbreviations are used in this manuscript:

RIs	Reference Intervals
NGRIs	Next-Generation Reference Intervals
GAMLSS	Generalized Additive Models for Location, Scale and Shape
BCCG	Box–Cox Cole and Green Distribution
RBC	Red Blood Cell Count
WBC	White Blood Cell Count
HGB	Hemoglobin
HCT	Hematocrit
MCV	Mean Corpuscular Volume
MCH	Mean Corpuscular Hemoglobin
MCHC	Mean Corpuscular Hemoglobin Concentration
PLT	Platelet Count
BMI	Body Mass Index
SBP	Systolic Blood Pressure
DBP	Diastolic Blood Pressure
IQR	Interquartile Range
