# Supplementary material for: Establishment and Temporal Validation of Next-Generation Reference Intervals for Routine Hematological Parameters Using Large-Scale Data
Source: Diagnostics (Basel). 2026 Mar 23;16(6):944. doi: 10.3390/diagnostics16060944 (PMC13025947; doi:10.3390/diagnostics16060944)
Supplement: Supplementary file 1 [file diagnostics-16-00944-s001.zip › Supplemental Table 1-7.pdf]

Supplemental Table 1. Model-predicted sex- and age-specific next-generation reference intervals for HGB.

Age	Female					Male				
	Q2.5	Q25	Q50	Q75	Q97.5	Q2.5	Q25	Q50	Q75	Q97.5
18	113 [111-115]	129 [128-130]	135 [134-136]	141 [140-141]	149 [148-150]	141 [140-143]	153 [152-154]	158 [158-160]	164 [163-165]	175 [174-176]
19	113 [112-115]	129 [128-129]	135 [134-135]	140 [140-141]	149 [148-150]	141 [140-142]	152 [152-153]	158 [157-159]	164 [163-165]	175 [174-176]
20	113 [112-114]	129 [128-129]	135 [134-135]	140 [140-141]	149 [148-150]	141 [140-142]	152 [151-153]	158 [157-159]	164 [163-164]	174 [173-175]
21	113 [112-114]	129 [128-129]	135 [134-135]	140 [140-141]	149 [148-149]	140 [140-141]	152 [151-153]	158 [157-158]	163 [163-164]	174 [173-175]
22	113 [112-114]	129 [128-129]	135 [134-135]	140 [140-140]	149 [148-149]	140 [139-141]	152 [151-152]	157 [157-158]	163 [163-164]	174 [173-174]
23	113 [112-114]	128 [128-129]	135 [134-135]	140 [140-140]	149 [148-149]	140 [139-140]	151 [151-152]	157 [157-157]	163 [162-163]	174 [173-174]
24	113 [113-114]	128 [128-129]	135 [134-135]	140 [140-140]	148 [148-149]	140 [139-140]	151 [151-151]	157 [157-157]	163 [162-163]	173 [173-174]
25	113 [113-114]	128 [128-129]	134 [134-135]	140 [140-140]	148 [148-149]	139 [139-140]	151 [150-151]	157 [156-157]	162 [162-163]	173 [173-174]
26	113 [113-114]	128 [128-128]	134 [134-135]	140 [140-140]	148 [148-149]	139 [139-140]	151 [150-151]	156 [156-157]	162 [162-162]	173 [173-173]
27	113 [113-113]	128 [128-128]	134 [134-134]	140 [140-140]	148 [148-149]	139 [138-139]	150 [150-151]	156 [156-156]	162 [162-162]	173 [172-173]
28	113 [112-113]	128 [128-128]	134 [134-134]	140 [139-140]	148 [148-148]	139 [138-139]	150 [150-150]	156 [156-156]	162 [162-162]	173 [172-173]
29	113	128	134	140	148	139	150	156	162	173

	[112-113]	[128-128]	[134-134]	[139-140]	[148-149]	[138-139]	[150-150]	[156-156]	[162-162]	[172-173]
30	112<br>[112-113]	128<br>[128-128]	134<br>[134-134]	140<br>[139-140]	148<br>[148-149]	138<br>[138-139]	150<br>[150-150]	156<br>[156-156]	162<br>[161-162]	172<br>[172-173]
31	112<br>[111-112]	128<br>[128-128]	134<br>[134-134]	139<br>[139-140]	148<br>[148-148]	138<br>[138-139]	150<br>[150-150]	156<br>[155-156]	161<br>[161-162]	172<br>[172-173]
32	111<br>[111-112]	127<br>[127-128]	134<br>[134-134]	139<br>[139-139]	148<br>[148-148]	138<br>[138-138]	150<br>[149-150]	156<br>[155-156]	161<br>[161-162]	172<br>[172-172]
33	111<br>[110-111]	127<br>[127-127]	134<br>[134-134]	139<br>[139-139]	148<br>[148-148]	138<br>[138-138]	150<br>[149-150]	155<br>[155-156]	161<br>[161-161]	172<br>[172-172]
34	110<br>[109-110]	127<br>[127-127]	133<br>[133-134]	139<br>[139-139]	148<br>[148-148]	138<br>[137-138]	149<br>[149-150]	155<br>[155-156]	161<br>[161-161]	172<br>[172-172]
35	109<br>[109-110]	127<br>[126-127]	133<br>[133-133]	139<br>[139-139]	148<br>[148-148]	138<br>[137-138]	149<br>[149-150]	155<br>[155-155]	161<br>[161-161]	172<br>[172-172]
36	108<br>[108-109]	126<br>[126-126]	133<br>[133-133]	139<br>[139-139]	148<br>[148-148]	138<br>[137-138]	149<br>[149-149]	155<br>[155-155]	161<br>[161-161]	172<br>[171-172]
37	107<br>[107-108]	126<br>[126-126]	133<br>[133-133]	139<br>[139-139]	148<br>[148-148]	137<br>[137-138]	149<br>[149-149]	155<br>[155-155]	161<br>[161-161]	172<br>[171-172]
38	106<br>[106-107]	125<br>[125-126]	133<br>[132-133]	139<br>[139-139]	148<br>[148-149]	137<br>[137-138]	149<br>[149-149]	155<br>[155-155]	161<br>[160-161]	172<br>[171-172]
39	106<br>[105-106]	125<br>[125-125]	132<br>[132-133]	139<br>[138-139]	148<br>[148-149]	137<br>[137-138]	149<br>[149-149]	155<br>[155-155]	161<br>[160-161]	172<br>[171-172]
40	105<br>[104-106]	125<br>[125-125]	132<br>[132-133]	139<br>[138-139]	149<br>[148-149]	137<br>[137-138]	149<br>[149-149]	155<br>[155-155]	161<br>[160-161]	171<br>[171-172]
41	104<br>[104-105]	125<br>[125-125]	132<br>[132-133]	139<br>[138-139]	149<br>[148-149]	137<br>[137-137]	149<br>[148-149]	155<br>[154-155]	161<br>[160-161]	171<br>[171-172]
42	104	125	132	139	149	137	149	155	160	171

	[103-105]	[124-125]	[132-133]	[139-139]	[149-149]	[137-137]	[148-149]	[154-155]	[160-161]	[171-172]
43	104	125	132	139	149	137	149	155	160	171
	[103-104]	[124-125]	[132-133]	[139-139]	[149-149]	[136-137]	[148-149]	[154-155]	[160-161]	[171-172]
44	104	125	133	139	149	137	149	154	160	171
	[103-104]	[125-125]	[132-133]	[139-139]	[149-149]	[136-137]	[148-149]	[154-155]	[160-161]	[171-172]
45	104	125	133	139	149	137	148	154	160	171
	[103-105]	[125-125]	[133-133]	[139-139]	[149-150]	[136-137]	[148-149]	[154-155]	[160-161]	[171-172]
46	105	125	133	139	150	137	148	154	160	171
	[104-105]	[125-126]	[133-133]	[139-140]	[149-150]	[136-137]	[148-149]	[154-155]	[160-161]	[171-172]
47	105	126	133	140	150	136	148	154	160	171
	[105-106]	[126-126]	[133-134]	[139-140]	[149-150]	[136-137]	[148-149]	[154-155]	[160-161]	[171-172]
48	107	126	134	140	150	136	148	154	160	171
	[106-107]	[126-127]	[134-134]	[140-140]	[149-150]	[136-137]	[148-149]	[154-155]	[160-161]	[171-172]
49	108	127	134	140	150	136	148	154	160	171
	[107-109]	[127-127]	[134-134]	[140-140]	[150-150]	[136-137]	[148-149]	[154-155]	[160-161]	[171-172]
50	109	127	134	140	150	136	148	154	160	171
	[108-110]	[127-128]	[134-135]	[140-141]	[150-150]	[135-137]	[148-148]	[154-154]	[160-161]	[171-172]
51	111	128	135	141	150	136	148	154	160	171
	[110-111]	[128-128]	[135-135]	[140-141]	[150-150]	[135-136]	[148-148]	[154-154]	[160-160]	[171-172]
52	112	129	135	141	150	136	148	154	160	171
	[111-113]	[128-129]	[135-135]	[140-141]	[149-150]	[135-136]	[147-148]	[154-154]	[160-160]	[171-172]
53	113	129	135	141	150	135	147	154	160	171
	[112-114]	[129-129]	[135-136]	[141-141]	[149-150]	[135-136]	[147-148]	[153-154]	[159-160]	[171-172]
54	114	129	136	141	150	135	147	154	160	171
	[113-115]	[129-130]	[135-136]	[141-141]	[149-150]	[134-136]	[147-148]	[153-154]	[159-160]	[171-172]
55	115	130	136	141	150	135	147	153	159	171

	[114-116]	[129-130]	[135-136]	[141-141]	[149-150]	[134-135]	[147-147]	[153-154]	[159-160]	[170-171]
56	115	130	136	141	150	134	147	153	159	171
	[115-116]	[130-130]	[136-136]	[141-141]	[149-150]	[134-135]	[146-147]	[153-153]	[159-160]	[170-171]
57	116	130	136	141	150	134	146	153	159	171
	[115-117]	[130-130]	[136-136]	[141-141]	[149-150]	[133-134]	[146-147]	[152-153]	[159-159]	[170-171]
58	116	130	136	141	149	134	146	153	159	170
	[115-117]	[130-131]	[136-136]	[141-141]	[149-150]	[133-134]	[146-146]	[152-153]	[158-159]	[170-171]
59	116	130	136	141	149	133	146	152	159	170
	[115-117]	[130-131]	[136-136]	[141-141]	[149-150]	[132-134]	[145-146]	[152-153]	[158-159]	[170-171]
60	116	130	136	141	149	133	145	152	158	170
	[115-117]	[130-131]	[136-136]	[141-142]	[149-150]	[132-133]	[145-146]	[151-152]	[158-159]	[169-171]
61	116	130	136	141	149	132	145	152	158	170
	[115-117]	[130-131]	[136-136]	[141-141]	[149-150]	[131-133]	[144-145]	[151-152]	[157-158]	[169-171]
62	116	130	136	141	149	132	145	151	158	170
	[115-117]	[130-130]	[135-136]	[140-141]	[149-150]	[131-133]	[144-145]	[151-152]	[157-158]	[169-170]
63	116	130	136	141	149	131	144	151	157	169
	[115-117]	[130-130]	[135-136]	[140-141]	[148-150]	[131-132]	[144-145]	[150-151]	[157-158]	[169-170]
64	116	130	136	141	149	131	144	151	157	169
	[115-117]	[129-130]	[135-136]	[140-141]	[148-150]	[130-132]	[143-144]	[150-151]	[156-158]	[168-170]
65	115	130	136	141	149	130	144	150	157	169
	[114-116]	[129-130]	[135-136]	[140-141]	[148-150]	[129-131]	[143-144]	[150-151]	[156-157]	[168-170]
66	115	130	135	141	149	130	143	150	156	169
	[114-116]	[129-130]	[135-136]	[140-141]	[148-150]	[129-131]	[142-144]	[149-150]	[156-157]	[168-170]
67	115	129	135	140	149	129	143	150	156	168
	[114-116]	[129-130]	[135-136]	[140-141]	[148-150]	[128-130]	[142-143]	[149-150]	[155-157]	[167-169]
68	115	129	135	140	149	129	142	149	156	168

	[114-116]	[129-130]	[135-136]	[140-141]	[148-149]	[128-130]	[142-143]	[148-150]	[155-157]	[167-169]
69	115	129	135	140	148	129	142	149	156	168
	[113-116]	[128-130]	[134-136]	[139-141]	[148-149]	[127-130]	[141-143]	[148-150]	[155-156]	[167-169]
70	114	129	135	140	148	128	142	148	155	168
	[113-116]	[128-130]	[134-135]	[139-140]	[147-149]	[127-129]	[141-142]	[148-149]	[154-156]	[166-169]
71	114	129	135	140	148	128	141	148	155	167
	[113-116]	[128-130]	[134-135]	[139-140]	[147-149]	[126-129]	[140-142]	[147-149]	[154-156]	[166-169]
72	114	129	135	140	148	127	141	148	155	167
	[112-116]	[128-129]	[134-135]	[139-140]	[147-149]	[125-128]	[140-142]	[147-149]	[153-156]	[166-169]
73	114	128	134	140	148	127	140	147	154	167
	[112-116]	[127-129]	[133-135]	[139-140]	[147-149]	[125-128]	[139-141]	[146-148]	[153-155]	[165-168]
74	114	128	134	139	148	126	140	147	154	167
	[111-116]	[127-129]	[133-135]	[138-140]	[147-149]	[124-127]	[139-141]	[146-148]	[152-155]	[165-168]
75	113	128	134	139	148	125	139	146	153	166
	[111-116]	[127-129]	[133-135]	[138-140]	[146-149]	[124-127]	[138-140]	[145-148]	[152-155]	[164-168]
76	113	128	134	139	148	125	139	146	153	166
	[110-116]	[127-129]	[133-135]	[138-140]	[146-149]	[123-127]	[138-140]	[145-147]	[152-155]	[164-168]
77	113	128	134	139	148	124	139	146	153	166
	[110-116]	[126-129]	[132-135]	[138-140]	[146-149]	[123-126]	[137-140]	[144-147]	[151-154]	[163-168]
78	113	128	134	139	148	124	138	145	152	165
	[109-116]	[126-129]	[132-135]	[138-140]	[146-149]	[122-126]	[137-139]	[144-147]	[151-154]	[163-168]
79	112	128	134	139	147	123	138	145	152	165
	[108-116]	[126-130]	[132-135]	[137-140]	[145-149]	[121-125]	[136-139]	[143-146]	[150-154]	[163-168]
80	112	127	133	139	147	123	137	144	151	165
	[108-117]	[125-130]	[131-135]	[137-140]	[145-149]	[121-125]	[135-139]	[143-146]	[150-154]	[162-168]

Supplemental Table 2. Model-predicted sex- and age-specific next-generation reference intervals for HCT.

Age	Female					Male				
	Q2.5	Q25	Q50	Q75	Q97.5	Q2.5	Q25	Q50	Q75	Q97.5
18	34.5 [34.2-34.9]	38.1 [37.9-38.2]	39.6 [39.5-39.8]	41.1 [40.9-41.2]	43.5 [43.3-43.7]	41.4 [41.1-41.7]	44.4 [44.2-44.7]	46.0 [45.8-46.2]	47.6 [47.3-47.8]	50.6 [50.3-50.9]
19	34.5 [34.2-34.8]	38.0 [37.9-38.2]	39.6 [39.5-39.7]	41.1 [40.9-41.2]	43.5 [43.3-43.7]	41.3 [41.1-41.6]	44.3 [44.1-44.6]	45.9 [45.7-46.1]	47.5 [47.3-47.7]	50.5 [50.3-50.8]
20	34.4 [34.2-34.7]	38.0 [37.9-38.1]	39.6 [39.5-39.7]	41.0 [40.9-41.2]	43.5 [43.4-43.7]	41.2 [41.0-41.5]	44.2 [44.1-44.4]	45.8 [45.7-46.0]	47.4 [47.2-47.6]	50.4 [50.2-50.7]
21	34.4 [34.2-34.6]	38.0 [37.9-38.1]	39.6 [39.5-39.7]	41.0 [40.9-41.1]	43.5 [43.4-43.6]	41.1 [41.0-41.4]	44.2 [44.0-44.3]	45.7 [45.6-45.9]	47.3 [47.2-47.5]	50.4 [50.2-50.6]
22	34.4 [34.2-34.5]	38.0 [37.9-38.1]	39.6 [39.5-39.6]	41.0 [40.9-41.1]	43.5 [43.4-43.6]	41.1 [40.9-41.3]	44.1 [44.0-44.2]	45.7 [45.5-45.8]	47.3 [47.1-47.4]	50.3 [50.1-50.5]
23	34.3 [34.2-34.5]	38.0 [37.9-38.0]	39.6 [39.5-39.6]	41.0 [41.0-41.1]	43.5 [43.4-43.6]	41.0 [40.8-41.1]	44.0 [43.9-44.1]	45.6 [45.5-45.7]	47.2 [47.1-47.3]	50.2 [50.1-50.4]
24	34.3 [34.2-34.4]	37.9 [37.9-38.0]	39.6 [39.5-39.6]	41.0 [41.0-41.1]	43.5 [43.4-43.6]	40.9 [40.8-41.0]	43.9 [43.8-44.0]	45.5 [45.4-45.6]	47.1 [47.0-47.2]	50.2 [50.0-50.3]
25	34.3 [34.2-34.4]	37.9 [37.9-38.0]	39.6 [39.5-39.6]	41.0 [41.0-41.1]	43.5 [43.5-43.6]	40.8 [40.7-41.0]	43.9 [43.8-43.9]	45.5 [45.4-45.5]	47.0 [47.0-47.1]	50.1 [50.0-50.2]
26	34.3 [34.2-34.3]	37.9 [37.9-38.0]	39.5 [39.5-39.6]	41.0 [41.0-41.1]	43.5 [43.5-43.6]	40.8 [40.7-40.9]	43.8 [43.7-43.9]	45.4 [45.3-45.5]	47.0 [46.9-47.0]	50.0 [49.9-50.1]
27	34.2 [34.1-34.3]	37.9 [37.9-38.0]	39.5 [39.5-39.6]	41.0 [41.0-41.1]	43.5 [43.5-43.6]	40.7 [40.6-40.8]	43.7 [43.7-43.8]	45.3 [45.3-45.4]	46.9 [46.9-47.0]	50.0 [49.9-50.1]
28	34.2 [34.1-34.3]	37.9 [37.8-37.9]	39.5 [39.5-39.6]	41.0 [41.0-41.1]	43.5 [43.5-43.6]	40.7 [40.5-40.7]	43.7 [43.6-43.7]	45.3 [45.2-45.3]	46.9 [46.8-46.9]	49.9 [49.8-50.0]
29	34.1 [34.0-34.2]	37.9 [37.8-37.9]	39.5 [39.5-39.6]	41.0 [41.0-41.1]	43.5 [43.5-43.6]	40.6 [40.5-40.7]	43.6 [43.6-43.7]	45.2 [45.2-45.3]	46.8 [46.8-46.9]	49.9 [49.8-50.0]
30	34.0 [33.9-34.2]	37.8 [37.8-37.9]	39.5 [39.4-39.5]	41.0 [41.0-41.0]	43.5 [43.5-43.6]	40.5 [40.4-40.6]	43.6 [43.5-43.6]	45.2 [45.1-45.2]	46.8 [46.7-46.8]	49.8 [49.8-49.9]
31	33.9 [33.8-34.1]	37.8 [37.7-37.8]	39.4 [39.4-39.5]	41.0 [40.9-41.0]	43.5 [43.5-43.6]	40.5 [40.4-40.6]	43.5 [43.5-43.6]	45.1 [45.1-45.2]	46.7 [46.7-46.8]	49.8 [49.7-49.9]
32	33.8 [33.7-33.9]	37.7 [37.6-37.7]	39.4 [39.4-39.4]	40.9 [40.9-41.0]	43.5 [43.5-43.6]	40.5 [40.4-40.6]	43.5 [43.5-43.6]	45.1 [45.1-45.2]	46.7 [46.7-46.8]	49.8 [49.7-49.9]
33	33.7 [33.5-33.8]	37.6 [37.6-37.7]	39.3 [39.3-39.4]	40.9 [40.9-40.9]	43.5 [43.5-43.6]	40.4 [40.3-40.5]	43.5 [43.4-43.5]	45.1 [45.0-45.1]	46.7 [46.6-46.7]	49.8 [49.7-49.8]
34	33.5 [33.4-33.6]	37.5 [37.5-37.6]	39.3 [39.2-39.3]	40.8 [40.8-40.9]	43.5 [43.4-43.5]	40.4 [40.3-40.5]	43.4 [43.4-43.5]	45.0 [45.0-45.1]	46.7 [46.6-46.7]	49.7 [49.6-49.8]
35	33.3 [33.2-33.4]	37.4 [37.4-37.5]	39.2 [39.2-39.2]	40.8 [40.8-40.8]	43.5 [43.4-43.5]	40.4 [40.3-40.4]	43.4 [43.3-43.5]	45.0 [45.0-45.1]	46.6 [46.6-46.7]	49.7 [49.6-49.8]
36	33.2 [33.1-33.3]	37.3 [37.3-37.4]	39.1 [39.1-39.2]	40.8 [40.7-40.8]	43.5 [43.4-43.5]	40.3 [40.3-40.4]	43.4 [43.3-43.4]	45.0 [44.9-45.1]	46.6 [46.5-46.7]	49.7 [49.6-49.8]

37	33.0 [32.9-33.1]	37.2 [37.2-37.3]	39.1 [39.0-39.1]	40.7 [40.7-40.8]	43.5 [43.4-43.5]	40.3 [40.2-40.4]	43.4 [43.3-43.4]	45.0 [44.9-45.0]	46.6 [46.5-46.7]	49.7 [49.6-49.8]
38	32.8 [32.7-32.9]	37.2 [37.1-37.2]	39.0 [39.0-39.1]	40.7 [40.6-40.7]	43.5 [43.4-43.6]	40.3 [40.2-40.4]	43.3 [43.3-43.4]	45.0 [44.9-45.0]	46.6 [46.5-46.6]	49.7 [49.6-49.8]
39	32.7 [32.5-32.8]	37.1 [37.0-37.2]	39.0 [38.9-39.0]	40.7 [40.6-40.7]	43.5 [43.4-43.6]	40.2 [40.2-40.3]	43.3 [43.2-43.4]	44.9 [44.9-45.0]	46.6 [46.5-46.6]	49.6 [49.5-49.7]
40	32.6 [32.4-32.7]	37.1 [37.0-37.1]	39.0 [38.9-39.0]	40.7 [40.6-40.7]	43.5 [43.4-43.6]	40.2 [40.1-40.3]	43.3 [43.2-43.4]	44.9 [44.9-45.0]	46.5 [46.5-46.6]	49.6 [49.5-49.7]
41	32.5 [32.3-32.6]	37.0 [37.0-37.1]	38.9 [38.9-39.0]	40.7 [40.6-40.7]	43.5 [43.4-43.6]	40.2 [40.1-40.3]	43.3 [43.2-43.3]	44.9 [44.8-45.0]	46.5 [46.5-46.6]	49.6 [49.5-49.7]
42	32.4 [32.2-32.5]	37.0 [36.9-37.1]	39.0 [38.9-39.0]	40.7 [40.6-40.8]	43.6 [43.5-43.7]	40.2 [40.1-40.3]	43.3 [43.2-43.3]	44.9 [44.8-45.0]	46.5 [46.5-46.6]	49.6 [49.5-49.7]
43	32.4 [32.2-32.5]	37.0 [36.9-37.1]	39.0 [38.9-39.0]	40.7 [40.7-40.8]	43.6 [43.5-43.7]	40.2 [40.1-40.3]	43.3 [43.2-43.3]	44.9 [44.8-45.0]	46.5 [46.5-46.6]	49.6 [49.5-49.8]
44	32.4 [32.2-32.5]	37.0 [37.0-37.1]	39.0 [39.0-39.1]	40.8 [40.7-40.8]	43.7 [43.6-43.7]	40.1 [40.0-40.2]	43.3 [43.2-43.3]	44.9 [44.8-45.0]	46.5 [46.5-46.6]	49.7 [49.6-49.8]
45	32.5 [32.3-32.6]	37.1 [37.0-37.2]	39.1 [39.0-39.1]	40.8 [40.8-40.9]	43.7 [43.6-43.8]	40.1 [40.0-40.2]	43.2 [43.2-43.3]	44.9 [44.8-45.0]	46.5 [46.5-46.6]	49.7 [49.6-49.8]
46	32.6 [32.4-32.7]	37.2 [37.1-37.3]	39.1 [39.1-39.2]	40.9 [40.8-41.0]	43.8 [43.7-43.9]	40.1 [40.0-40.2]	43.2 [43.2-43.3]	44.9 [44.8-45.0]	46.5 [46.5-46.6]	49.7 [49.6-49.8]
47	32.7 [32.6-32.9]	37.3 [37.2-37.4]	39.2 [39.2-39.3]	41.0 [40.9-41.0]	43.8 [43.7-43.9]	40.1 [40.0-40.2]	43.2 [43.2-43.3]	44.9 [44.8-45.0]	46.5 [46.5-46.6]	49.7 [49.6-49.8]
48	32.9 [32.8-33.1]	37.4 [37.3-37.5]	39.3 [39.3-39.4]	41.0 [41.0-41.1]	43.9 [43.8-44.0]	40.1 [40.0-40.2]	43.2 [43.1-43.3]	44.9 [44.8-45.0]	46.5 [46.5-46.6]	49.7 [49.6-49.8]
49	33.1 [33.0-33.3]	37.5 [37.5-37.6]	39.4 [39.4-39.5]	41.1 [41.1-41.2]	43.9 [43.8-44.0]	40.0 [39.9-40.2]	43.2 [43.1-43.3]	44.9 [44.8-45.0]	46.5 [46.5-46.6]	49.7 [49.6-49.9]
50	33.4 [33.2-33.6]	37.7 [37.6-37.8]	39.5 [39.5-39.6]	41.2 [41.1-41.2]	44.0 [43.9-44.0]	40.0 [39.9-40.2]	43.2 [43.1-43.3]	44.9 [44.8-45.0]	46.5 [46.5-46.6]	49.8 [49.6-49.9]
51	33.6 [33.5-33.8]	37.8 [37.7-37.9]	39.6 [39.6-39.7]	41.3 [41.2-41.3]	44.0 [43.9-44.1]	40.0 [39.8-40.1]	43.2 [43.1-43.3]	44.8 [44.7-44.9]	46.5 [46.4-46.6]	49.8 [49.6-49.9]
52	33.9 [33.7-34.0]	37.9 [37.9-38.0]	39.7 [39.6-39.8]	41.3 [41.2-41.4]	44.0 [43.9-44.1]	39.9 [39.8-40.1]	43.1 [43.0-43.3]	44.8 [44.7-44.9]	46.5 [46.4-46.6]	49.8 [49.6-49.9]
53	34.1 [33.9-34.2]	38.0 [38.0-38.1]	39.8 [39.7-39.8]	41.3 [41.3-41.4]	44.0 [43.9-44.1]	39.8 [39.7-40.0]	43.1 [43.0-43.2]	44.8 [44.7-44.9]	46.5 [46.4-46.6]	49.8 [49.6-49.9]
54	34.3 [34.1-34.4]	38.1 [38.1-38.2]	39.8 [39.8-39.9]	41.4 [41.3-41.4]	44.0 [43.9-44.1]	39.8 [39.6-39.9]	43.0 [42.9-43.1]	44.7 [44.6-44.8]	46.5 [46.4-46.5]	49.7 [49.6-49.9]
55	34.4 [34.3-34.6]	38.2 [38.1-38.3]	39.9 [39.8-39.9]	41.4 [41.3-41.5]	44.0 [43.9-44.1]	39.7 [39.5-39.8]	43.0 [42.9-43.1]	44.7 [44.6-44.8]	46.4 [46.3-46.5]	49.7 [49.6-49.8]
56	34.5 [34.4-34.7]	38.3 [38.2-38.3]	39.9 [39.8-40.0]	41.4 [41.3-41.5]	43.9 [43.8-44.0]	39.6 [39.4-39.7]	42.9 [42.8-43.0]	44.6 [44.5-44.7]	46.4 [46.3-46.5]	49.7 [49.6-49.8]
57	34.6 [34.5-34.8]	38.3 [38.2-38.4]	39.9 [39.8-40.0]	41.4 [41.3-41.5]	43.9 [43.8-44.0]	39.5 [39.4-39.7]	42.8 [42.7-42.9]	44.6 [44.5-44.7]	46.3 [46.2-46.4]	49.6 [49.5-49.8]
58	34.7 [34.5-34.9]	38.3 [38.2-38.4]	39.9 [39.8-40.0]	41.4 [41.3-41.5]	43.9 [43.7-44.0]	39.4 [39.3-39.6]	42.7 [42.6-42.8]	44.5 [44.4-44.6]	46.2 [46.1-46.4]	49.6 [49.5-49.8]
59	34.7 [34.6-34.9]	38.3 [38.2-38.4]	39.9 [39.8-40.0]	41.4 [41.3-41.5]	43.9 [43.7-44.0]	39.3 [39.1-39.5]	42.7 [42.5-42.8]	44.4 [44.3-44.5]	46.2 [46.1-46.3]	49.5 [49.4-49.7]
60	34.8 [34.6-35.0]	38.3 [38.2-38.4]	39.9 [39.8-40.0]	41.4 [41.3-41.5]	43.8 [43.7-44.0]	39.2 [39.0-39.4]	42.6 [42.4-42.7]	44.3 [44.2-44.5]	46.1 [46.0-46.2]	49.5 [49.4-49.7]

61	34.8 [34.6-35.0]	38.3 [38.2-38.4]	39.9 [39.8-40.0]	41.3 [41.2-41.4]	43.8 [43.6-43.9]	39.1 [38.9-39.3]	42.5 [42.3-42.6]	44.3 [44.1-44.4]	46.0 [45.9-46.2]	49.4 [49.3-49.6]
62	34.8 [34.6-35.0]	38.3 [38.2-38.4]	39.9 [39.8-40.0]	41.3 [41.2-41.4]	43.8 [43.6-43.9]	39.0 [38.8-39.2]	42.4 [42.2-42.5]	44.2 [44.0-44.3]	46.0 [45.8-46.1]	49.4 [49.2-49.6]
63	34.8 [34.6-35.0]	38.3 [38.2-38.4]	39.9 [39.7-39.9]	41.3 [41.2-41.4]	43.7 [43.6-43.9]	38.9 [38.7-39.1]	42.3 [42.1-42.4]	44.1 [43.9-44.2]	45.9 [45.7-46.1]	49.3 [49.1-49.6]
64	34.7 [34.5-34.9]	38.3 [38.1-38.4]	39.8 [39.7-39.9]	41.3 [41.1-41.4]	43.7 [43.5-43.9]	38.8 [38.5-39.0]	42.2 [42.0-42.3]	44.0 [43.8-44.2]	45.8 [45.6-46.0]	49.3 [49.1-49.5]
65	34.7 [34.5-34.9]	38.2 [38.1-38.4]	39.8 [39.7-39.9]	41.3 [41.1-41.4]	43.7 [43.5-43.9]	38.6 [38.4-38.9]	42.1 [41.9-42.3]	43.9 [43.7-44.1]	45.7 [45.5-45.9]	49.2 [49.0-49.4]
66	34.7 [34.4-34.9]	38.2 [38.1-38.4]	39.8 [39.7-39.9]	41.2 [41.1-41.4]	43.7 [43.5-43.9]	38.5 [38.3-38.8]	42.0 [41.8-42.2]	43.8 [43.6-44.0]	45.7 [45.5-45.9]	49.2 [48.9-49.4]
67	34.6 [34.4-34.9]	38.2 [38.0-38.3]	39.8 [39.6-39.9]	41.2 [41.1-41.3]	43.7 [43.5-43.8]	38.4 [38.2-38.7]	41.9 [41.7-42.1]	43.8 [43.5-43.9]	45.6 [45.4-45.8]	49.1 [48.9-49.4]
68	34.6 [34.3-34.8]	38.2 [38.0-38.3]	39.8 [39.6-39.9]	41.2 [41.1-41.3]	43.7 [43.4-43.8]	38.3 [38.0-38.6]	41.8 [41.6-42.0]	43.7 [43.5-43.9]	45.5 [45.3-45.7]	49.1 [48.8-49.3]
69	34.6 [34.3-34.8]	38.1 [37.9-38.3]	39.7 [39.5-39.9]	41.2 [41.0-41.3]	43.7 [43.4-43.9]	38.2 [37.9-38.5]	41.7 [41.5-41.9]	43.6 [43.4-43.8]	45.5 [45.2-45.7]	49.0 [48.7-49.3]
70	34.5 [34.2-34.8]	38.1 [37.9-38.3]	39.7 [39.5-39.9]	41.2 [41.0-41.3]	43.6 [43.4-43.9]	38.1 [37.8-38.4]	41.6 [41.4-41.8]	43.5 [43.3-43.7]	45.4 [45.1-45.6]	49.0 [48.6-49.3]
71	34.4 [34.1-34.8]	38.1 [37.8-38.3]	39.7 [39.4-39.9]	41.2 [40.9-41.3]	43.6 [43.4-43.9]	38.0 [37.7-38.3]	41.6 [41.3-41.8]	43.4 [43.2-43.7]	45.3 [45.0-45.5]	48.9 [48.5-49.2]
72	34.4 [33.9-34.8]	38.0 [37.8-38.3]	39.7 [39.4-39.9]	41.1 [40.9-41.3]	43.7 [43.4-43.9]	37.9 [37.5-38.2]	41.5 [41.2-41.7]	43.3 [43.1-43.6]	45.2 [44.9-45.5]	48.9 [48.4-49.2]
73	34.3 [33.8-34.8]	38.0 [37.7-38.3]	39.7 [39.4-39.9]	41.1 [40.9-41.4]	43.7 [43.3-44.0]	37.8 [37.4-38.1]	41.4 [41.1-41.6]	43.3 [43.0-43.5]	45.2 [44.8-45.4]	48.8 [48.4-49.1]
74	34.2 [33.7-34.8]	38.0 [37.6-38.3]	39.6 [39.3-39.9]	41.1 [40.8-41.4]	43.7 [43.3-44.0]	37.6 [37.2-38.0]	41.3 [41.0-41.6]	43.2 [42.9-43.5]	45.1 [44.7-45.4]	48.7 [48.3-49.1]
75	34.2 [33.5-34.8]	37.9 [37.6-38.3]	39.6 [39.3-39.9]	41.1 [40.8-41.4]	43.7 [43.3-44.0]	37.5 [37.1-37.9]	41.2 [40.8-41.5]	43.1 [42.8-43.4]	45.0 [44.6-45.3]	48.7 [48.2-49.1]
76	34.1 [33.4-34.8]	37.9 [37.5-38.4]	39.6 [39.2-39.9]	41.1 [40.7-41.4]	43.7 [43.2-44.1]	37.4 [37.0-37.8]	41.1 [40.7-41.4]	43.0 [42.7-43.3]	44.9 [44.5-45.3]	48.6 [48.1-49.1]
77	34.0 [33.2-34.9]	37.9 [37.4-38.4]	39.6 [39.1-39.9]	41.1 [40.7-41.4]	43.7 [43.2-44.1]	37.3 [36.8-37.7]	41.0 [40.6-41.4]	42.9 [42.5-43.3]	44.8 [44.4-45.2]	48.6 [48.0-49.0]
78	33.9 [33.0-34.9]	37.9 [37.3-38.4]	39.6 [39.1-40.0]	41.1 [40.6-41.5]	43.7 [43.2-44.2]	37.2 [36.7-37.6]	40.9 [40.5-41.3]	42.8 [42.4-43.2]	44.8 [44.3-45.1]	48.5 [47.9-49.0]
79	33.9 [32.8-34.9]	37.8 [37.3-38.4]	39.5 [39.0-40.0]	41.1 [40.6-41.5]	43.7 [43.2-44.2]	37.1 [36.6-37.5]	40.8 [40.4-41.2]	42.7 [42.3-43.1]	44.7 [44.2-45.1]	48.4 [47.8-49.0]
80	33.8 [32.6-34.9]	37.8 [37.2-38.4]	39.5 [38.9-40.0]	41.1 [40.5-41.5]	43.7 [43.1-44.3]	36.9 [36.4-37.4]	40.7 [40.2-41.1]	42.6 [42.2-43.1]	44.6 [44.1-45.0]	48.4 [47.7-48.9]

Supplemental Table 3. Model-predicted sex- and age-specific next-generation reference intervals for MCV.

Age	Female					Male				
	Q2.5	Q25	Q50	Q75	Q97.5	Q2.5	Q25	Q50	Q75	Q97.5
18	78.4 [77.5-79.3]	85.7 [85.4-86.0]	88.4 [88.1-88.6]	90.7 [90.4-90.9]	94.2 [93.9-94.6]	81.2 [80.8-81.7]	86.5 [86.1-86.9]	88.8 [88.5-89.2]	91.0 [90.6-91.4]	94.8 [94.3-95.1]
19	78.2 [77.4-79.0]	85.7 [85.4-86.0]	88.4 [88.2-88.6]	90.7 [90.5-90.9]	94.3 [94.1-94.6]	81.1 [80.7-81.5]	86.4 [86.1-86.7]	88.8 [88.5-89.1]	91.0 [90.6-91.3]	94.7 [94.3-95.0]
20	78.1 [77.4-78.8]	85.7 [85.5-85.9]	88.5 [88.3-88.6]	90.8 [90.6-91.0]	94.4 [94.2-94.7]	80.9 [80.6-81.4]	86.3 [86.1-86.6]	88.7 [88.4-88.9]	90.9 [90.6-91.2]	94.7 [94.3-94.9]
21	77.9 [77.3-78.7]	85.7 [85.5-85.9]	88.5 [88.4-88.6]	90.9 [90.7-91.0]	94.5 [94.3-94.7]	80.8 [80.5-81.3]	86.2 [86.0-86.4]	88.6 [88.4-88.8]	90.9 [90.6-91.0]	94.6 [94.3-94.9]
22	77.8 [77.3-78.5]	85.7 [85.5-85.9]	88.5 [88.4-88.6]	90.9 [90.8-91.1]	94.6 [94.5-94.8]	80.7 [80.4-81.1]	86.1 [86.0-86.3]	88.6 [88.4-88.7]	90.8 [90.6-91.0]	94.6 [94.3-94.8]
23	77.7 [77.3-78.2]	85.7 [85.6-85.8]	88.6 [88.5-88.7]	91.0 [90.9-91.1]	94.7 [94.6-94.8]	80.6 [80.3-81.0]	86.1 [85.9-86.2]	88.5 [88.4-88.6]	90.8 [90.6-90.9]	94.6 [94.4-94.8]
24	77.6 [77.2-78.0]	85.7 [85.6-85.8]	88.6 [88.5-88.7]	91.0 [90.9-91.1]	94.8 [94.7-94.9]	80.5 [80.2-80.9]	86.0 [85.9-86.1]	88.5 [88.3-88.6]	90.7 [90.6-90.8]	94.6 [94.4-94.7]
25	77.5 [77.1-77.8]	85.7 [85.6-85.8]	88.6 [88.6-88.7]	91.1 [91.0-91.1]	94.9 [94.7-95.0]	80.4 [80.2-80.8]	85.9 [85.8-86.1]	88.4 [88.3-88.5]	90.7 [90.6-90.8]	94.5 [94.4-94.7]
26	77.4 [77.0-77.6]	85.7 [85.6-85.8]	88.6 [88.6-88.7]	91.1 [91.0-91.2]	94.9 [94.8-95.0]	80.3 [80.1-80.6]	85.9 [85.8-86.0]	88.4 [88.3-88.5]	90.7 [90.6-90.8]	94.5 [94.4-94.7]
27	77.3 [77.0-77.6]	85.7 [85.6-85.8]	88.7 [88.6-88.7]	91.1 [91.1-91.2]	94.9 [94.9-95.0]	80.3 [80.0-80.5]	85.9 [85.8-86.0]	88.4 [88.3-88.4]	90.7 [90.6-90.8]	94.6 [94.4-94.7]
28	77.2 [76.9-77.5]	85.7 [85.6-85.7]	88.7 [88.6-88.7]	91.1 [91.1-91.2]	95.0 [94.9-95.1]	80.2 [79.9-80.4]	85.8 [85.8-85.9]	88.4 [88.3-88.4]	90.7 [90.6-90.8]	94.6 [94.4-94.7]
29	77.1 [76.8-77.3]	85.6 [85.6-85.7]	88.7 [88.6-88.7]	91.1 [91.1-91.2]	95.0 [94.9-95.1]	80.1 [79.9-80.4]	85.8 [85.7-85.9]	88.4 [88.3-88.4]	90.7 [90.6-90.8]	94.6 [94.5-94.8]

30	77.0 [76.8-77.2]	85.6 [85.5-85.7]	88.6 [88.6-88.7]	91.2 [91.1-91.2]	95.0 [94.9-95.1]	80.1 [79.9-80.3]	85.8 [85.7-85.9]	88.4 [88.3-88.4]	90.7 [90.6-90.8]	94.6 [94.5-94.8]
31	76.9 [76.6-77.1]	85.6 [85.5-85.7]	88.6 [88.6-88.7]	91.2 [91.1-91.2]	95.0 [95.0-95.1]	80.1 [79.8-80.3]	85.8 [85.7-85.9]	88.4 [88.3-88.5]	90.7 [90.7-90.8]	94.7 [94.6-94.9]
32	76.7 [76.4-77.0]	85.5 [85.5-85.6]	88.6 [88.6-88.7]	91.2 [91.1-91.2]	95.1 [95.0-95.2]	80.0 [79.8-80.2]	85.9 [85.8-85.9]	88.4 [88.4-88.5]	90.8 [90.7-90.9]	94.8 [94.7-95.0]
33	76.5 [76.3-76.8]	85.5 [85.4-85.6]	88.6 [88.6-88.7]	91.2 [91.1-91.2]	95.1 [95.0-95.2]	80.0 [79.8-80.2]	85.9 [85.8-86.0]	88.5 [88.4-88.6]	90.9 [90.8-91.0]	94.9 [94.8-95.0]
34	76.3 [76.0-76.5]	85.4 [85.4-85.5]	88.6 [88.5-88.7]	91.2 [91.1-91.3]	95.2 [95.1-95.3]	80.0 [79.8-80.3]	86.0 [85.9-86.0]	88.6 [88.5-88.7]	91.0 [90.9-91.1]	95.0 [94.9-95.1]
35	76.0 [75.7-76.2]	85.4 [85.3-85.5]	88.6 [88.5-88.7]	91.2 [91.2-91.3]	95.3 [95.2-95.3]	80.1 [79.8-80.3]	86.0 [85.9-86.1]	88.7 [88.6-88.8]	91.1 [91.0-91.2]	95.2 [95.0-95.3]
36	75.6 [75.3-75.9]	85.3 [85.2-85.4]	88.6 [88.5-88.7]	91.3 [91.2-91.3]	95.4 [95.3-95.4]	80.1 [79.9-80.3]	86.1 [86.0-86.2]	88.8 [88.7-88.9]	91.2 [91.1-91.3]	95.3 [95.2-95.4]
37	75.3 [75.0-75.6]	85.2 [85.1-85.3]	88.6 [88.5-88.7]	91.3 [91.3-91.4]	95.5 [95.4-95.5]	80.2 [80.0-80.4]	86.2 [86.2-86.3]	88.9 [88.8-89.0]	91.4 [91.3-91.4]	95.5 [95.4-95.6]
38	75.0 [74.6-75.3]	85.2 [85.1-85.3]	88.6 [88.5-88.7]	91.4 [91.3-91.4]	95.6 [95.5-95.6]	80.2 [80.0-80.5]	86.4 [86.3-86.5]	89.1 [89.0-89.2]	91.5 [91.4-91.6]	95.7 [95.6-95.8]
39	74.6 [74.3-75.0]	85.1 [85.0-85.3]	88.6 [88.5-88.7]	91.4 [91.4-91.5]	95.7 [95.6-95.8]	80.3 [80.1-80.6]	86.5 [86.4-86.6]	89.2 [89.1-89.3]	91.7 [91.6-91.8]	95.9 [95.7-96.0]
40	74.3 [74.0-74.8]	85.1 [85.0-85.2]	88.6 [88.6-88.7]	91.5 [91.4-91.6]	95.8 [95.7-95.9]	80.4 [80.1-80.6]	86.6 [86.5-86.7]	89.4 [89.3-89.4]	91.8 [91.8-91.9]	96.0 [95.9-96.2]
41	74.1 [73.7-74.5]	85.1 [85.0-85.2]	88.7 [88.6-88.7]	91.6 [91.5-91.6]	95.9 [95.8-96.0]	80.4 [80.2-80.7]	86.7 [86.6-86.8]	89.5 [89.4-89.6]	92.0 [91.9-92.1]	96.2 [96.1-96.4]
42	74.0 [73.6-74.4]	85.1 [85.0-85.2]	88.7 [88.6-88.8]	91.6 [91.6-91.7]	96.0 [95.9-96.1]	80.5 [80.3-80.8]	86.9 [86.8-87.0]	89.7 [89.6-89.8]	92.2 [92.1-92.3]	96.4 [96.3-96.6]

43	73.9 [73.6-74.4]	85.1 [85.0-85.2]	88.8 [88.7-88.8]	91.7 [91.6-91.7]	96.0 [95.9-96.1]	80.6 [80.3-80.9]	87.0 [86.9-87.1]	89.8 [89.7-89.9]	92.4 [92.2-92.5]	96.6 [96.5-96.8]
44	74.0 [73.7-74.5]	85.2 [85.1-85.3]	88.8 [88.7-88.9]	91.7 [91.6-91.8]	96.1 [96.0-96.2]	80.7 [80.4-81.0]	87.1 [87.0-87.2]	90.0 [89.8-90.1]	92.5 [92.4-92.6]	96.8 [96.7-97.0]
45	74.3 [73.9-74.7]	85.3 [85.2-85.4]	88.9 [88.8-88.9]	91.8 [91.7-91.8]	96.1 [96.0-96.2]	80.7 [80.4-81.0]	87.3 [87.1-87.4]	90.1 [90.0-90.2]	92.7 [92.6-92.8]	97.0 [96.9-97.2]
46	74.6 [74.2-75.1]	85.4 [85.3-85.5]	88.9 [88.9-89.0]	91.8 [91.7-91.9]	96.1 [96.0-96.2]	80.8 [80.5-81.1]	87.4 [87.2-87.5]	90.3 [90.1-90.4]	92.9 [92.7-93.0]	97.2 [97.1-97.4]
47	75.0 [74.6-75.5]	85.5 [85.4-85.6]	89.0 [88.9-89.1]	91.8 [91.7-91.9]	96.0 [95.9-96.1]	80.9 [80.5-81.2]	87.5 [87.4-87.6]	90.4 [90.3-90.5]	93.0 [92.9-93.1]	97.4 [97.2-97.6]
48	75.5 [75.1-75.9]	85.6 [85.5-85.7]	89.0 [89.0-89.1]	91.8 [91.7-91.9]	96.0 [95.9-96.1]	80.9 [80.6-81.3]	87.6 [87.5-87.8]	90.6 [90.4-90.7]	93.2 [93.0-93.3]	97.6 [97.4-97.8]
49	76.0 [75.6-76.5]	85.8 [85.7-85.9]	89.1 [89.0-89.2]	91.8 [91.7-91.9]	95.9 [95.8-96.0]	81.0 [80.7-81.4]	87.8 [87.6-87.9]	90.7 [90.6-90.8]	93.3 [93.2-93.5]	97.8 [97.6-98.0]
50	76.6 [76.2-77.0]	86.0 [85.8-86.1]	89.2 [89.1-89.3]	91.8 [91.7-91.9]	95.8 [95.7-96.0]	81.1 [80.7-81.4]	87.9 [87.7-88.0]	90.8 [90.7-91.0]	93.5 [93.3-93.6]	98.0 [97.8-98.2]
51	77.2 [76.8-77.6]	86.1 [86.0-86.3]	89.2 [89.2-89.3]	91.8 [91.7-91.9]	95.8 [95.6-95.9]	81.1 [80.8-81.5]	88.0 [87.8-88.1]	91.0 [90.8-91.1]	93.7 [93.5-93.8]	98.2 [98.0-98.4]
52	77.8 [77.3-78.2]	86.3 [86.2-86.4]	89.3 [89.2-89.4]	91.8 [91.7-91.9]	95.7 [95.5-95.8]	81.2 [80.8-81.6]	88.1 [87.9-88.3]	91.1 [91.0-91.2]	93.8 [93.7-94.0]	98.3 [98.1-98.5]
53	78.3 [77.8-78.8]	86.4 [86.3-86.6]	89.4 [89.3-89.5]	91.8 [91.7-91.9]	95.6 [95.5-95.8]	81.2 [80.8-81.6]	88.2 [88.0-88.4]	91.2 [91.1-91.4]	93.9 [93.8-94.1]	98.5 [98.3-98.7]
54	78.7 [78.3-79.2]	86.6 [86.5-86.7]	89.5 [89.4-89.5]	91.8 [91.7-91.9]	95.6 [95.4-95.7]	81.3 [80.8-81.7]	88.3 [88.1-88.5]	91.3 [91.2-91.5]	94.1 [93.9-94.2]	98.6 [98.4-98.9]
55	79.2 [78.8-79.6]	86.7 [86.6-86.9]	89.5 [89.4-89.6]	91.9 [91.8-92.0]	95.5 [95.4-95.7]	81.3 [80.8-81.7]	88.4 [88.2-88.6]	91.4 [91.3-91.6]	94.2 [94.0-94.4]	98.8 [98.6-99.0]

56	79.5 [79.1-79.9]	86.9 [86.8-87.0]	89.6 [89.5-89.7]	91.9 [91.8-92.0]	95.5 [95.4-95.7]	81.3 [80.8-81.8]	88.4 [88.2-88.6]	91.5 [91.4-91.7]	94.3 [94.1-94.5]	98.9 [98.7-99.2]
57	79.8 [79.5-80.3]	87.0 [86.9-87.1]	89.7 [89.6-89.8]	92.0 [91.8-92.1]	95.5 [95.3-95.7]	81.3 [80.8-81.8]	88.5 [88.3-88.7]	91.6 [91.5-91.8]	94.4 [94.2-94.6]	99.1 [98.8-99.3]
58	80.1 [79.7-80.6]	87.1 [87.0-87.3]	89.8 [89.7-89.9]	92.0 [91.9-92.1]	95.5 [95.4-95.7]	81.3 [80.8-81.8]	88.6 [88.3-88.8]	91.7 [91.5-91.9]	94.5 [94.3-94.7]	99.2 [98.9-99.4]
59	80.3 [79.9-80.8]	87.2 [87.1-87.4]	89.9 [89.8-90.0]	92.1 [92.0-92.2]	95.6 [95.4-95.7]	81.3 [80.8-81.9]	88.6 [88.4-88.8]	91.8 [91.6-91.9]	94.6 [94.4-94.8]	99.3 [99.0-99.5]
60	80.5 [80.1-81.0]	87.4 [87.2-87.5]	89.9 [89.8-90.1]	92.1 [92.0-92.3]	95.6 [95.4-95.8]	81.3 [80.7-81.9]	88.7 [88.4-88.9]	91.8 [91.7-92.0]	94.7 [94.5-94.9]	99.4 [99.1-99.7]
61	80.7 [80.3-81.2]	87.5 [87.3-87.6]	90.0 [89.9-90.2]	92.2 [92.1-92.4]	95.7 [95.5-95.9]	81.3 [80.7-81.9]	88.7 [88.4-88.9]	91.9 [91.7-92.1]	94.7 [94.5-94.9]	99.5 [99.2-99.8]
62	80.9 [80.4-81.4]	87.6 [87.4-87.7]	90.1 [90.0-90.3]	92.3 [92.2-92.5]	95.8 [95.5-96.0]	81.2 [80.6-81.9]	88.7 [88.4-89.0]	91.9 [91.7-92.1]	94.8 [94.6-95.0]	99.6 [99.3-99.9]
63	81.0 [80.5-81.5]	87.7 [87.5-87.8]	90.2 [90.1-90.4]	92.4 [92.3-92.6]	95.8 [95.6-96.1]	81.2 [80.5-81.9]	88.8 [88.5-89.0]	92.0 [91.8-92.2]	94.9 [94.6-95.1]	99.6 [99.4-100.0]
64	81.1 [80.6-81.6]	87.8 [87.6-88.0]	90.3 [90.2-90.5]	92.5 [92.3-92.7]	95.9 [95.7-96.2]	81.2 [80.5-81.9]	88.8 [88.5-89.0]	92.0 [91.8-92.2]	94.9 [94.7-95.2]	99.7 [99.4-100.0]
65	81.2 [80.6-81.8]	87.9 [87.7-88.1]	90.4 [90.3-90.6]	92.6 [92.4-92.8]	96.0 [95.8-96.3]	81.2 [80.4-81.9]	88.8 [88.5-89.1]	92.0 [91.8-92.3]	95.0 [94.7-95.2]	99.8 [99.5-100.1]
66	81.3 [80.7-81.9]	88.0 [87.8-88.2]	90.5 [90.4-90.7]	92.7 [92.5-92.9]	96.1 [95.9-96.4]	81.1 [80.3-81.9]	88.8 [88.5-89.1]	92.1 [91.9-92.3]	95.0 [94.8-95.2]	99.9 [99.5-100.2]
67	81.3 [80.8-81.9]	88.0 [87.9-88.3]	90.6 [90.5-90.8]	92.8 [92.6-93.0]	96.3 [96.0-96.5]	81.1 [80.2-82.0]	88.8 [88.5-89.2]	92.1 [91.9-92.4]	95.0 [94.8-95.3]	99.9 [99.6-100.3]
68	81.4 [80.8-82.0]	88.1 [87.9-88.4]	90.7 [90.5-90.9]	92.9 [92.7-93.1]	96.4 [96.1-96.7]	81.0 [80.2-81.9]	88.8 [88.5-89.2]	92.1 [91.9-92.4]	95.1 [94.8-95.4]	100.0 [99.6-100.3]

69	81.5<br>[80.8-82.2]	88.2<br>[88.0-88.5]	90.8<br>[90.6-91.1]	93.0<br>[92.8-93.3]	96.5<br>[96.2-96.8]	81.0<br>[80.1-81.9]	88.8<br>[88.5-89.2]	92.1<br>[91.9-92.4]	95.1<br>[94.8-95.4]	100.0<br>[99.7-100.4]
70	81.5<br>[80.8-82.3]	88.3<br>[88.1-88.6]	90.9<br>[90.7-91.2]	93.1<br>[92.9-93.4]	96.6<br>[96.3-97.0]	80.9<br>[80.0-81.9]	88.8<br>[88.4-89.2]	92.2<br>[91.8-92.5]	95.1<br>[94.8-95.4]	100.1<br>[99.7-100.5]
71	81.6<br>[80.8-82.4]	88.4<br>[88.1-88.7]	91.0<br>[90.8-91.3]	93.2<br>[93.0-93.5]	96.7<br>[96.3-97.1]	80.8<br>[79.9-81.9]	88.8<br>[88.4-89.3]	92.2<br>[91.8-92.5]	95.2<br>[94.8-95.5]	100.1<br>[99.7-100.6]
72	81.6<br>[80.7-82.5]	88.5<br>[88.2-88.8]	91.1<br>[90.9-91.4]	93.3<br>[93.0-93.7]	96.8<br>[96.4-97.2]	80.8<br>[79.8-81.9]	88.8<br>[88.3-89.3]	92.2<br>[91.8-92.5]	95.2<br>[94.8-95.5]	100.2<br>[99.8-100.6]
73	81.7<br>[80.6-82.7]	88.6<br>[88.2-89.0]	91.2<br>[90.9-91.5]	93.4<br>[93.1-93.8]	97.0<br>[96.5-97.4]	80.7<br>[79.7-81.9]	88.8<br>[88.2-89.3]	92.2<br>[91.7-92.6]	95.2<br>[94.8-95.5]	100.2<br>[99.8-100.7]
74	81.7<br>[80.6-82.8]	88.7<br>[88.3-89.1]	91.3<br>[91.0-91.7]	93.6<br>[93.2-93.9]	97.1<br>[96.6-97.5]	80.7<br>[79.6-82.0]	88.8<br>[88.2-89.4]	92.2<br>[91.7-92.6]	95.3<br>[94.8-95.6]	100.3<br>[99.8-100.8]
75	81.7<br>[80.5-82.9]	88.8<br>[88.3-89.3]	91.4<br>[91.1-91.8]	93.7<br>[93.2-94.1]	97.2<br>[96.7-97.7]	80.6<br>[79.5-82.0]	88.8<br>[88.1-89.4]	92.2<br>[91.6-92.6]	95.3<br>[94.8-95.6]	100.3<br>[99.8-100.9]
76	81.7<br>[80.3-83.1]	88.8<br>[88.4-89.4]	91.5<br>[91.1-91.9]	93.8<br>[93.3-94.2]	97.4<br>[96.8-97.9]	80.5<br>[79.3-82.0]	88.8<br>[88.0-89.4]	92.2<br>[91.6-92.7]	95.3<br>[94.8-95.7]	100.4<br>[99.8-100.9]
77	81.7<br>[80.2-83.2]	88.9<br>[88.4-89.5]	91.6<br>[91.2-92.0]	93.9<br>[93.4-94.4]	97.5<br>[96.8-98.0]	80.5<br>[79.2-82.1]	88.8<br>[87.9-89.5]	92.2<br>[91.5-92.7]	95.3<br>[94.7-95.8]	100.4<br>[99.7-101.0]
78	81.7<br>[80.0-83.4]	89.0<br>[88.5-89.6]	91.7<br>[91.3-92.2]	94.0<br>[93.5-94.5]	97.6<br>[96.9-98.2]	80.4<br>[79.0-82.1]	88.8<br>[87.8-89.5]	92.3<br>[91.5-92.8]	95.4<br>[94.7-95.9]	100.5<br>[99.7-101.1]
79	81.7<br>[79.9-83.6]	89.1<br>[88.5-89.7]	91.8<br>[91.3-92.3]	94.1<br>[93.6-94.7]	97.8<br>[97.0-98.4]	80.3<br>[78.9-82.1]	88.7<br>[87.8-89.5]	92.3<br>[91.5-92.9]	95.4<br>[94.6-95.9]	100.5<br>[99.7-101.1]
80	81.7<br>[79.6-83.7]	89.2<br>[88.6-89.8]	91.9<br>[91.4-92.4]	94.2<br>[93.7-94.8]	97.9<br>[97.1-98.6]	80.2<br>[78.7-82.1]	88.7<br>[87.7-89.6]	92.3<br>[91.5-92.9]	95.4<br>[94.6-96.0]	100.6<br>[99.7-101.2]

Supplemental Table 4. Model-predicted sex- and age-specific next-generation reference intervals for MCH.

Age	Female					Male				
	Q2.5	Q25	Q50	Q75	Q97.5	Q2.5	Q25	Q50	Q75	Q97.5
18	25.7 [25.3-26.1]	29.0 [28.9-29.1]	30.1 [30.0-30.2]	31.0 [30.9-31.1]	32.4 [32.2-32.5]	27.7 [27.5-27.9]	29.7 [29.6-29.9]	30.6 [30.5-30.7]	31.4 [31.3-31.5]	32.7 [32.6-32.9]
19	25.7 [25.3-26.1]	29.0 [28.9-29.1]	30.1 [30.1-30.2]	31.0 [30.9-31.1]	32.4 [32.3-32.5]	27.6 [27.5-27.8]	29.7 [29.6-29.8]	30.6 [30.5-30.7]	31.4 [31.3-31.5]	32.7 [32.6-32.8]
20	25.7 [25.4-26.0]	29.0 [28.9-29.1]	30.1 [30.1-30.2]	31.0 [31.0-31.1]	32.4 [32.3-32.5]	27.6 [27.4-27.8]	29.7 [29.6-29.8]	30.6 [30.5-30.7]	31.4 [31.3-31.5]	32.7 [32.6-32.8]
21	25.7 [25.4-25.9]	29.0 [29.0-29.1]	30.1 [30.1-30.2]	31.0 [31.0-31.1]	32.4 [32.3-32.5]	27.5 [27.4-27.7]	29.7 [29.6-29.7]	30.6 [30.5-30.6]	31.4 [31.3-31.4]	32.7 [32.6-32.8]
22	25.6 [25.4-25.8]	29.0 [29.0-29.1]	30.1 [30.1-30.2]	31.0 [31.0-31.1]	32.4 [32.4-32.5]	27.5 [27.4-27.7]	29.6 [29.6-29.7]	30.5 [30.5-30.6]	31.3 [31.3-31.4]	32.7 [32.6-32.8]
23	25.6 [25.4-25.8]	29.0 [29.0-29.1]	30.1 [30.1-30.2]	31.1 [31.0-31.1]	32.4 [32.4-32.5]	27.5 [27.3-27.6]	29.6 [29.5-29.7]	30.5 [30.5-30.6]	31.3 [31.3-31.4]	32.7 [32.6-32.7]
24	25.6 [25.4-25.7]	29.0 [29.0-29.1]	30.1 [30.1-30.2]	31.1 [31.0-31.1]	32.4 [32.4-32.5]	27.4 [27.3-27.6]	29.6 [29.5-29.6]	30.5 [30.5-30.5]	31.3 [31.3-31.4]	32.6 [32.6-32.7]
25	25.6 [25.4-25.7]	29.0 [29.0-29.1]	30.1 [30.1-30.2]	31.1 [31.0-31.1]	32.4 [32.4-32.5]	27.4 [27.3-27.5]	29.6 [29.5-29.6]	30.5 [30.4-30.5]	31.3 [31.3-31.3]	32.6 [32.6-32.7]
26	25.5 [25.4-25.7]	29.0 [29.0-29.0]	30.1 [30.1-30.2]	31.1 [31.0-31.1]	32.5 [32.4-32.5]	27.4 [27.3-27.5]	29.6 [29.5-29.6]	30.5 [30.4-30.5]	31.3 [31.3-31.3]	32.6 [32.6-32.7]
27	25.5 [25.3-25.6]	29.0 [29.0-29.0]	30.1 [30.1-30.2]	31.1 [31.0-31.1]	32.5 [32.4-32.5]	27.3 [27.3-27.5]	29.6 [29.5-29.6]	30.5 [30.5-30.5]	31.3 [31.3-31.3]	32.7 [32.6-32.7]
28	25.4 [25.3-25.6]	29.0 [29.0-29.0]	30.1 [30.1-30.2]	31.1 [31.0-31.1]	32.5 [32.4-32.5]	27.3 [27.2-27.4]	29.5 [29.5-29.6]	30.5 [30.5-30.5]	31.3 [31.3-31.3]	32.7 [32.6-32.7]
29	25.4 [25.3-25.5]	29.0 [28.9-29.0]	30.1 [30.1-30.2]	31.1 [31.0-31.1]	32.5 [32.4-32.5]	27.3 [27.2-27.4]	29.5 [29.5-29.6]	30.5 [30.5-30.5]	31.3 [31.3-31.3]	32.7 [32.6-32.7]
30	25.3 [25.2-25.4]	29.0 [28.9-29.0]	30.1 [30.1-30.2]	31.1 [31.1-31.1]	32.5 [32.5-32.5]	27.3 [27.2-27.4]	29.5 [29.5-29.6]	30.5 [30.5-30.5]	31.3 [31.3-31.4]	32.7 [32.7-32.8]
31	25.2 [25.1-25.4]	28.9 [28.9-29.0]	30.1 [30.1-30.2]	31.1 [31.1-31.1]	32.5 [32.5-32.5]	27.3 [27.2-27.4]	29.5 [29.5-29.6]	30.5 [30.5-30.5]	31.3 [31.3-31.4]	32.7 [32.7-32.8]
32	25.2 [25.1-25.3]	28.9 [28.9-28.9]	30.1 [30.1-30.1]	31.1 [31.1-31.1]	32.5 [32.5-32.6]	27.3 [27.2-27.4]	29.6 [29.5-29.6]	30.5 [30.5-30.5]	31.4 [31.3-31.4]	32.8 [32.7-32.8]
33	25.1 [24.9-25.2]	28.9 [28.9-28.9]	30.1 [30.1-30.1]	31.1 [31.1-31.1]	32.5 [32.5-32.6]	27.3 [27.2-27.3]	29.6 [29.5-29.6]	30.5 [30.5-30.6]	31.4 [31.4-31.4]	32.8 [32.7-32.8]
34	24.9 [24.8-25.0]	28.9 [28.8-28.9]	30.1 [30.1-30.1]	31.1 [31.1-31.1]	32.6 [32.5-32.6]	27.3 [27.2-27.4]	29.6 [29.6-29.6]	30.6 [30.5-30.6]	31.4 [31.4-31.5]	32.8 [32.8-32.9]

35	24.8 [24.7-24.9]	28.8 [28.8-28.9]	30.1 [30.1-30.1]	31.1 [31.1-31.1]	32.6 [32.6-32.6]	27.3 [27.2-27.4]	29.6 [29.6-29.7]	30.6 [30.6-30.6]	31.5 [31.4-31.5]	32.9 [32.8-32.9]
36	24.6 [24.5-24.8]	28.8 [28.8-28.8]	30.1 [30.1-30.1]	31.1 [31.1-31.2]	32.6 [32.6-32.7]	27.3 [27.2-27.4]	29.6 [29.6-29.7]	30.6 [30.6-30.7]	31.5 [31.5-31.5]	32.9 [32.9-33.0]
37	24.5 [24.4-24.6]	28.8 [28.7-28.8]	30.1 [30.1-30.1]	31.2 [31.1-31.2]	32.7 [32.7-32.7]	27.3 [27.2-27.4]	29.7 [29.7-29.7]	30.7 [30.6-30.7]	31.5 [31.5-31.6]	33.0 [32.9-33.0]
38	24.4 [24.2-24.5]	28.8 [28.7-28.8]	30.1 [30.1-30.1]	31.2 [31.2-31.2]	32.7 [32.7-32.8]	27.3 [27.2-27.4]	29.7 [29.7-29.8]	30.7 [30.7-30.7]	31.6 [31.6-31.6]	33.0 [33.0-33.1]
39	24.2 [24.1-24.4]	28.7 [28.7-28.8]	30.1 [30.1-30.2]	31.2 [31.2-31.2]	32.8 [32.7-32.8]	27.3 [27.2-27.4]	29.8 [29.7-29.8]	30.8 [30.7-30.8]	31.6 [31.6-31.7]	33.1 [33.1-33.1]
40	24.1 [24.0-24.3]	28.7 [28.7-28.8]	30.1 [30.1-30.2]	31.2 [31.2-31.3]	32.8 [32.8-32.9]	27.3 [27.2-27.4]	29.8 [29.8-29.8]	30.8 [30.8-30.8]	31.7 [31.7-31.7]	33.2 [33.1-33.2]
41	24.0 [23.9-24.1]	28.7 [28.7-28.8]	30.1 [30.1-30.2]	31.3 [31.2-31.3]	32.9 [32.8-32.9]	27.3 [27.3-27.4]	29.8 [29.8-29.9]	30.9 [30.8-30.9]	31.8 [31.7-31.8]	33.2 [33.2-33.3]
42	23.9 [23.8-24.1]	28.7 [28.7-28.8]	30.2 [30.1-30.2]	31.3 [31.3-31.3]	32.9 [32.9-32.9]	27.4 [27.3-27.5]	29.9 [29.8-29.9]	30.9 [30.9-30.9]	31.8 [31.8-31.8]	33.3 [33.2-33.3]
43	23.9 [23.8-24.1]	28.7 [28.7-28.8]	30.2 [30.2-30.2]	31.3 [31.3-31.3]	32.9 [32.9-33.0]	27.4 [27.3-27.5]	29.9 [29.9-30.0]	31.0 [30.9-31.0]	31.9 [31.8-31.9]	33.3 [33.3-33.4]
44	24.0 [23.8-24.1]	28.7 [28.7-28.8]	30.2 [30.2-30.2]	31.3 [31.3-31.4]	33.0 [32.9-33.0]	27.4 [27.3-27.5]	30.0 [29.9-30.0]	31.0 [31.0-31.0]	31.9 [31.9-31.9]	33.4 [33.4-33.4]
45	24.0 [23.9-24.2]	28.8 [28.7-28.8]	30.2 [30.2-30.2]	31.3 [31.3-31.4]	33.0 [32.9-33.0]	27.4 [27.3-27.5]	30.0 [29.9-30.0]	31.0 [31.0-31.1]	32.0 [31.9-32.0]	33.5 [33.4-33.5]
46	24.1 [24.0-24.3]	28.8 [28.8-28.9]	30.2 [30.2-30.3]	31.3 [31.3-31.4]	33.0 [32.9-33.0]	27.4 [27.3-27.5]	30.0 [30.0-30.1]	31.1 [31.0-31.1]	32.0 [32.0-32.0]	33.5 [33.5-33.6]
47	24.3 [24.2-24.5]	28.9 [28.8-28.9]	30.3 [30.2-30.3]	31.4 [31.3-31.4]	32.9 [32.9-33.0]	27.4 [27.3-27.5]	30.1 [30.0-30.1]	31.1 [31.1-31.2]	32.1 [32.0-32.1]	33.6 [33.5-33.6]
48	24.5 [24.3-24.7]	28.9 [28.9-29.0]	30.3 [30.3-30.3]	31.3 [31.3-31.4]	32.9 [32.9-33.0]	27.4 [27.3-27.6]	30.1 [30.0-30.1]	31.2 [31.1-31.2]	32.1 [32.1-32.2]	33.6 [33.6-33.7]
49	24.8 [24.6-24.9]	29.0 [28.9-29.0]	30.3 [30.3-30.3]	31.3 [31.3-31.4]	32.9 [32.8-32.9]	27.4 [27.3-27.6]	30.1 [30.1-30.2]	31.2 [31.2-31.3]	32.1 [32.1-32.2]	33.7 [33.6-33.7]
50	25.0 [24.9-25.2]	29.1 [29.0-29.1]	30.3 [30.3-30.4]	31.3 [31.3-31.4]	32.8 [32.8-32.9]	27.5 [27.3-27.6]	30.2 [30.1-30.2]	31.2 [31.2-31.3]	32.2 [32.1-32.3]	33.7 [33.7-33.8]
51	25.3 [25.1-25.5]	29.1 [29.1-29.2]	30.4 [30.3-30.4]	31.3 [31.3-31.4]	32.8 [32.7-32.8]	27.5 [27.3-27.6]	30.2 [30.1-30.2]	31.3 [31.2-31.3]	32.2 [32.2-32.3]	33.8 [33.7-33.9]
52	25.6 [25.4-25.8]	29.2 [29.2-29.3]	30.4 [30.4-30.4]	31.3 [31.3-31.4]	32.7 [32.7-32.8]	27.5 [27.3-27.6]	30.2 [30.1-30.3]	31.3 [31.3-31.4]	32.3 [32.2-32.3]	33.8 [33.8-33.9]
53	25.9 [25.7-26.1]	29.3 [29.2-29.3]	30.4 [30.4-30.4]	31.3 [31.3-31.4]	32.7 [32.7-32.8]	27.5 [27.3-27.6]	30.2 [30.2-30.3]	31.3 [31.3-31.4]	32.3 [32.3-32.4]	33.9 [33.8-34.0]
54	26.1 [25.9-26.3]	29.4 [29.3-29.4]	30.4 [30.4-30.5]	31.3 [31.3-31.4]	32.7 [32.6-32.7]	27.5 [27.3-27.6]	30.2 [30.2-30.3]	31.4 [31.3-31.4]	32.3 [32.3-32.4]	33.9 [33.8-34.0]
55	26.3 [26.1-26.5]	29.4 [29.4-29.5]	30.5 [30.4-30.5]	31.3 [31.3-31.4]	32.7 [32.6-32.7]	27.4 [27.3-27.6]	30.3 [30.2-30.3]	31.4 [31.3-31.4]	32.4 [32.3-32.4]	34.0 [33.9-34.0]
56	26.5 [26.3-26.7]	29.5 [29.4-29.5]	30.5 [30.5-30.5]	31.4 [31.3-31.4]	32.6 [32.6-32.7]	27.4 [27.3-27.6]	30.3 [30.2-30.3]	31.4 [31.4-31.5]	32.4 [32.3-32.5]	34.0 [33.9-34.1]
57	26.6 [26.4-26.8]	29.5 [29.5-29.6]	30.5 [30.5-30.6]	31.4 [31.3-31.4]	32.6 [32.6-32.7]	27.4 [27.2-27.6]	30.3 [30.2-30.4]	31.4 [31.4-31.5]	32.4 [32.4-32.5]	34.0 [33.9-34.1]
58	26.8 [26.5-26.9]	29.6 [29.5-29.6]	30.6 [30.5-30.6]	31.4 [31.3-31.4]	32.7 [32.6-32.7]	27.4 [27.2-27.6]	30.3 [30.2-30.4]	31.5 [31.4-31.5]	32.4 [32.4-32.5]	34.0 [34.0-34.1]

59	26.8 [26.6-27.0]	29.6 [29.6-29.7]	30.6 [30.6-30.6]	31.4 [31.4-31.5]	32.7 [32.6-32.7]	27.4 [27.2-27.6]	30.3 [30.2-30.4]	31.5 [31.4-31.5]	32.5 [32.4-32.5]	34.1 [34.0-34.2]
60	26.9 [26.7-27.1]	29.6 [29.6-29.7]	30.6 [30.6-30.7]	31.4 [31.4-31.5]	32.7 [32.6-32.7]	27.4 [27.2-27.6]	30.3 [30.2-30.4]	31.5 [31.4-31.5]	32.5 [32.4-32.5]	34.1 [34.0-34.2]
61	27.0 [26.8-27.2]	29.7 [29.6-29.7]	30.6 [30.6-30.7]	31.5 [31.4-31.5]	32.7 [32.6-32.8]	27.4 [27.1-27.6]	30.3 [30.2-30.4]	31.5 [31.4-31.6]	32.5 [32.4-32.6]	34.1 [34.0-34.2]
62	27.0 [26.8-27.2]	29.7 [29.6-29.8]	30.7 [30.6-30.7]	31.5 [31.4-31.5]	32.7 [32.6-32.8]	27.3 [27.1-27.6]	30.3 [30.2-30.4]	31.5 [31.4-31.6]	32.5 [32.4-32.6]	34.1 [34.1-34.2]
63	27.1 [26.8-27.3]	29.7 [29.7-29.8]	30.7 [30.7-30.8]	31.5 [31.4-31.6]	32.7 [32.7-32.8]	27.3 [27.1-27.6]	30.3 [30.2-30.4]	31.5 [31.4-31.6]	32.5 [32.5-32.6]	34.2 [34.1-34.3]
64	27.1 [26.9-27.4]	29.8 [29.7-29.8]	30.7 [30.7-30.8]	31.5 [31.5-31.6]	32.8 [32.7-32.8]	27.3 [27.0-27.6]	30.3 [30.2-30.4]	31.5 [31.4-31.6]	32.5 [32.5-32.6]	34.2 [34.1-34.3]
65	27.2 [26.9-27.4]	29.8 [29.7-29.9]	30.8 [30.7-30.8]	31.5 [31.5-31.6]	32.8 [32.7-32.9]	27.3 [27.0-27.6]	30.3 [30.2-30.4]	31.5 [31.4-31.6]	32.5 [32.5-32.6]	34.2 [34.1-34.3]
66	27.2 [27.0-27.5]	29.8 [29.8-29.9]	30.8 [30.7-30.8]	31.6 [31.5-31.6]	32.8 [32.7-32.9]	27.2 [26.9-27.5]	30.3 [30.2-30.4]	31.5 [31.4-31.6]	32.5 [32.5-32.6]	34.2 [34.1-34.3]
67	27.3 [27.0-27.6]	29.9 [29.8-30.0]	30.8 [30.7-30.9]	31.6 [31.5-31.7]	32.8 [32.7-32.9]	27.2 [26.9-27.5]	30.3 [30.2-30.4]	31.5 [31.4-31.6]	32.5 [32.5-32.6]	34.2 [34.1-34.3]
68	27.3 [27.0-27.6]	29.9 [29.8-30.0]	30.8 [30.8-30.9]	31.6 [31.5-31.7]	32.8 [32.7-32.9]	27.2 [26.8-27.5]	30.3 [30.2-30.4]	31.5 [31.4-31.6]	32.6 [32.5-32.6]	34.2 [34.1-34.3]
69	27.4 [27.1-27.6]	29.9 [29.8-30.0]	30.8 [30.8-30.9]	31.6 [31.5-31.7]	32.8 [32.7-33.0]	27.1 [26.8-27.5]	30.3 [30.2-30.4]	31.5 [31.4-31.6]	32.6 [32.4-32.7]	34.2 [34.1-34.4]
70	27.4 [27.1-27.7]	29.9 [29.8-30.0]	30.9 [30.8-31.0]	31.6 [31.6-31.7]	32.8 [32.7-33.0]	27.1 [26.8-27.5]	30.3 [30.1-30.4]	31.5 [31.4-31.6]	32.6 [32.4-32.7]	34.2 [34.1-34.4]
71	27.5 [27.2-27.8]	30.0 [29.9-30.1]	30.9 [30.8-31.0]	31.6 [31.6-31.8]	32.8 [32.7-33.0]	27.1 [26.7-27.5]	30.3 [30.1-30.4]	31.5 [31.4-31.6]	32.5 [32.4-32.7]	34.2 [34.1-34.4]
72	27.5 [27.2-27.9]	30.0 [29.9-30.1]	30.9 [30.8-31.0]	31.7 [31.6-31.8]	32.9 [32.7-33.0]	27.0 [26.6-27.4]	30.2 [30.1-30.4]	31.5 [31.3-31.6]	32.5 [32.4-32.7]	34.2 [34.1-34.4]
73	27.6 [27.2-28.0]	30.0 [29.9-30.1]	30.9 [30.8-31.0]	31.7 [31.6-31.8]	32.9 [32.7-33.0]	27.0 [26.6-27.4]	30.2 [30.0-30.4]	31.5 [31.3-31.6]	32.5 [32.4-32.7]	34.2 [34.1-34.4]
74	27.6 [27.2-28.1]	30.0 [29.9-30.2]	30.9 [30.8-31.1]	31.7 [31.6-31.8]	32.9 [32.7-33.0]	26.9 [26.5-27.3]	30.2 [30.0-30.4]	31.5 [31.3-31.6]	32.5 [32.3-32.7]	34.2 [34.0-34.4]
75	27.6 [27.2-28.2]	30.1 [29.9-30.2]	31.0 [30.9-31.1]	31.7 [31.6-31.9]	32.9 [32.7-33.1]	26.9 [26.5-27.3]	30.2 [29.9-30.4]	31.4 [31.2-31.6]	32.5 [32.3-32.7]	34.2 [34.0-34.4]
76	27.7 [27.2-28.2]	30.1 [29.9-30.3]	31.0 [30.9-31.1]	31.7 [31.6-31.9]	32.9 [32.7-33.1]	26.8 [26.4-27.3]	30.2 [29.9-30.4]	31.4 [31.2-31.6]	32.5 [32.3-32.7]	34.2 [34.0-34.5]
77	27.7 [27.2-28.3]	30.1 [29.9-30.3]	31.0 [30.9-31.2]	31.7 [31.6-31.9]	32.9 [32.7-33.1]	26.8 [26.3-27.2]	30.1 [29.8-30.4]	31.4 [31.1-31.6]	32.5 [32.2-32.7]	34.2 [34.0-34.5]
78	27.8 [27.2-28.4]	30.1 [29.9-30.3]	31.0 [30.9-31.2]	31.8 [31.6-32.0]	32.9 [32.7-33.1]	26.7 [26.3-27.2]	30.1 [29.8-30.4]	31.4 [31.1-31.6]	32.5 [32.2-32.7]	34.2 [33.9-34.5]
79	27.8 [27.2-28.5]	30.2 [30.0-30.4]	31.0 [30.9-31.2]	31.8 [31.6-32.0]	32.9 [32.7-33.2]	26.7 [26.2-27.2]	30.1 [29.7-30.4]	31.4 [31.1-31.6]	32.5 [32.2-32.7]	34.2 [33.9-34.5]
80	27.8 [27.1-28.6]	30.2 [30.0-30.4]	31.1 [30.9-31.2]	31.8 [31.6-32.0]	33.0 [32.6-33.2]	26.7 [26.0-27.2]	30.1 [29.7-30.4]	31.4 [31.0-31.6]	32.5 [32.1-32.7]	34.2 [33.9-34.5]

Supplemental Table 5. Model-predicted sex- and age-specific next-generation reference intervals for MCHC.

Age	Female					Male				
	Q2.5	Q25	Q50	Q75	Q97.5	Q2.5	Q25	Q50	Q75	Q97.5
18	318 [316-320]	334 [333-335]	340 [340-341]	346 [346-347]	357 [355-358]	329 [328-329]	339 [338-339]	344 [344-345]	350 [349-350]	360 [360-361]
19	318 [317-320]	334 [333-334]	340 [340-341]	346 [346-347]	356 [355-357]	329 [328-329]	339 [339-339]	344 [344-345]	350 [349-350]	360 [360-361]
20	319 [317-320]	334 [333-334]	340 [340-341]	346 [346-347]	356 [355-357]	329 [328-329]	339 [339-339]	344 [344-345]	350 [349-350]	360 [360-361]
21	319 [317-320]	334 [333-334]	340 [340-340]	346 [345-346]	356 [355-356]	329 [328-329]	339 [339-339]	344 [344-345]	350 [350-350]	360 [360-361]
22	319 [318-320]	334 [333-334]	340 [340-340]	346 [345-346]	356 [355-356]	328 [328-329]	339 [339-339]	344 [344-345]	350 [350-350]	361 [360-361]
23	319 [318-320]	333 [333-334]	340 [340-340]	346 [345-346]	355 [355-356]	328 [328-329]	339 [339-339]	344 [344-345]	350 [350-350]	361 [360-361]
24	319 [318-320]	333 [333-334]	340 [340-340]	346 [345-346]	355 [355-356]	328 [328-329]	339 [339-339]	344 [344-345]	350 [350-350]	361 [360-361]
25	319 [318-319]	333 [333-334]	340 [339-340]	345 [345-346]	355 [355-355]	328 [328-329]	339 [339-339]	344 [344-345]	350 [350-350]	361 [360-361]
26	319 [318-319]	333 [333-333]	340 [339-340]	345 [345-345]	355 [355-355]	328 [328-329]	339 [339-339]	344 [344-345]	350 [350-350]	361 [360-361]
27	319 [318-319]	333 [333-333]	339 [339-340]	345 [345-345]	355 [354-355]	328 [328-329]	339 [339-339]	344 [344-345]	350 [350-350]	361 [360-361]
28	319 [318-319]	333 [333-333]	339 [339-340]	345 [345-345]	355 [354-355]	328 [328-329]	339 [339-339]	344 [344-345]	350 [350-350]	361 [361-361]
29	319 [318-319]	333 [333-333]	339 [339-340]	345 [345-345]	355 [354-355]	328 [328-329]	339 [339-339]	344 [344-345]	350 [350-350]	361 [361-361]
30	318 [318-319]	333 [333-333]	339 [339-339]	345 [345-345]	355 [354-355]	328 [328-329]	339 [339-339]	344 [344-345]	350 [350-350]	361 [361-361]
31	318 [318-319]	333 [333-333]	339 [339-339]	345 [345-345]	355 [355-355]	328 [328-328]	339 [339-339]	344 [344-345]	350 [350-350]	361 [361-361]
32	318 [318-318]	333 [333-333]	339 [339-339]	345 [345-345]	355 [355-355]	328 [328-328]	339 [339-339]	344 [344-345]	350 [350-350]	361 [361-361]
33	318 [317-318]	333 [333-333]	339 [339-339]	345 [345-345]	355 [355-355]	328 [328-328]	339 [339-339]	344 [344-345]	350 [350-350]	361 [361-361]
34	317 [317-318]	333 [332-333]	339 [339-339]	345 [345-345]	355 [355-355]	328 [328-328]	339 [339-339]	344 [344-345]	350 [350-350]	361 [361-361]

35	317 [317-317]	332 [332-333]	339 [339-339]	345 [345-345]	355 [355-356]	328 [328-328]	339 [339-339]	344 [344-345]	350 [350-350]	361 [361-361]
36	316 [316-317]	332 [332-332]	339 [339-339]	345 [345-345]	355 [355-356]	328 [328-328]	339 [339-339]	344 [344-345]	350 [350-350]	361 [361-361]
37	316 [316-316]	332 [332-332]	339 [339-339]	345 [345-345]	356 [355-356]	328 [327-328]	339 [338-339]	344 [344-344]	350 [350-350]	361 [361-361]
38	315 [315-316]	332 [332-332]	339 [339-339]	345 [345-345]	356 [355-356]	328 [327-328]	339 [338-339]	344 [344-344]	350 [350-350]	361 [361-361]
39	315 [315-316]	332 [331-332]	339 [339-339]	345 [345-345]	356 [355-356]	328 [327-328]	339 [338-339]	344 [344-344]	350 [350-350]	361 [361-361]
40	315 [314-315]	332 [331-332]	339 [339-339]	345 [345-345]	356 [356-356]	328 [327-328]	338 [338-339]	344 [344-344]	350 [350-350]	361 [361-361]
41	314 [314-315]	331 [331-332]	339 [339-339]	345 [345-345]	356 [356-356]	327 [327-328]	338 [338-339]	344 [344-344]	350 [350-350]	361 [361-361]
42	314 [313-315]	331 [331-332]	339 [338-339]	345 [345-345]	356 [356-356]	327 [327-328]	338 [338-338]	344 [344-344]	350 [350-350]	361 [361-361]
43	314 [313-315]	331 [331-332]	339 [338-339]	345 [345-345]	356 [356-356]	327 [327-328]	338 [338-338]	344 [344-344]	350 [350-350]	361 [361-361]
44	314 [313-315]	331 [331-332]	339 [338-339]	345 [345-345]	356 [356-356]	327 [327-327]	338 [338-338]	344 [344-344]	350 [349-350]	361 [360-361]
45	314 [314-315]	331 [331-332]	339 [338-339]	345 [345-345]	356 [356-356]	327 [327-327]	338 [338-338]	344 [344-344]	350 [349-350]	361 [360-361]
46	315 [314-315]	332 [331-332]	339 [339-339]	345 [345-345]	356 [355-356]	327 [327-327]	338 [338-338]	344 [344-344]	350 [349-350]	361 [360-361]
47	315 [315-316]	332 [331-332]	339 [339-339]	345 [345-345]	356 [356-356]	327 [326-327]	338 [338-338]	344 [343-344]	349 [349-350]	361 [360-361]
48	316 [315-316]	332 [332-332]	339 [339-339]	345 [345-346]	356 [355-356]	327 [326-327]	338 [337-338]	344 [343-344]	349 [349-350]	361 [360-361]
49	316 [316-317]	332 [332-333]	339 [339-339]	345 [345-346]	356 [355-356]	327 [326-327]	338 [337-338]	343 [343-344]	349 [349-350]	361 [360-361]
50	317 [316-317]	333 [332-333]	339 [339-340]	346 [345-346]	356 [355-356]	326 [326-327]	338 [337-338]	343 [343-344]	349 [349-349]	360 [360-361]
51	317 [317-318]	333 [333-333]	340 [339-340]	346 [345-346]	356 [355-356]	326 [326-327]	337 [337-338]	343 [343-343]	349 [349-349]	360 [360-361]
52	318 [317-319]	333 [333-333]	340 [340-340]	346 [346-346]	356 [355-356]	326 [326-327]	337 [337-338]	343 [343-343]	349 [349-349]	360 [360-361]
53	319 [318-319]	334 [333-334]	340 [340-340]	346 [346-346]	356 [355-356]	326 [326-326]	337 [337-337]	343 [343-343]	349 [349-349]	360 [360-361]
54	319 [319-320]	334 [333-334]	340 [340-340]	346 [346-346]	356 [355-356]	326 [325-326]	337 [337-337]	343 [343-343]	349 [349-349]	360 [360-361]
55	320 [319-320]	334 [334-334]	340 [340-341]	346 [346-346]	356 [355-356]	326 [325-326]	337 [337-337]	343 [343-343]	349 [348-349]	360 [359-360]
56	320 [319-321]	334 [334-335]	340 [340-341]	346 [346-346]	356 [355-356]	326 [325-326]	337 [337-337]	343 [342-343]	349 [348-349]	360 [359-360]
57	320 [320-321]	334 [334-335]	341 [340-341]	346 [346-346]	356 [355-356]	325 [325-326]	337 [336-337]	343 [342-343]	348 [348-349]	360 [359-360]
58	321 [320-321]	335 [334-335]	341 [340-341]	346 [346-347]	356 [355-356]	325 [325-326]	337 [336-337]	342 [342-343]	348 [348-349]	360 [359-360]

59	321 [320-322]	335 [334-335]	341 [340-341]	346 [346-347]	356 [355-356]	325 [325-326]	336 [336-337]	342 [342-343]	348 [348-349]	360 [359-360]
60	321 [320-322]	335 [334-335]	341 [340-341]	346 [346-347]	355 [355-356]	325 [324-326]	336 [336-337]	342 [342-343]	348 [348-349]	360 [359-360]
61	321 [320-322]	335 [334-335]	341 [340-341]	346 [346-347]	355 [355-356]	325 [324-326]	336 [336-336]	342 [342-342]	348 [348-348]	359 [359-360]
62	321 [320-322]	335 [334-335]	341 [340-341]	346 [346-346]	355 [355-356]	325 [324-325]	336 [336-336]	342 [342-342]	348 [347-348]	359 [359-360]
63	321 [320-322]	335 [334-335]	341 [340-341]	346 [346-346]	355 [354-356]	324 [324-325]	336 [335-336]	342 [341-342]	348 [347-348]	359 [358-360]
64	321 [321-322]	335 [334-335]	340 [340-341]	346 [345-346]	355 [354-355]	324 [324-325]	336 [335-336]	342 [341-342]	348 [347-348]	359 [358-360]
65	321 [320-322]	334 [334-335]	340 [340-341]	346 [345-346]	355 [354-355]	324 [323-325]	335 [335-336]	341 [341-342]	347 [347-348]	359 [358-360]
66	322 [320-322]	334 [334-335]	340 [340-341]	345 [345-346]	354 [354-355]	324 [323-325]	335 [335-336]	341 [341-342]	347 [347-348]	359 [358-360]
67	322 [320-323]	334 [334-335]	340 [339-340]	345 [345-346]	354 [353-355]	324 [323-325]	335 [335-336]	341 [341-342]	347 [346-348]	359 [358-360]
68	322 [320-323]	334 [334-335]	340 [339-340]	345 [344-346]	354 [353-355]	324 [323-325]	335 [334-336]	341 [340-342]	347 [346-348]	358 [357-359]
69	322 [320-323]	334 [333-335]	340 [339-340]	345 [344-345]	353 [352-354]	323 [323-324]	335 [334-335]	341 [340-341]	347 [346-348]	358 [357-359]
70	322 [321-323]	334 [333-335]	339 [339-340]	344 [344-345]	353 [352-354]	323 [322-324]	335 [334-335]	341 [340-341]	347 [346-347]	358 [357-359]
71	322 [321-323]	334 [333-335]	339 [339-340]	344 [343-345]	353 [351-354]	323 [322-324]	334 [334-335]	340 [340-341]	346 [346-347]	358 [357-359]
72	322 [321-324]	334 [333-335]	339 [338-340]	344 [343-345]	352 [351-353]	323 [322-324]	334 [334-335]	340 [340-341]	346 [345-347]	358 [356-359]
73	322 [321-324]	334 [333-335]	339 [338-340]	344 [343-345]	352 [351-353]	323 [322-324]	334 [333-335]	340 [339-341]	346 [345-347]	358 [356-359]
74	322 [321-324]	334 [333-335]	339 [338-340]	343 [342-344]	351 [350-353]	322 [321-324]	334 [333-335]	340 [339-341]	346 [345-347]	357 [356-359]
75	323 [321-324]	334 [333-335]	339 [338-339]	343 [342-344]	351 [350-352]	322 [321-324]	334 [333-335]	340 [339-341]	346 [345-347]	357 [356-359]
76	323 [321-325]	333 [332-334]	338 [337-339]	343 [342-344]	351 [349-352]	322 [321-323]	333 [333-334]	339 [338-341]	346 [344-347]	357 [355-359]
77	323 [321-325]	333 [332-335]	338 [337-339]	343 [342-344]	350 [349-352]	322 [320-323]	333 [332-334]	339 [338-340]	345 [344-347]	357 [355-359]
78	323 [321-325]	333 [332-335]	338 [337-339]	342 [341-344]	350 [348-352]	321 [320-323]	333 [332-334]	339 [338-340]	345 [344-347]	357 [355-359]
79	323 [321-326]	333 [332-335]	338 [337-339]	342 [341-344]	350 [348-351]	321 [320-323]	333 [332-334]	339 [338-340]	345 [344-346]	357 [355-358]
80	323 [321-326]	333 [332-335]	338 [337-339]	342 [340-343]	349 [347-351]	321 [320-323]	333 [331-334]	339 [337-340]	345 [343-346]	356 [354-358]

Supplemental Table 6. Model-predicted sex- and age-specific next-generation reference intervals for WBC.

Age	Female					Male				
	Q2.5	Q25	Q50	Q75	Q97.5	Q2.5	Q25	Q50	Q75	Q97.5
18	3.95 [3.87-4.04]	5.27 [5.17-5.38]	6.14 [6.03-6.26]	7.16 [7.04-7.31]	9.61 [9.41-9.84]	4.13 [3.99-4.22]	5.29 [5.19-5.39]	6.07 [5.98-6.17]	6.99 [6.87-7.12]	9.28 [9.10-9.58]
19	3.92 [3.85-4.00]	5.23 [5.15-5.31]	6.10 [6.00-6.19]	7.11 [7.00-7.22]	9.55 [9.39-9.75]	4.11 [4.00-4.20]	5.28 [5.19-5.37]	6.06 [5.98-6.15]	6.98 [6.87-7.09]	9.28 [9.12-9.52]
20	3.88 [3.83-3.95]	5.19 [5.12-5.25]	6.05 [5.98-6.12]	7.06 [6.98-7.14]	9.50 [9.35-9.65]	4.10 [4.01-4.18]	5.27 [5.20-5.35]	6.05 [5.97-6.12]	6.97 [6.87-7.07]	9.27 [9.13-9.46]
21	3.85 [3.80-3.90]	5.15 [5.10-5.20]	6.01 [5.95-6.06]	7.01 [6.95-7.08]	9.44 [9.31-9.57]	4.09 [4.01-4.16]	5.26 [5.20-5.33]	6.04 [5.97-6.11]	6.96 [6.87-7.05]	9.27 [9.14-9.40]
22	3.82 [3.78-3.86]	5.11 [5.07-5.15]	5.97 [5.92-6.01]	6.97 [6.92-7.03]	9.39 [9.30-9.49]	4.08 [4.01-4.14]	5.25 [5.19-5.31]	6.03 [5.97-6.09]	6.96 [6.88-7.03]	9.26 [9.14-9.38]
23	3.79 [3.76-3.83]	5.08 [5.05-5.11]	5.93 [5.90-5.97]	6.93 [6.89-6.98]	9.35 [9.26-9.44]	4.07 [4.01-4.12]	5.24 [5.19-5.30]	6.02 [5.96-6.07]	6.95 [6.87-7.01]	9.26 [9.12-9.37]
24	3.77 [3.74-3.80]	5.05 [5.02-5.08]	5.90 [5.86-5.93]	6.89 [6.86-6.94]	9.31 [9.24-9.40]	4.06 [4.01-4.10]	5.23 [5.18-5.28]	6.01 [5.96-6.05]	6.94 [6.87-7.00]	9.26 [9.13-9.36]
25	3.74 [3.72-3.77]	5.02 [4.99-5.04]	5.87 [5.84-5.89]	6.86 [6.83-6.90]	9.27 [9.21-9.37]	4.04 [4.00-4.09]	5.22 [5.18-5.26]	6.00 [5.96-6.04]	6.93 [6.87-6.98]	9.26 [9.14-9.36]
26	3.72 [3.70-3.74]	5.00 [4.98-5.02]	5.84 [5.82-5.87]	6.84 [6.81-6.87]	9.25 [9.19-9.33]	4.03 [3.99-4.07]	5.21 [5.17-5.24]	5.99 [5.95-6.02]	6.92 [6.87-6.96]	9.26 [9.16-9.35]

27	3.70<br>[3.68-3.72]	4.98<br>[4.96-5.00]	5.82<br>[5.80-5.84]	6.82<br>[6.79-6.85]	9.23<br>[9.17-9.30]	4.02<br>[3.98-4.05]	5.19<br>[5.16-5.22]	5.98<br>[5.95-6.01]	6.92<br>[6.87-6.96]	9.27 [9.17-9.35]
28	3.68<br>[3.66-3.70]	4.96<br>[4.94-4.98]	5.80<br>[5.78-5.82]	6.80<br>[6.77-6.83]	9.21<br>[9.16-9.28]	4.01<br>[3.97-4.04]	5.18<br>[5.16-5.21]	5.97<br>[5.94-6.00]	6.91<br>[6.88-6.95]	9.27 [9.18-9.36]
29	3.67<br>[3.65-3.68]	4.94<br>[4.92-4.96]	5.78<br>[5.76-5.81]	6.78<br>[6.75-6.81]	9.20<br>[9.15-9.26]	3.99<br>[3.96-4.02]	5.17<br>[5.15-5.19]	5.96<br>[5.94-5.99]	6.91<br>[6.87-6.95]	9.28 [9.18-9.36]
30	3.65<br>[3.63-3.67]	4.92<br>[4.90-4.94]	5.76<br>[5.74-5.79]	6.76<br>[6.73-6.79]	9.18<br>[9.13-9.24]	3.98<br>[3.95-4.01]	5.16<br>[5.14-5.18]	5.95<br>[5.93-5.98]	6.90<br>[6.87-6.94]	9.29 [9.18-9.37]
31	3.63<br>[3.61-3.65]	4.90<br>[4.88-4.92]	5.74<br>[5.72-5.77]	6.74<br>[6.71-6.77]	9.16<br>[9.11-9.22]	3.97<br>[3.94-4.00]	5.15<br>[5.13-5.17]	5.95<br>[5.92-5.97]	6.90<br>[6.86-6.93]	9.30 [9.21-9.37]
32	3.61<br>[3.59-3.62]	4.88<br>[4.86-4.90]	5.72<br>[5.70-5.74]	6.71<br>[6.69-6.75]	9.14<br>[9.08-9.20]	3.95<br>[3.93-3.98]	5.14<br>[5.12-5.16]	5.94<br>[5.92-5.96]	6.90<br>[6.87-6.93]	9.32 [9.22-9.39]
33	3.59<br>[3.57-3.60]	4.85<br>[4.83-4.87]	5.69<br>[5.67-5.72]	6.69<br>[6.66-6.72]	9.11<br>[9.05-9.17]	3.94<br>[3.91-3.96]	5.13<br>[5.11-5.15]	5.93<br>[5.91-5.96]	6.90<br>[6.86-6.93]	9.33 [9.24-9.40]
34	3.56<br>[3.54-3.58]	4.83<br>[4.81-4.85]	5.67<br>[5.64-5.69]	6.66<br>[6.63-6.69]	9.08<br>[9.02-9.14]	3.93<br>[3.90-3.95]	5.12<br>[5.10-5.15]	5.93<br>[5.90-5.95]	6.90<br>[6.86-6.93]	9.35 [9.26-9.42]
35	3.54<br>[3.52-3.56]	4.80<br>[4.78-4.82]	5.64<br>[5.61-5.67]	6.63<br>[6.60-6.66]	9.05<br>[8.99-9.11]	3.91<br>[3.89-3.94]	5.11<br>[5.09-5.14]	5.92<br>[5.89-5.95]	6.90<br>[6.86-6.93]	9.37 [9.28-9.44]
36	3.52<br>[3.50-3.54]	4.78<br>[4.76-4.80]	5.62<br>[5.59-5.64]	6.60<br>[6.57-6.64]	9.02<br>[8.95-9.09]	3.90<br>[3.88-3.93]	5.11<br>[5.08-5.13]	5.92<br>[5.89-5.95]	6.90<br>[6.86-6.93]	9.39 [9.30-9.46]
37	3.51<br>[3.48-3.53]	4.76<br>[4.73-4.78]	5.59<br>[5.56-5.62]	6.58<br>[6.54-6.62]	9.00<br>[8.93-9.07]	3.89<br>[3.87-3.92]	5.10<br>[5.07-5.12]	5.92<br>[5.89-5.95]	6.90<br>[6.86-6.94]	9.41 [9.33-9.48]
38	3.50<br>[3.47-3.52]	4.75<br>[4.72-4.77]	5.58<br>[5.55-5.61]	6.56<br>[6.53-6.60]	8.98<br>[8.91-9.05]	3.88<br>[3.86-3.91]	5.09<br>[5.07-5.12]	5.91<br>[5.89-5.94]	6.91<br>[6.86-6.94]	9.43 [9.36-9.50]
39	3.49<br>[3.46-3.51]	4.73<br>[4.71-4.76]	5.57<br>[5.54-5.59]	6.55<br>[6.51-6.59]	8.96<br>[8.89-9.04]	3.87<br>[3.85-3.90]	5.09<br>[5.06-5.11]	5.91<br>[5.88-5.94]	6.91<br>[6.87-6.94]	9.45 [9.38-9.53]

40	3.48 [3.46-3.51]	4.73 [4.70-4.75]	5.56 [5.53-5.58]	6.54 [6.50-6.57]	8.95 [8.88-9.03]	3.87 [3.84-3.89]	5.08 [5.06-5.11]	5.91 [5.88-5.94]	6.91 [6.87-6.95]	9.47 [9.40-9.56]
41	3.48 [3.45-3.50]	4.72 [4.70-4.75]	5.55 [5.52-5.58]	6.54 [6.50-6.57]	8.94 [8.87-9.03]	3.86 [3.83-3.88]	5.08 [5.05-5.11]	5.91 [5.88-5.94]	6.92 [6.87-6.96]	9.50 [9.41-9.59]
42	3.47 [3.45-3.50]	4.72 [4.69-4.74]	5.55 [5.52-5.58]	6.53 [6.49-6.56]	8.93 [8.85-9.02]	3.85 [3.82-3.88]	5.08 [5.05-5.10]	5.91 [5.88-5.94]	6.93 [6.88-6.97]	9.52 [9.43-9.62]
43	3.47 [3.44-3.50]	4.71 [4.69-4.74]	5.54 [5.51-5.57]	6.52 [6.48-6.56]	8.92 [8.83-9.00]	3.84 [3.81-3.87]	5.07 [5.05-5.10]	5.91 [5.88-5.94]	6.93 [6.89-6.97]	9.55 [9.46-9.64]
44	3.47 [3.44-3.50]	4.71 [4.68-4.73]	5.53 [5.50-5.56]	6.51 [6.46-6.54]	8.90 [8.81-8.98]	3.84 [3.81-3.87]	5.07 [5.05-5.10]	5.92 [5.89-5.95]	6.94 [6.90-6.98]	9.57 [9.48-9.67]
45	3.46 [3.43-3.49]	4.69 [4.66-4.73]	5.52 [5.48-5.55]	6.49 [6.44-6.52]	8.86 [8.77-8.95]	3.83 [3.80-3.86]	5.07 [5.05-5.10]	5.92 [5.89-5.95]	6.95 [6.91-6.99]	9.60 [9.50-9.70]
46	3.45 [3.42-3.48]	4.68 [4.65-4.71]	5.49 [5.46-5.53]	6.46 [6.41-6.49]	8.82 [8.73-8.89]	3.83 [3.80-3.86]	5.07 [5.05-5.10]	5.92 [5.90-5.95]	6.96 [6.92-7.00]	9.63 [9.53-9.73]
47	3.44 [3.41-3.47]	4.66 [4.62-4.68]	5.47 [5.43-5.50]	6.42 [6.38-6.46]	8.76 [8.67-8.83]	3.82 [3.79-3.86]	5.07 [5.05-5.10]	5.93 [5.90-5.96]	6.97 [6.93-7.01]	9.66 [9.56-9.76]
48	3.42 [3.39-3.45]	4.63 [4.60-4.66]	5.43 [5.40-5.46]	6.38 [6.34-6.41]	8.69 [8.61-8.77]	3.82 [3.79-3.85]	5.07 [5.05-5.10]	5.94 [5.90-5.96]	6.98 [6.94-7.03]	9.70 [9.60-9.80]
49	3.40 [3.37-3.43]	4.60 [4.57-4.62]	5.39 [5.36-5.42]	6.33 [6.29-6.36]	8.61 [8.54-8.69]	3.82 [3.78-3.85]	5.08 [5.05-5.11]	5.94 [5.91-5.97]	7.00 [6.96-7.04]	9.73 [9.63-9.84]
50	3.38 [3.35-3.41]	4.57 [4.54-4.59]	5.35 [5.32-5.38]	6.28 [6.24-6.31]	8.53 [8.45-8.61]	3.81 [3.77-3.85]	5.08 [5.05-5.11]	5.95 [5.91-5.99]	7.01 [6.96-7.06]	9.77 [9.66-9.88]
51	3.36 [3.33-3.39]	4.53 [4.50-4.56]	5.31 [5.28-5.34]	6.23 [6.18-6.27]	8.46 [8.36-8.55]	3.81 [3.76-3.85]	5.08 [5.05-5.11]	5.95 [5.92-6.00]	7.02 [6.97-7.08]	9.80 [9.68-9.92]
52	3.35 [3.31-3.38]	4.51 [4.47-4.54]	5.28 [5.24-5.32]	6.18 [6.14-6.23]	8.39 [8.30-8.49]	3.80 [3.76-3.85]	5.08 [5.04-5.12]	5.96 [5.92-6.01]	7.04 [6.99-7.11]	9.84 [9.73-9.98]

53	3.33 [3.29-3.37]	4.49 [4.45-4.52]	5.25 [5.21-5.29]	6.15 [6.10-6.20]	8.34 [8.24-8.44]	3.80 [3.75-3.84]	5.08 [5.04-5.12]	5.97 [5.93-6.02]	7.05 [7.00-7.13]	9.87 [9.76-10.02]
54	3.32 [3.28-3.36]	4.47 [4.43-4.51]	5.23 [5.19-5.28]	6.13 [6.07-6.18]	8.30 [8.19-8.42]	3.80 [3.74-3.84]	5.08 [5.04-5.13]	5.97 [5.93-6.03]	7.06 [7.01-7.15]	9.91 [9.79-10.06]
55	3.32 [3.28-3.36]	4.46 [4.43-4.50]	5.22 [5.18-5.27]	6.12 [6.06-6.18]	8.28 [8.16-8.41]	3.79 [3.74-3.84]	5.08 [5.04-5.13]	5.98 [5.93-6.04]	7.07 [7.02-7.17]	9.94 [9.82-10.10]
56	3.32 [3.28-3.37]	4.47 [4.43-4.51]	5.23 [5.18-5.27]	6.12 [6.06-6.17]	8.28 [8.16-8.42]	3.79 [3.73-3.84]	5.09 [5.04-5.13]	5.98 [5.93-6.04]	7.09 [7.02-7.18]	9.97 [9.83-10.13]
57	3.33 [3.29-3.37]	4.48 [4.44-4.52]	5.24 [5.19-5.28]	6.13 [6.07-6.18]	8.30 [8.18-8.43]	3.79 [3.72-3.84]	5.09 [5.03-5.13]	5.99 [5.94-6.05]	7.10 [7.03-7.19]	10.00 [9.85-10.16]
58	3.34 [3.30-3.38]	4.50 [4.45-4.53]	5.26 [5.21-5.30]	6.15 [6.08-6.21]	8.33 [8.20-8.44]	3.78 [3.72-3.84]	5.09 [5.03-5.14]	5.99 [5.94-6.05]	7.11 [7.04-7.20]	10.03 [9.89-10.20]
59	3.36 [3.32-3.40]	4.52 [4.47-4.56]	5.28 [5.24-5.33]	6.18 [6.11-6.25]	8.37 [8.22-8.49]	3.78 [3.72-3.84]	5.09 [5.03-5.14]	6.00 [5.94-6.06]	7.11 [7.05-7.21]	10.05 [9.91-10.24]
60	3.38 [3.34-3.42]	4.54 [4.50-4.59]	5.31 [5.26-5.36]	6.22 [6.13-6.29]	8.42 [8.26-8.54]	3.78 [3.71-3.84]	5.09 [5.03-5.14]	6.00 [5.94-6.07]	7.12 [7.05-7.22]	10.07 [9.92-10.25]
61	3.40 [3.35-3.44]	4.57 [4.53-4.63]	5.34 [5.29-5.41]	6.26 [6.17-6.34]	8.48 [8.31-8.60]	3.78 [3.71-3.84]	5.09 [5.03-5.14]	6.00 [5.94-6.07]	7.13 [7.05-7.22]	10.09 [9.92-10.27]
62	3.41 [3.37-3.47]	4.59 [4.54-4.66]	5.37 [5.30-5.45]	6.29 [6.20-6.39]	8.53 [8.35-8.67]	3.78 [3.71-3.84]	5.09 [5.03-5.14]	6.01 [5.94-6.08]	7.13 [7.05-7.23]	10.11 [9.92-10.30]
63	3.43 [3.38-3.49]	4.62 [4.56-4.69]	5.40 [5.33-5.48]	6.33 [6.23-6.44]	8.58 [8.40-8.75]	3.77 [3.70-3.85]	5.09 [5.03-5.15]	6.01 [5.94-6.08]	7.14 [7.05-7.23]	10.12 [9.91-10.32]
64	3.44 [3.39-3.51]	4.64 [4.57-4.72]	5.43 [5.34-5.51]	6.36 [6.25-6.47]	8.63 [8.43-8.82]	3.77 [3.70-3.85]	5.09 [5.03-5.15]	6.01 [5.94-6.08]	7.14 [7.04-7.24]	10.14 [9.90-10.36]
65	3.46 [3.39-3.53]	4.66 [4.58-4.74]	5.45 [5.36-5.54]	6.39 [6.27-6.50]	8.67 [8.47-8.88]	3.77 [3.70-3.85]	5.09 [5.03-5.16]	6.01 [5.94-6.08]	7.15 [7.05-7.25]	10.15 [9.92-10.40]

66	3.46 [3.39-3.54]	4.67 [4.59-4.76]	5.47 [5.37-5.56]	6.41 [6.29-6.54]	8.71 [8.48-8.94]	3.77 [3.69-3.86]	5.09 [5.03-5.16]	6.02 [5.94-6.08]	7.15 [7.05-7.26]	10.15 [9.91-10.44]
67	3.47 [3.40-3.55]	4.68 [4.60-4.77]	5.48 [5.38-5.57]	6.43 [6.30-6.56]	8.74 [8.52-8.98]	3.77 [3.69-3.88]	5.10 [5.03-5.16]	6.02 [5.94-6.09]	7.16 [7.04-7.27]	10.16 [9.84-10.48]
68	3.47 [3.39-3.56]	4.68 [4.60-4.78]	5.49 [5.38-5.59]	6.44 [6.29-6.58]	8.76 [8.52-9.02]	3.77 [3.68-3.88]	5.10 [5.03-5.17]	6.02 [5.95-6.09]	7.16 [7.03-7.28]	10.17 [9.81-10.51]
69	3.47 [3.39-3.56]	4.69 [4.60-4.78]	5.50 [5.39-5.61]	6.45 [6.29-6.59]	8.78 [8.51-9.06]	3.78 [3.68-3.89]	5.10 [5.02-5.18]	6.02 [5.95-6.10]	7.16 [7.02-7.28]	10.17 [9.73-10.54]
70	3.47 [3.38-3.57]	4.69 [4.60-4.79]	5.50 [5.38-5.60]	6.46 [6.30-6.62]	8.80 [8.51-9.11]	3.78 [3.67-3.92]	5.10 [5.02-5.19]	6.02 [5.95-6.10]	7.16 [7.00-7.30]	10.17 [9.67-10.58]
71	3.46 [3.37-3.57]	4.69 [4.59-4.79]	5.50 [5.38-5.63]	6.46 [6.31-6.63]	8.82 [8.50-9.13]	3.78 [3.66-3.94]	5.10 [5.01-5.19]	6.02 [5.94-6.11]	7.16 [6.98-7.31]	10.18 [9.62-10.62]
72	3.46 [3.35-3.58]	4.68 [4.59-4.80]	5.50 [5.37-5.63]	6.47 [6.31-6.64]	8.83 [8.49-9.16]	3.78 [3.66-3.97]	5.10 [5.01-5.20]	6.03 [5.94-6.12]	7.17 [6.96-7.32]	10.18 [9.57-10.66]
73	3.45 [3.34-3.59]	4.68 [4.56-4.82]	5.50 [5.36-5.65]	6.47 [6.28-6.67]	8.84 [8.48-9.23]	3.78 [3.65-4.01]	5.10 [5.01-5.21]	6.03 [5.93-6.12]	7.17 [6.95-7.33]	10.18 [9.51-10.70]
74	3.44 [3.32-3.61]	4.67 [4.54-4.84]	5.50 [5.33-5.66]	6.47 [6.24-6.68]	8.85 [8.46-9.26]	3.78 [3.64-4.04]	5.11 [5.01-5.22]	6.03 [5.93-6.13]	7.17 [6.93-7.34]	10.18 [9.45-10.74]
75	3.43 [3.29-3.62]	4.67 [4.51-4.85]	5.49 [5.29-5.68]	6.46 [6.21-6.71]	8.85 [8.42-9.31]	3.79 [3.63-4.06]	5.11 [5.00-5.24]	6.03 [5.92-6.14]	7.17 [6.92-7.35]	10.18 [9.40-10.77]
76	3.43 [3.26-3.63]	4.66 [4.49-4.86]	5.48 [5.27-5.71]	6.46 [6.17-6.74]	8.86 [8.36-9.39]	3.79 [3.62-4.08]	5.11 [5.00-5.25]	6.03 [5.91-6.15]	7.17 [6.90-7.36]	10.18 [9.34-10.81]
77	3.41 [3.23-3.63]	4.65 [4.45-4.88]	5.48 [5.23-5.74]	6.46 [6.13-6.78]	8.86 [8.30-9.47]	3.79 [3.61-4.11]	5.11 [4.99-5.26]	6.03 [5.91-6.15]	7.17 [6.88-7.38]	10.19 [9.26-10.85]
78	3.40 [3.20-3.63]	4.64 [4.41-4.89]	5.47 [5.19-5.76]	6.45 [6.07-6.82]	8.87 [8.25-9.54]	3.79 [3.60-4.13]	5.11 [4.98-5.27]	6.03 [5.90-6.16]	7.17 [6.87-7.39]	10.19 [9.18-10.90]

79	3.39<br>[3.17-3.63]	4.63<br>[4.38-4.90]	5.46<br>[5.14-5.79]	6.44<br>[6.02-6.86]	8.87<br>[8.18-9.59]	3.80<br>[3.58-4.16]	5.11<br>[4.97-5.28]	6.04<br>[5.89-6.16]	7.17<br>[6.86-7.40]	10.19<br>[9.11-10.95]
80	3.38<br>[3.14-3.63]	4.62<br>[4.34-4.91]	5.45<br>[5.09-5.81]	6.44<br>[5.98-6.89]	8.87<br>[8.11-9.64]	3.80<br>[3.57-4.19]	5.12<br>[4.97-5.30]	6.04<br>[5.89-6.17]	7.18<br>[6.84-7.41]	10.19<br>[9.03-10.99]

Supplemental Table 7. Model-predicted sex- and age-specific next-generation reference intervals for PLT.

Age	Female					Male				
	Q2.5	Q25	Q50	Q75	Q97.5	Q2.5	Q25	Q50	Q75	Q97.5
18	163<br>[159-167]	215<br>[210-219]	247<br>[242-252]	283<br>[278-288]	363<br>[356-369]	152<br>[150-155]	197<br>[195-199]	225<br>[223-227]	256<br>[253-258]	324<br>[320-330]
19	162<br>[159-165]	214<br>[210-218]	246<br>[242-250]	282<br>[278-286]	362<br>[356-368]	152<br>[150-154]	197<br>[195-199]	225<br>[222-227]	256<br>[253-258]	325<br>[320-330]
20	161<br>[159-164]	213<br>[210-216]	245<br>[242-248]	281<br>[278-285]	362<br>[357-367]	151<br>[149-153]	197<br>[195-198]	224<br>[223-226]	256<br>[253-258]	325<br>[321-330]
21	161<br>[159-163]	212<br>[210-214]	245<br>[242-247]	281<br>[278-284]	361<br>[357-365]	151<br>[149-153]	196<br>[195-198]	224<br>[223-226]	256<br>[253-258]	326<br>[321-330]
22	160<br>[158-162]	212<br>[210-213]	244<br>[242-246]	280<br>[278-282]	361<br>[358-364]	151<br>[149-152]	196<br>[194-197]	224<br>[223-226]	256<br>[254-258]	326<br>[322-330]
23	159<br>[158-161]	211<br>[210-212]	243<br>[242-245]	280<br>[278-281]	360<br>[358-363]	150<br>[148-152]	196<br>[194-197]	224<br>[222-225]	255<br>[254-257]	326<br>[323-330]

24	159<br>[157-160]	211<br>[209-211]	243<br>[242-244]	279<br>[278-280]	360<br>[358-363]	150<br>[148-151]	195<br>[194-197]	224<br>[222-225]	255<br>[254-257]	327<br>[323-330]
25	158<br>[157-159]	210<br>[209-211]	242<br>[241-243]	279<br>[277-280]	360<br>[358-362]	149<br>[148-150]	195<br>[194-196]	223<br>[222-225]	255<br>[254-257]	327<br>[324-330]
26	157<br>[156-158]	209<br>[208-210]	242<br>[241-243]	278<br>[277-279]	360<br>[358-362]	149<br>[147-150]	195<br>[194-196]	223<br>[222-224]	255<br>[254-257]	327<br>[324-330]
27	157<br>[156-158]	209<br>[208-210]	241<br>[240-242]	278<br>[277-279]	360<br>[358-362]	148<br>[147-149]	194<br>[193-195]	223<br>[222-224]	255<br>[254-256]	328<br>[325-330]
28	156<br>[155-157]	208<br>[208-209]	241<br>[240-242]	277<br>[276-278]	359<br>[358-361]	148<br>[147-149]	194<br>[193-195]	223<br>[222-224]	255<br>[254-256]	328<br>[325-330]
29	156<br>[155-156]	208<br>[207-208]	240<br>[240-241]	277<br>[276-278]	359<br>[358-361]	147<br>[146-148]	194<br>[193-195]	223<br>[222-224]	255<br>[254-256]	328<br>[326-330]
30	155<br>[154-156]	207<br>[207-208]	240<br>[239-241]	277<br>[276-278]	359<br>[358-361]	147<br>[146-148]	193<br>[193-194]	223<br>[222-223]	255<br>[254-256]	329<br>[326-331]
31	154<br>[154-155]	207<br>[206-208]	240<br>[239-240]	276<br>[275-277]	359<br>[358-361]	147<br>[146-147]	193<br>[192-194]	222<br>[221-223]	255<br>[254-256]	329<br>[327-331]
32	154<br>[153-155]	206<br>[206-207]	239<br>[238-240]	276<br>[275-277]	359<br>[358-361]	146<br>[145-147]	193<br>[192-194]	222<br>[221-223]	255<br>[254-256]	330<br>[328-331]
33	153<br>[153-154]	206<br>[205-207]	239<br>[238-240]	276<br>[275-277]	359<br>[358-361]	146<br>[145-146]	193<br>[192-193]	222<br>[221-223]	255<br>[254-256]	330<br>[328-332]
34	153<br>[152-154]	206<br>[205-206]	238<br>[238-239]	276<br>[275-276]	359<br>[358-361]	145<br>[144-146]	192<br>[192-193]	222<br>[221-223]	255<br>[254-256]	330<br>[329-332]
35	153<br>[152-154]	205<br>[204-206]	238<br>[237-239]	275<br>[275-276]	360<br>[358-361]	145<br>[144-146]	192<br>[191-193]	222<br>[221-223]	255<br>[254-256]	331<br>[329-333]
36	152<br>[151-153]	205<br>[204-206]	238<br>[237-239]	275<br>[274-276]	360<br>[358-362]	144<br>[144-145]	192<br>[191-193]	222<br>[221-223]	255<br>[254-256]	331<br>[330-333]

37	152<br>[151-153]	205<br>[204-206]	238<br>[237-239]	276<br>[275-277]	360<br>[358-362]	144<br>[143-145]	192<br>[191-192]	222<br>[221-223]	255<br>[254-256]	332<br>[330-334]
38	152<br>[151-153]	205<br>[204-206]	238<br>[237-239]	276<br>[275-277]	361<br>[359-363]	144<br>[143-145]	191<br>[191-192]	221<br>[221-222]	255<br>[254-256]	332<br>[331-335]
39	152<br>[151-153]	205<br>[204-206]	239<br>[237-240]	276<br>[275-278]	362<br>[360-364]	143<br>[142-144]	191<br>[190-192]	221<br>[220-222]	255<br>[254-257]	333<br>[331-335]
40	152<br>[151-153]	205<br>[204-207]	239<br>[238-240]	277<br>[276-278]	363<br>[361-365]	143<br>[142-144]	191<br>[190-192]	221<br>[220-222]	255<br>[254-257]	333<br>[331-336]
41	152<br>[151-153]	206<br>[205-207]	240<br>[238-241]	278<br>[277-279]	365<br>[362-367]	142<br>[141-143]	190<br>[189-191]	221<br>[220-222]	255<br>[254-257]	334<br>[331-336]
42	152<br>[151-153]	206<br>[205-207]	240<br>[239-242]	279<br>[277-280]	366<br>[364-368]	142<br>[141-143]	190<br>[189-191]	221<br>[219-222]	255<br>[254-257]	334<br>[331-337]
43	152<br>[151-154]	207<br>[205-208]	241<br>[240-242]	280<br>[278-281]	367<br>[365-370]	141<br>[140-142]	190<br>[189-191]	220<br>[219-222]	255<br>[254-257]	334<br>[332-337]
44	152<br>[151-153]	207<br>[206-208]	241<br>[240-242]	280<br>[279-282]	368<br>[366-371]	141<br>[139-142]	189<br>[188-190]	220<br>[219-221]	255<br>[253-256]	335<br>[332-337]
45	152<br>[151-154]	207<br>[205-208]	241<br>[240-243]	280<br>[279-282]	369<br>[366-372]	140<br>[139-141]	189<br>[188-190]	220<br>[218-221]	255<br>[253-256]	335<br>[332-338]
46	152<br>[150-153]	206<br>[205-208]	241<br>[239-243]	280<br>[278-282]	369<br>[366-372]	139<br>[138-141]	188<br>[187-189]	219<br>[218-221]	255<br>[253-256]	335<br>[332-338]
47	151<br>[150-153]	206<br>[204-207]	240<br>[239-242]	279<br>[277-281]	368<br>[365-371]	139<br>[138-140]	188<br>[187-189]	219<br>[217-220]	254<br>[253-256]	335<br>[332-338]
48	150<br>[149-152]	205<br>[203-206]	239<br>[238-241]	278<br>[276-280]	366<br>[363-369]	138<br>[137-140]	187<br>[186-188]	218<br>[217-220]	254<br>[252-255]	335<br>[332-338]
49	149<br>[148-151]	203<br>[202-205]	238<br>[236-239]	276<br>[275-278]	364<br>[362-367]	137<br>[136-139]	187<br>[185-188]	218<br>[217-219]	254<br>[252-255]	335<br>[332-338]

50	148<br>[147-149]	202<br>[201-203]	236<br>[235-237]	275<br>[273-276]	362<br>[360-365]	137<br>[135-138]	186<br>[185-187]	217<br>[216-219]	253<br>[252-255]	335<br>[332-338]
51	147<br>[146-148]	201<br>[199-202]	234<br>[233-236]	273<br>[271-274]	360<br>[357-362]	136<br>[135-137]	185<br>[184-187]	217<br>[216-218]	253<br>[251-254]	335<br>[332-338]
52	146<br>[145-147]	199<br>[198-200]	233<br>[231-234]	271<br>[269-273]	357<br>[354-361]	135<br>[134-137]	185<br>[184-186]	216<br>[215-218]	252<br>[251-254]	335<br>[332-338]
53	145<br>[143-146]	198<br>[196-199]	231<br>[229-233]	269<br>[267-271]	355<br>[352-358]	135<br>[133-136]	184<br>[183-185]	216<br>[214-217]	252<br>[251-254]	335<br>[332-338]
54	144<br>[142-145]	196<br>[195-198]	230<br>[228-232]	267<br>[265-270]	353<br>[350-357]	134<br>[133-135]	183<br>[182-185]	215<br>[214-216]	251<br>[250-253]	335<br>[332-338]
55	143<br>[141-145]	195<br>[194-197]	228<br>[227-231]	266<br>[264-269]	352<br>[348-355]	133<br>[132-135]	183<br>[181-184]	215<br>[213-216]	251<br>[249-253]	335<br>[332-339]
56	142<br>[140-144]	195<br>[193-196]	228<br>[226-230]	265<br>[263-268]	351<br>[347-354]	132<br>[131-134]	182<br>[181-183]	214<br>[213-215]	250<br>[249-252]	335<br>[331-339]
57	142<br>[140-144]	194<br>[192-196]	227<br>[225-229]	265<br>[262-267]	350<br>[346-354]	132<br>[130-133]	181<br>[180-183]	213<br>[212-215]	250<br>[248-252]	335<br>[331-339]
58	141<br>[140-143]	194<br>[192-195]	227<br>[225-229]	264<br>[262-267]	350<br>[345-353]	131<br>[129-133]	181<br>[179-182]	213<br>[211-214]	249<br>[248-251]	334<br>[331-339]
59	141<br>[139-143]	193<br>[192-195]	226<br>[225-228]	264<br>[262-266]	350<br>[346-353]	130<br>[128-132]	180<br>[178-181]	212<br>[210-214]	249<br>[247-251]	334<br>[330-339]
60	141<br>[139-143]	193<br>[191-195]	226<br>[224-229]	264<br>[262-266]	350<br>[346-353]	129<br>[127-131]	179<br>[177-181]	211<br>[209-213]	248<br>[246-250]	334<br>[330-339]
61	141<br>[138-143]	193<br>[191-196]	226<br>[224-229]	264<br>[262-267]	350<br>[346-354]	128<br>[126-131]	178<br>[176-180]	211<br>[209-212]	248<br>[245-250]	334<br>[330-339]
62	141<br>[138-143]	193<br>[191-196]	226<br>[224-229]	264<br>[262-268]	350<br>[346-355]	128<br>[125-130]	178<br>[176-179]	210<br>[208-212]	247<br>[245-249]	333<br>[329-339]

63	141<br>[138-143]	193<br>[190-196]	226<br>[224-230]	264<br>[261-268]	351<br>[346-356]	127<br>[124-129]	177<br>[175-179]	209<br>[207-211]	246<br>[244-249]	333<br>[329-338]
64	140<br>[137-143]	193<br>[190-196]	226<br>[223-230]	264<br>[261-268]	350<br>[346-356]	126<br>[124-128]	176<br>[174-178]	208<br>[206-211]	246<br>[243-248]	332<br>[328-338]
65	140<br>[137-143]	192<br>[190-196]	226<br>[223-230]	264<br>[260-268]	350<br>[345-356]	125<br>[123-128]	175<br>[173-177]	208<br>[205-210]	245<br>[242-248]	332<br>[327-338]
66	140<br>[136-143]	192<br>[189-196]	225<br>[222-229]	263<br>[259-267]	349<br>[344-356]	124<br>[122-127]	174<br>[172-177]	207<br>[205-210]	244<br>[242-248]	332<br>[326-337]
67	139<br>[135-142]	191<br>[188-195]	224<br>[221-229]	262<br>[258-266]	348<br>[343-355]	124<br>[121-126]	174<br>[171-176]	206<br>[204-209]	244<br>[241-247]	331<br>[326-337]
68	138<br>[135-142]	190<br>[187-194]	223<br>[220-228]	261<br>[257-266]	347<br>[341-354]	123<br>[120-126]	173<br>[170-176]	205<br>[203-209]	243<br>[240-247]	331<br>[325-337]
69	138<br>[134-141]	189<br>[186-193]	222<br>[219-226]	260<br>[256-265]	346<br>[339-353]	122<br>[119-125]	172<br>[169-175]	205<br>[202-208]	242<br>[239-246]	330<br>[323-337]
70	137<br>[133-141]	188<br>[185-192]	221<br>[217-225]	259<br>[254-263]	344<br>[337-351]	121<br>[118-125]	171<br>[168-174]	204<br>[201-208]	241<br>[238-246]	329<br>[323-337]
71	136<br>[132-140]	187<br>[183-191]	220<br>[216-224]	257<br>[252-262]	342<br>[334-351]	121<br>[117-125]	170<br>[167-174]	203<br>[200-207]	241<br>[237-245]	329<br>[322-337]
72	135<br>[130-140]	186<br>[182-191]	219<br>[214-223]	256<br>[250-261]	341<br>[332-350]	120<br>[116-124]	170<br>[166-173]	202<br>[199-206]	240<br>[236-245]	328<br>[321-337]
73	134<br>[129-139]	185<br>[181-190]	217<br>[212-223]	254<br>[248-260]	339<br>[329-348]	119<br>[115-124]	169<br>[165-173]	201<br>[197-206]	239<br>[235-245]	328<br>[320-337]
74	133<br>[128-138]	183<br>[179-189]	216<br>[210-222]	252<br>[246-259]	336<br>[326-347]	118<br>[115-123]	168<br>[164-172]	201<br>[197-205]	238<br>[233-244]	327<br>[319-337]
75	132<br>[127-137]	182<br>[177-188]	214<br>[208-221]	251<br>[243-258]	334<br>[324-345]	117<br>[114-123]	167<br>[163-172]	200<br>[196-205]	238<br>[233-244]	326<br>[316-337]

76	131<br>[125-136]	181<br>[174-188]	213<br>[206-220]	249<br>[241-258]	332<br>[321-344]	117<br>[113-122]	166<br>[162-171]	199<br>[194-204]	237<br>[231-243]	326<br>[313-337]
77	130<br>[124-136]	179<br>[173-187]	211<br>[204-220]	247<br>[238-257]	330<br>[317-343]	116<br>[112-121]	166<br>[161-171]	198<br>[193-203]	236<br>[230-243]	325<br>[311-337]
78	128<br>[122-135]	178<br>[170-186]	209<br>[201-219]	245<br>[235-256]	327<br>[312-342]	115<br>[111-121]	165<br>[160-171]	197<br>[192-203]	235<br>[229-242]	325<br>[308-337]
79	127<br>[120-135]	176<br>[168-185]	208<br>[199-218]	243<br>[232-256]	325<br>[308-341]	114<br>[110-120]	164<br>[159-170]	196<br>[191-202]	234<br>[228-242]	324<br>[306-337]
80	126<br>[119-135]	175<br>[166-184]	206<br>[196-217]	241<br>[229-255]	323<br>[305-340]	114<br>[109-120]	163<br>[158-170]	196<br>[190-202]	234<br>[227-241]	323<br>[305-337]
