# Supplementary material for: Establishment and Temporal Validation of Next-Generation Reference Intervals for Routine Hematological Parameters Using Large-Scale Data
Source: Diagnostics (Basel). 2026 Mar 23;16(6):944. doi: 10.3390/diagnostics16060944 (PMC13025947; doi:10.3390/diagnostics16060944)
Supplement: Supplementary file 1 [file diagnostics-16-00944-s001.zip › Supplemental Table 8-14.pdf]

Supplemental Table 8. External validation of the next-generation reference interval for HGB across annual validation cohorts (2019–2023).

Year	Group	NRI	n	Total	Percent (%)
2019	Female	L	354	10712	3.30
2019	Female	N	10113	10712	94.41
2019	Female	U	245	10712	2.29
2019	Male	L	180	7184	2.51
2019	Male	N	6877	7184	95.73
2019	Male	U	127	7184	1.77
2020	Female	L	215	5512	3.90
2020	Female	N	5173	5512	93.85
2020	Female	U	124	5512	2.25
2020	Male	L	90	3722	2.42
2020	Male	N	3569	3722	95.89
2020	Male	U	63	3722	1.69
2021	Female	L	330	8883	3.71
2021	Female	N	8367	8883	94.19
2021	Female	U	186	8883	2.09
2021	Male	L	146	5688	2.57
2021	Male	N	5455	5688	95.90
2021	Male	U	87	5688	1.53
2022	Female	L	290	8259	3.51
2022	Female	N	7716	8259	93.43
2022	Female	U	253	8259	3.06
2022	Male	L	121	5167	2.34
2022	Male	N	4932	5167	95.45
2022	Male	U	114	5167	2.21
2023	Female	L	358	9283	3.86
2023	Female	N	8728	9283	94.02
2023	Female	U	197	9283	2.12
2023	Male	L	164	5554	2.95
2023	Male	N	5283	5554	95.12
2023	Male	U	107	5554	1.93

Supplemental Table 9.– External validation of the next-generation reference interval for HCT across annual validation cohorts (2019–2023).

Year	Group	NRI	n	Total	Percent (%)
2019	Female	L	362	10712	3.38
2019	Female	N	10134	10712	94.60
2019	Female	U	216	10712	2.02
2019	Male	L	273	7184	3.80
2019	Male	N	6811	7184	94.81
2019	Male	U	100	7184	1.39
2020	Female	L	212	5512	3.85
2020	Female	N	5211	5512	94.54
2020	Female	U	89	5512	1.61
2020	Male	L	171	3722	4.59
2020	Male	N	3513	3722	94.38
2020	Male	U	38	3722	1.02
2021	Female	L	323	8883	3.64
2021	Female	N	8400	8883	94.56
2021	Female	U	160	8883	1.80
2021	Male	L	251	5688	4.41
2021	Male	N	5364	5688	94.30
2021	Male	U	73	5688	1.28
2022	Female	L	270	8259	3.27
2022	Female	N	7813	8259	94.60
2022	Female	U	176	8259	2.13
2022	Male	L	168	5167	3.25
2022	Male	N	4932	5167	95.45
2022	Male	U	67	5167	1.30
2023	Female	L	296	9283	3.19
2023	Female	N	8711	9283	93.84
2023	Female	U	276	9283	2.97
2023	Male	L	206	5554	3.71
2023	Male	N	5200	5554	93.63
2023	Male	U	148	5554	2.66

Supplemental Table 10. External validation of the next-generation reference interval for MCV across annual validation cohorts (2019–2023).

Year	Group	NRI	n	Total	Percent (%)

2019	Female	L	370	10712	3.45
2019	Female	N	10067	10712	93.98
2019	Female	U	275	10712	2.57
2019	Male	L	112	7184	1.56
2019	Male	N	6923	7184	96.37
2019	Male	U	149	7184	2.07
2020	Female	L	227	5512	4.12
2020	Female	N	5167	5512	93.74
2020	Female	U	118	5512	2.14
2020	Male	L	56	3722	1.50
2020	Male	N	3611	3722	97.02
2020	Male	U	55	3722	1.48
2021	Female	L	358	8883	4.03
2021	Female	N	8372	8883	94.25
2021	Female	U	153	8883	1.72
2021	Male	L	103	5688	1.81
2021	Male	N	5509	5688	96.85
2021	Male	U	76	5688	1.34
2022	Female	L	350	8259	4.24
2022	Female	N	7781	8259	94.21
2022	Female	U	128	8259	1.55
2022	Male	L	94	5167	1.82
2022	Male	N	5018	5167	97.12
2022	Male	U	55	5167	1.06
2023	Female	L	310	9283	3.34
2023	Female	N	8725	9283	93.99
2023	Female	U	248	9283	2.67
2023	Male	L	84	5554	1.51
2023	Male	N	5384	5554	96.94
2023	Male	U	86	5554	1.55

Supplemental Table 11. External validation of the next-generation reference interval for –MCH across annual validation cohorts (2019–2023).

Year	Group	NRI	n	Total	Percent (%)
2019	Female	L	411	10712	3.84
2019	Female	N	9961	10712	92.99

2019	Female	U	340	10712	3.17
2019	Male	L	101	7184	1.41
2019	Male	N	6908	7184	96.16
2019	Male	U	175	7184	2.44
2020	Female	L	240	5512	4.35
2020	Female	N	5077	5512	92.11
2020	Female	U	195	5512	3.54
2020	Male	L	55	3722	1.48
2020	Male	N	3524	3722	94.68
2020	Male	U	143	3722	3.84
2021	Female	L	413	8883	4.65
2021	Female	N	8264	8883	93.03
2021	Female	U	206	8883	2.32
2021	Male	L	108	5688	1.90
2021	Male	N	5430	5688	95.46
2021	Male	U	150	5688	2.64
2022	Female	L	364	8259	4.41
2022	Female	N	7644	8259	92.55
2022	Female	U	251	8259	3.04
2022	Male	L	94	5167	1.82
2022	Male	N	4929	5167	95.39
2022	Male	U	144	5167	2.79
2023	Female	L	406	9283	4.37
2023	Female	N	8687	9283	93.58
2023	Female	U	190	9283	2.05
2023	Male	L	117	5554	2.11
2023	Male	N	5330	5554	95.97
2023	Male	U	107	5554	1.93

Supplemental Table 12. External validation of the next-generation reference interval for MCHC across annual validation cohorts (2019–2023).

Year	Group	NRI	n	Total	Percent (%)
2019	Female	L	245	10712	2.29
2019	Female	N	10185	10712	95.08
2019	Female	U	282	10712	2.63
2019	Male	L	75	7184	1.04

2019	Male	N	6917	7184	96.28
2019	Male	U	192	7184	2.67
2020	Female	L	169	5512	3.07
2020	Female	N	5023	5512	91.13
2020	Female	U	320	5512	5.81
2020	Male	L	43	3722	1.16
2020	Male	N	3421	3722	91.91
2020	Male	U	258	3722	6.93
2021	Female	L	289	8883	3.25
2021	Female	N	8229	8883	92.64
2021	Female	U	365	8883	4.11
2021	Male	L	86	5688	1.51
2021	Male	N	5325	5688	93.62
2021	Male	U	277	5688	4.87
2022	Female	L	228	8259	2.76
2022	Female	N	7608	8259	92.12
2022	Female	U	423	8259	5.12
2022	Male	L	68	5167	1.32
2022	Male	N	4801	5167	92.92
2022	Male	U	298	5167	5.77
2023	Female	L	441	9283	4.75
2023	Female	N	8690	9283	93.61
2023	Female	U	152	9283	1.64
2023	Male	L	210	5554	3.78
2023	Male	N	5234	5554	94.24
2023	Male	U	110	5554	1.98

Supplemental Table 13-. External validation of the next-generation reference interval for WBC across annual validation cohorts (2019–2023).–

Year	Group	NRI	n	Total	Percent (%)
2019	Female	L	374	10712	3.49
2019	Female	N	10115	10712	94.43
2019	Female	U	223	10712	2.08
2019	Male	L	310	7184	4.32
2019	Male	N	6746	7184	93.90

2019	Male	U	128	7184	1.78
2020	Female	L	195	5512	3.54
2020	Female	N	5214	5512	94.59
2020	Female	U	103	5512	1.87
2020	Male	L	175	3722	4.70
2020	Male	N	3494	3722	93.87
2020	Male	U	53	3722	1.42
2021	Female	L	340	8883	3.83
2021	Female	N	8396	8883	94.52
2021	Female	U	147	8883	1.65
2021	Male	L	354	5688	6.22
2021	Male	N	5269	5688	92.63
2021	Male	U	65	5688	1.14
2022	Female	L	257	8259	3.11
2022	Female	N	7833	8259	94.84
2022	Female	U	169	8259	2.05
2022	Male	L	217	5167	4.20
2022	Male	N	4889	5167	94.62
2022	Male	U	61	5167	1.18
2023	Female	L	326	9283	3.51
2023	Female	N	8773	9283	94.51
2023	Female	U	184	9283	1.98
2023	Male	L	308	5554	5.55
2023	Male	N	5179	5554	93.25
2023	Male	U	67	5554	1.21

Supplemental Table 14. External validation of the next-generation reference interval for PLT across annual validation cohorts (2019–2023)

Year	Group	NRI	n	Total	Percent (%)
2019	Female	L	183	10710	1.71
2019	Female	N	10147	10710	94.74
2019	Female	U	380	10710	3.55
2019	Male	L	108	7184	1.50
2019	Male	N	6819	7184	94.92
2019	Male	U	257	7184	3.58
2020	Female	L	127	5511	2.30

2020	Female	N	5218	5511	94.68
2020	Female	U	166	5511	3.01
2020	Male	L	104	3722	2.79
2020	Male	N	3530	3722	94.84
2020	Male	U	88	3722	2.36
2021	Female	L	191	8883	2.15
2021	Female	N	8431	8883	94.91
2021	Female	U	261	8883	2.94
2021	Male	L	127	5687	2.23
2021	Male	N	5392	5687	94.81
2021	Male	U	168	5687	2.95
2022	Female	L	147	8259	1.78
2022	Female	N	7811	8259	94.58
2022	Female	U	301	8259	3.64
2022	Male	L	87	5167	1.68
2022	Male	N	4887	5167	94.58
2022	Male	U	193	5167	3.74
2023	Female	L	133	9282	1.43
2023	Female	N	8787	9282	94.67
2023	Female	U	362	9282	3.90
2023	Male	L	72	5554	1.30
2023	Male	N	5248	5554	94.49
2023	Male	U	234	5554	4.21
