# Supplementary figures and images for: Establishment and Temporal Validation of Next-Generation Reference Intervals for Routine Hematological Parameters Using Large-Scale Data
Source: Diagnostics (Basel). 2026 Mar 23;16(6):944. doi: 10.3390/diagnostics16060944 (PMC13025947; doi:10.3390/diagnostics16060944)

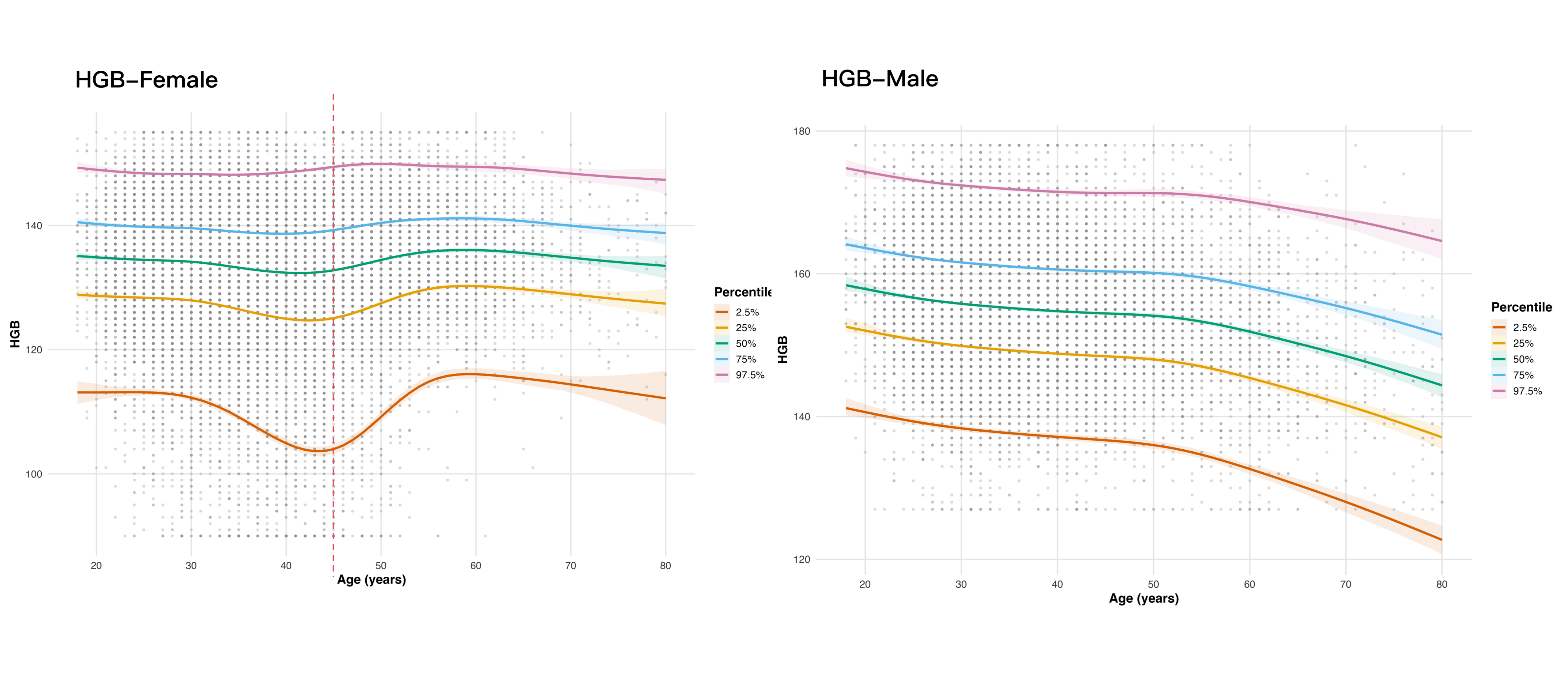

Supplement: Supplementary file 1 [file diagnostics-16-00944-s001.zip › Supplemental Figure 1.png]

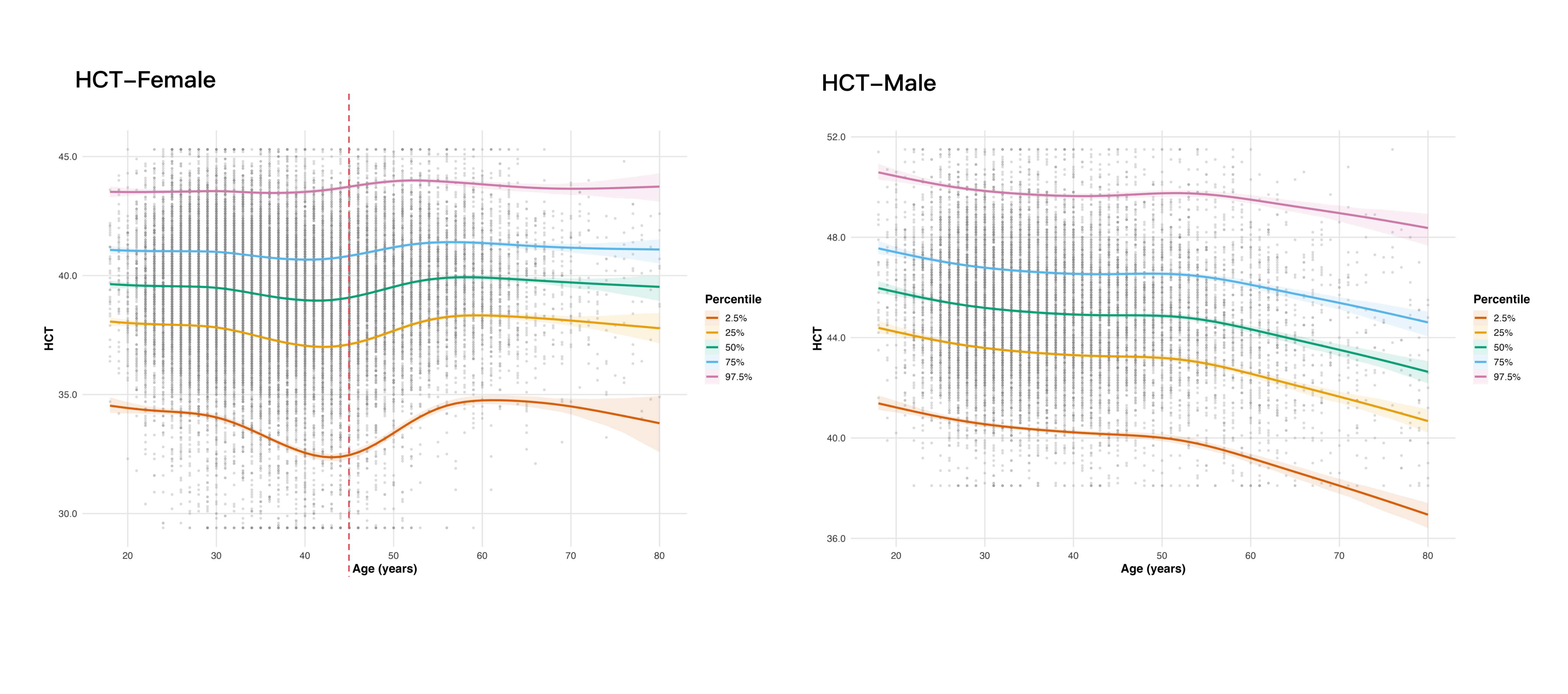

Supplement: Supplementary file 1 [file diagnostics-16-00944-s001.zip › Supplemental Figure 2.png]

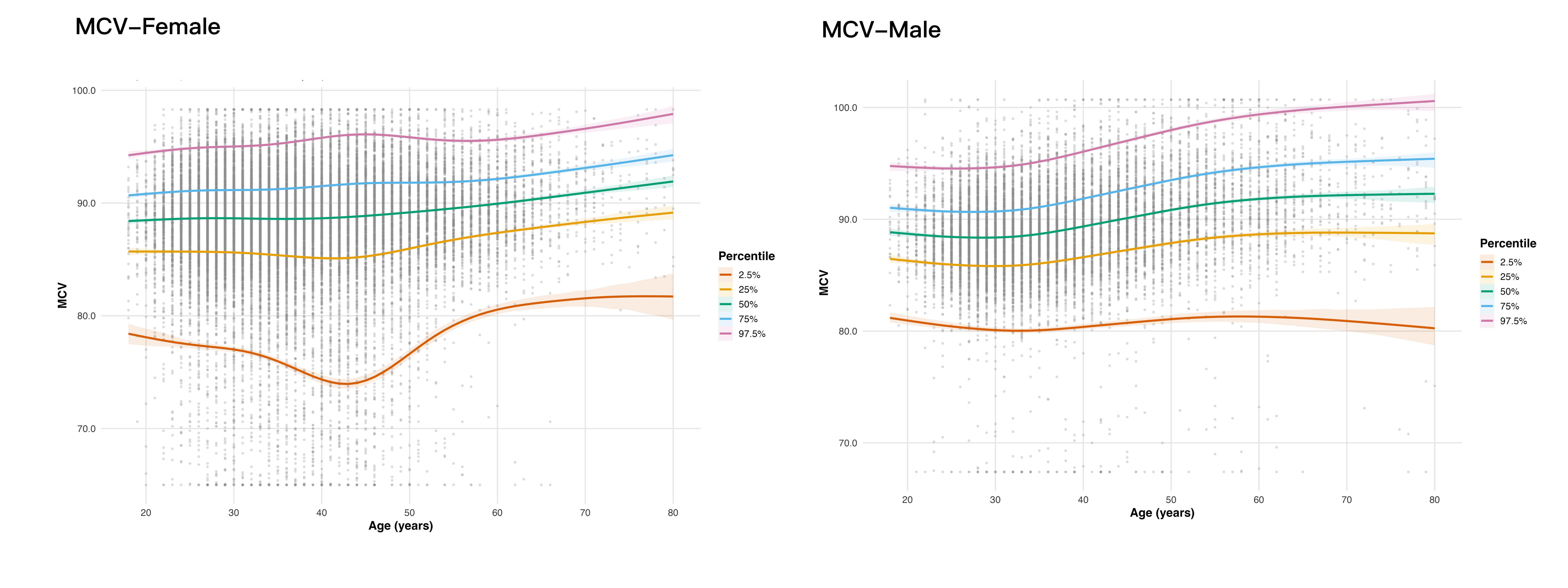

Supplement: Supplementary file 1 [file diagnostics-16-00944-s001.zip › Supplemental Figure 3.png]

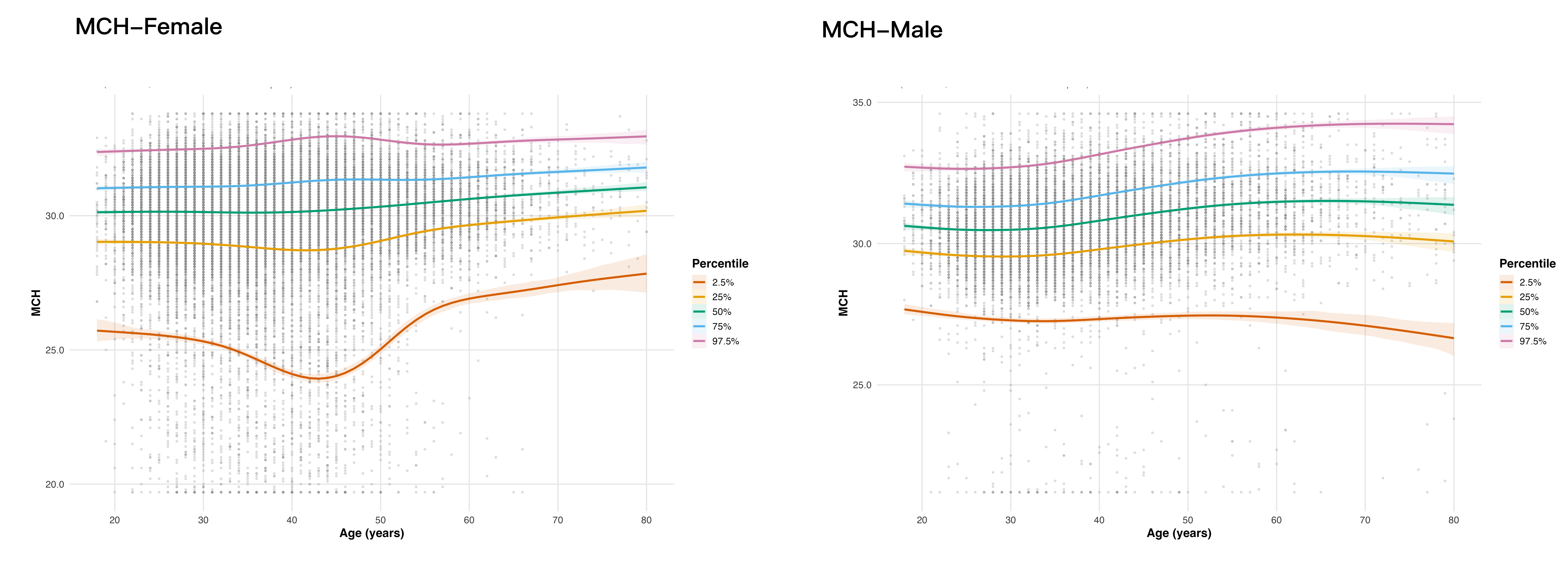

Supplement: Supplementary file 1 [file diagnostics-16-00944-s001.zip › Supplemental Figure 4.png]

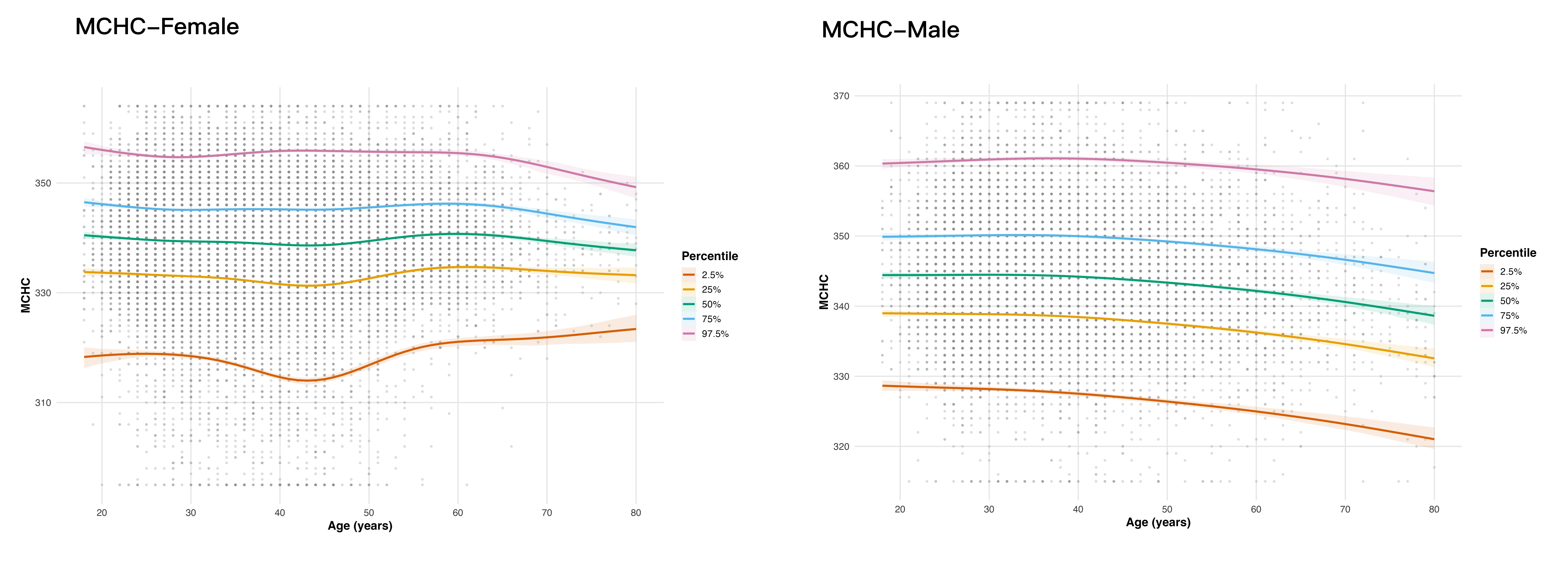

Supplement: Supplementary file 1 [file diagnostics-16-00944-s001.zip › Supplemental Figure 5.png]

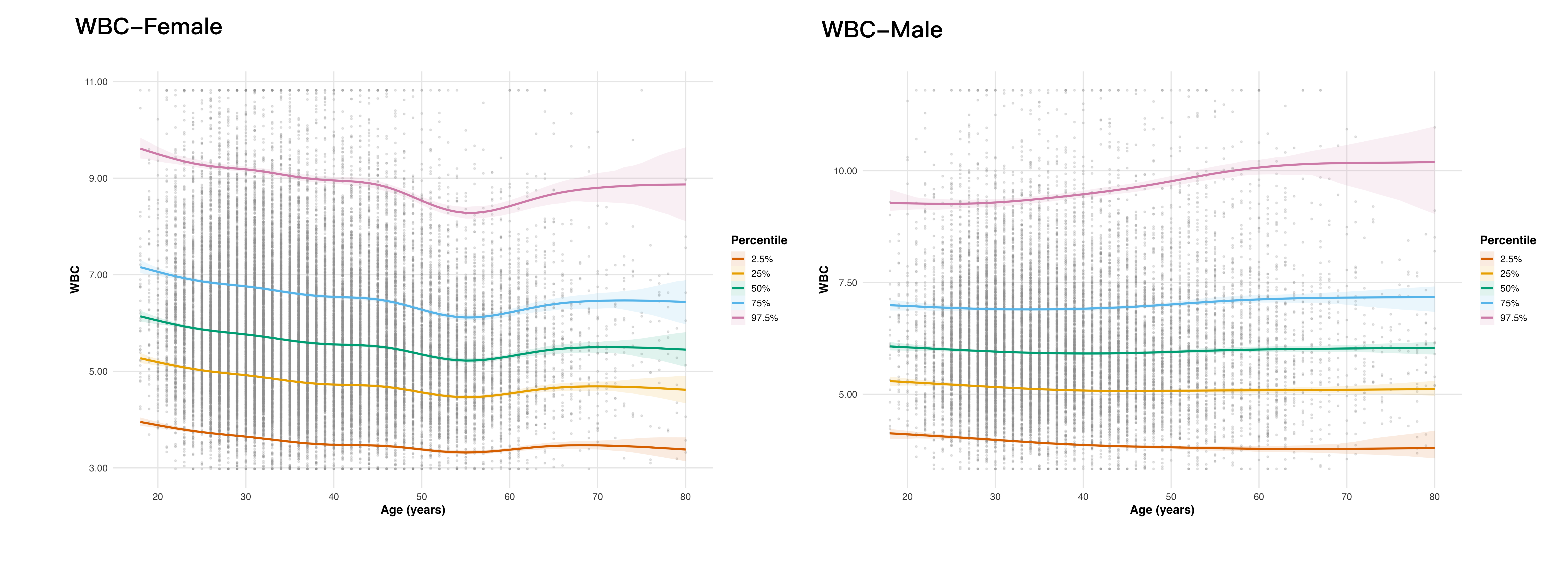

Supplement: Supplementary file 1 [file diagnostics-16-00944-s001.zip › Supplemental Figure 6.png]

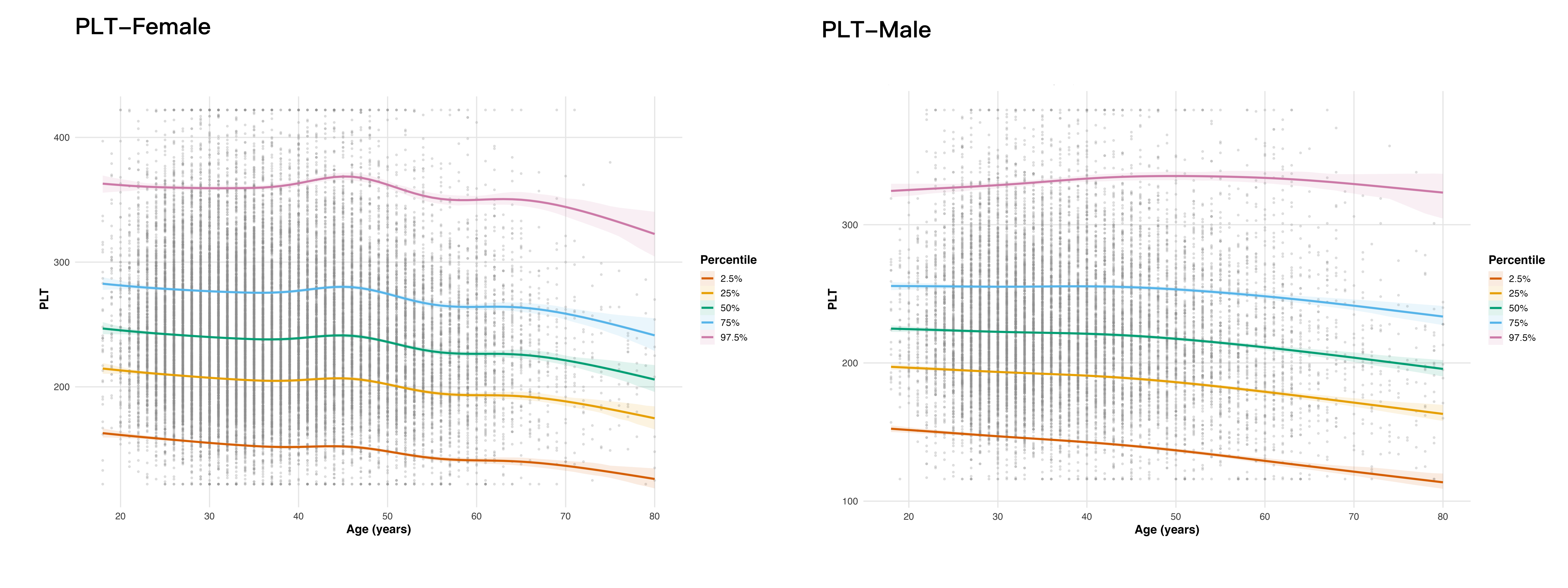

Supplement: Supplementary file 1 [file diagnostics-16-00944-s001.zip › Supplemental Figure 7.png]

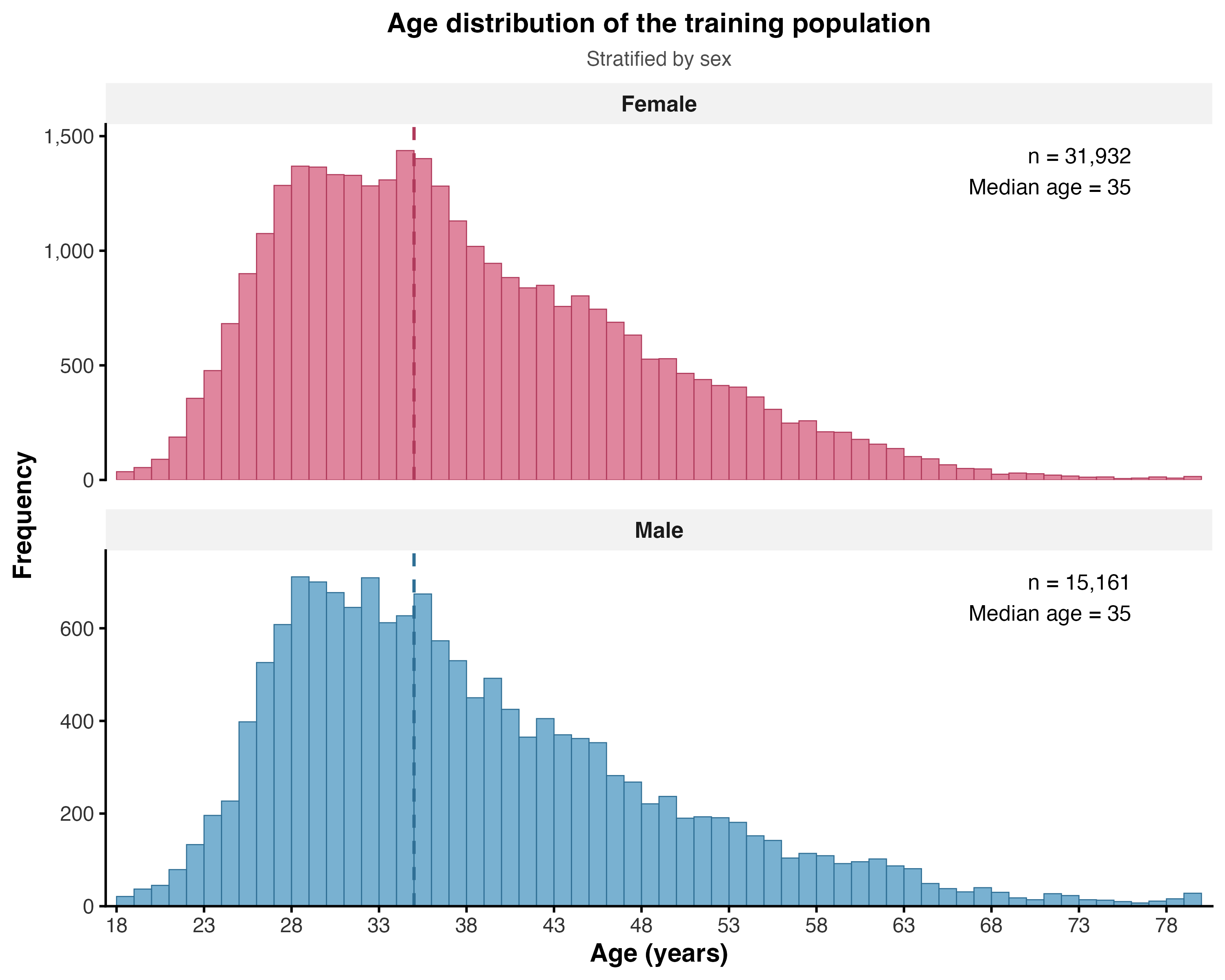

Supplement: Supplementary file 1 [file diagnostics-16-00944-s001.zip › Supplemental Figure 8.png]
